# Supplementary material for: The Phytochemical Profile of the Petroleum Ether Extract of Purslane Leaves and Its Anticancer Effect on 4-(Methylnitrosamino)-1-(3-pyridyl)-1-buta-4 None (NNK)-Induced Lung Cancer in Rats
Source: Int J Mol Sci. 2024 Dec 4;25(23):13024. doi: 10.3390/ijms252313024 (PMC11641252; doi:10.3390/ijms252313024)
Supplement: Supplementary file 1 [file ijms-25-13024-s001.zip › ijms-3271464-supplementary.pdf]

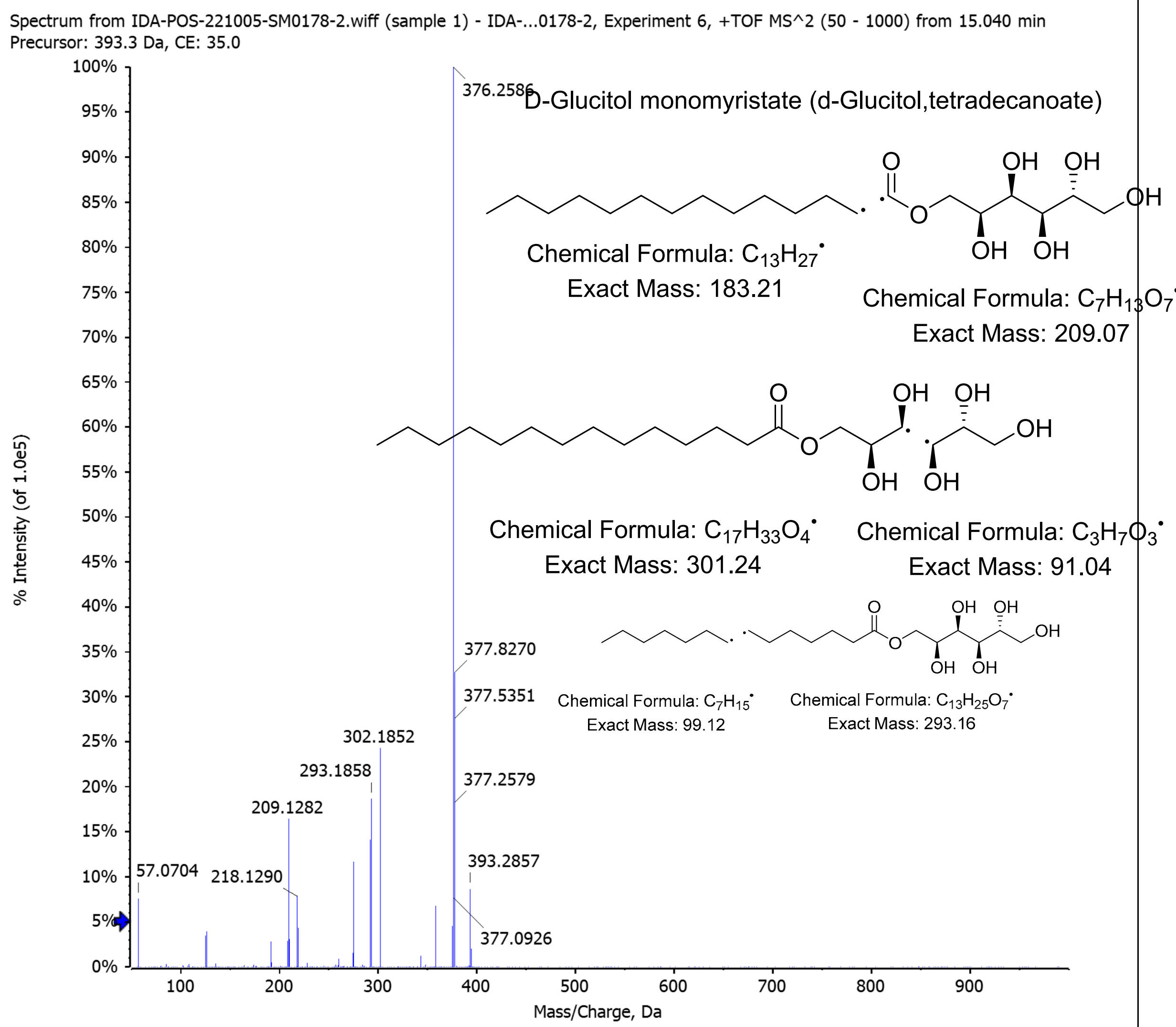
Spectrum from IDA-POS-221005-SM0178-2.wiff (sample 1) - IDA-...0178-2, Experiment 6, +TOF MS^2 (50 - 1000) from 15.040 min
Precursor: 393.3 Da, CE: 35.0
D-Glucitol monomyristate (d-Glucitol,tetradecanoate)
Chemical Formula: C13H27•
Exact Mass: 183.21
Chemical Formula: C7H13O7•
Exact Mass: 209.07
Chemical Formula: C17H33O4•
Exact Mass: 301.24
Chemical Formula: C3H7O3•
Exact Mass: 91.04
Chemical Formula: C7H15•
Exact Mass: 99.12
Chemical Formula: C13H25O7•
Exact Mass: 293.16
376.2586
377.8270
377.5351
377.2579
302.1852
293.1858
209.1282
218.1290
57.0704
393.2857
377.0926
% Intensity (of 1.0e5)
Mass/Charge, Da

Figure S1

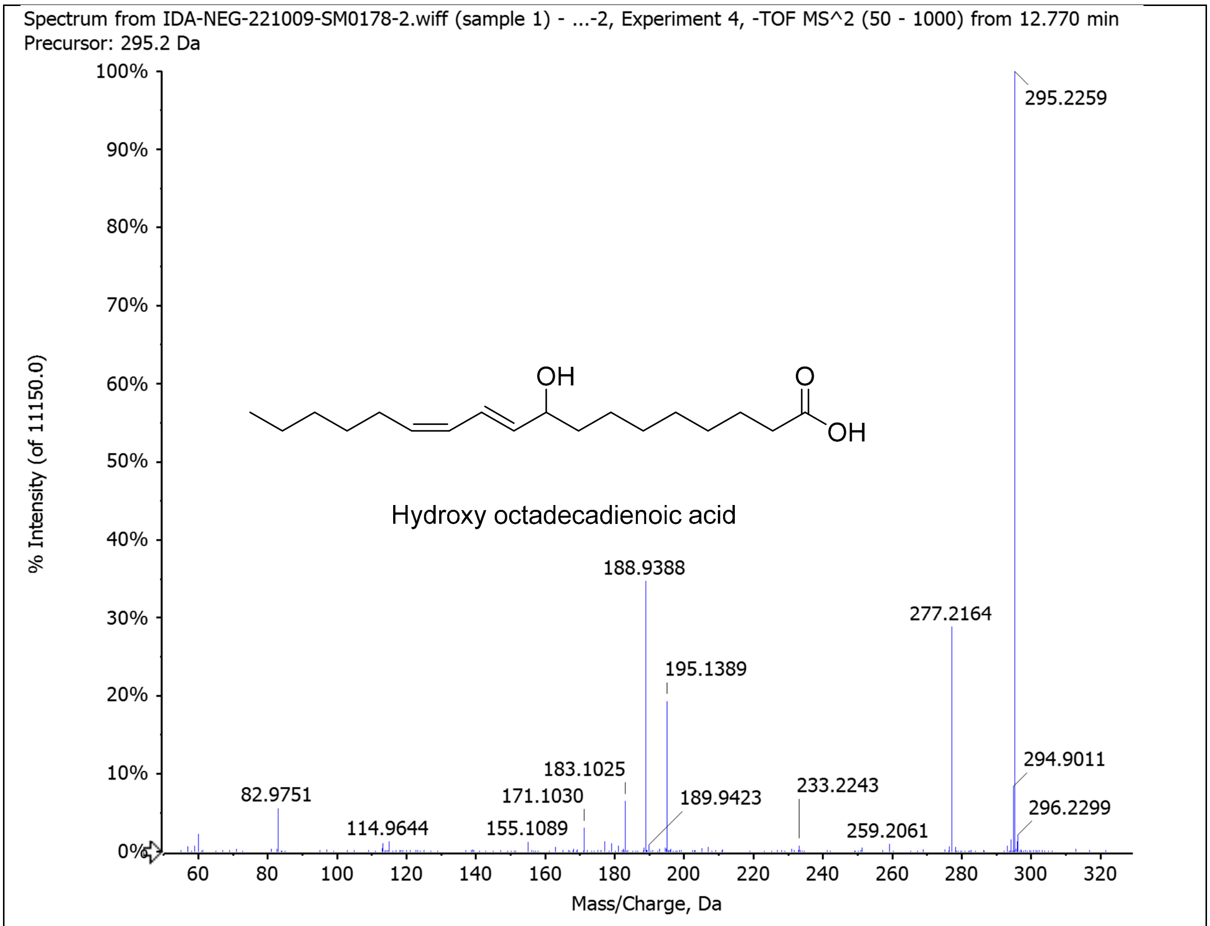

Spectrum from IDA-NEG-221009-SM0178-2.wiff (sample 1) - ...-2, Experiment 4, -TOF MS^2 (50 - 1000) from 12.770 min
Precursor: 295.2 Da
% Intensity (of 11150.0)
Mass/Charge, Da
OH
O
OH
Hydroxy octadecadienoic acid
295.2259
188.9388
277.2164
195.1389
294.9011
183.1025
233.2243
82.9751
171.1030
189.9423
296.2299
114.9644
155.1089
259.2061

Figure S2

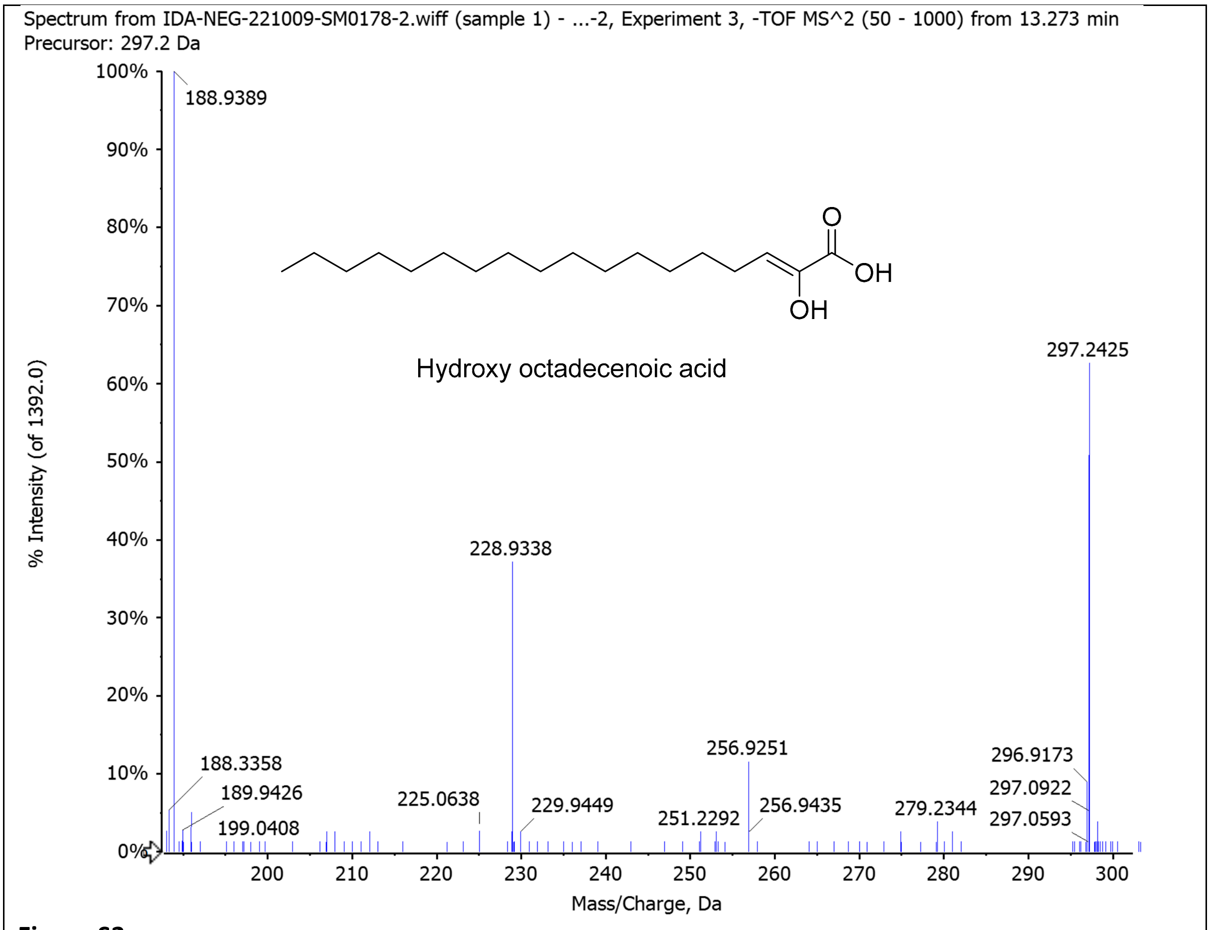

Spectrum from IDA-NEG-221009-SM0178-2.wiff (sample 1) - ...-2, Experiment 3, -TOF MS^2 (50 - 1000) from 13.273 min
Precursor: 297.2 Da
Hydroxy octadecenoic acid
OH
OH
O
188.9389
228.9338
297.2425
188.3358
189.9426
199.0408
225.0638
229.9449
251.2292
256.9251
256.9435
279.2344
296.9173
297.0922
297.0593
% Intensity (of 1392.0)
Mass/Charge, Da

Figure S3

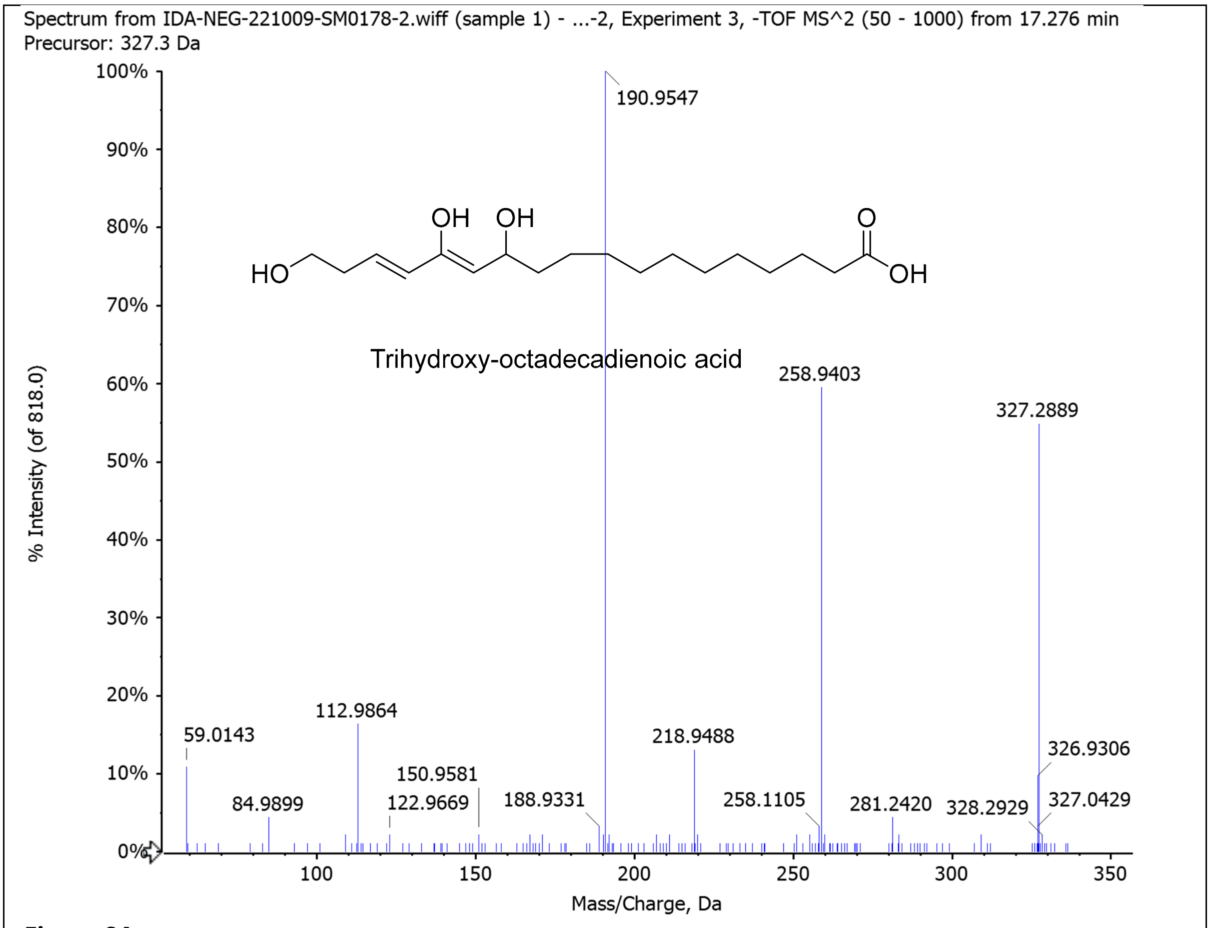
Spectrum from IDA-NEG-221009-SM0178-2.wiff (sample 1) - ...-2, Experiment 3, -TOF MS^2 (50 - 1000) from 17.276 min
Precursor: 327.3 Da
Trihydroxy-octadecadienoic acid
% Intensity (of 818.0)
Mass/Charge, Da
190.9547
258.9403
327.2889
112.9864
59.0143
218.9488
326.9306
150.9581
84.9899
122.9669
188.9331
258.1105
281.2420
328.2929
327.0429
HO
OH
OH
O
OH

Figure S4

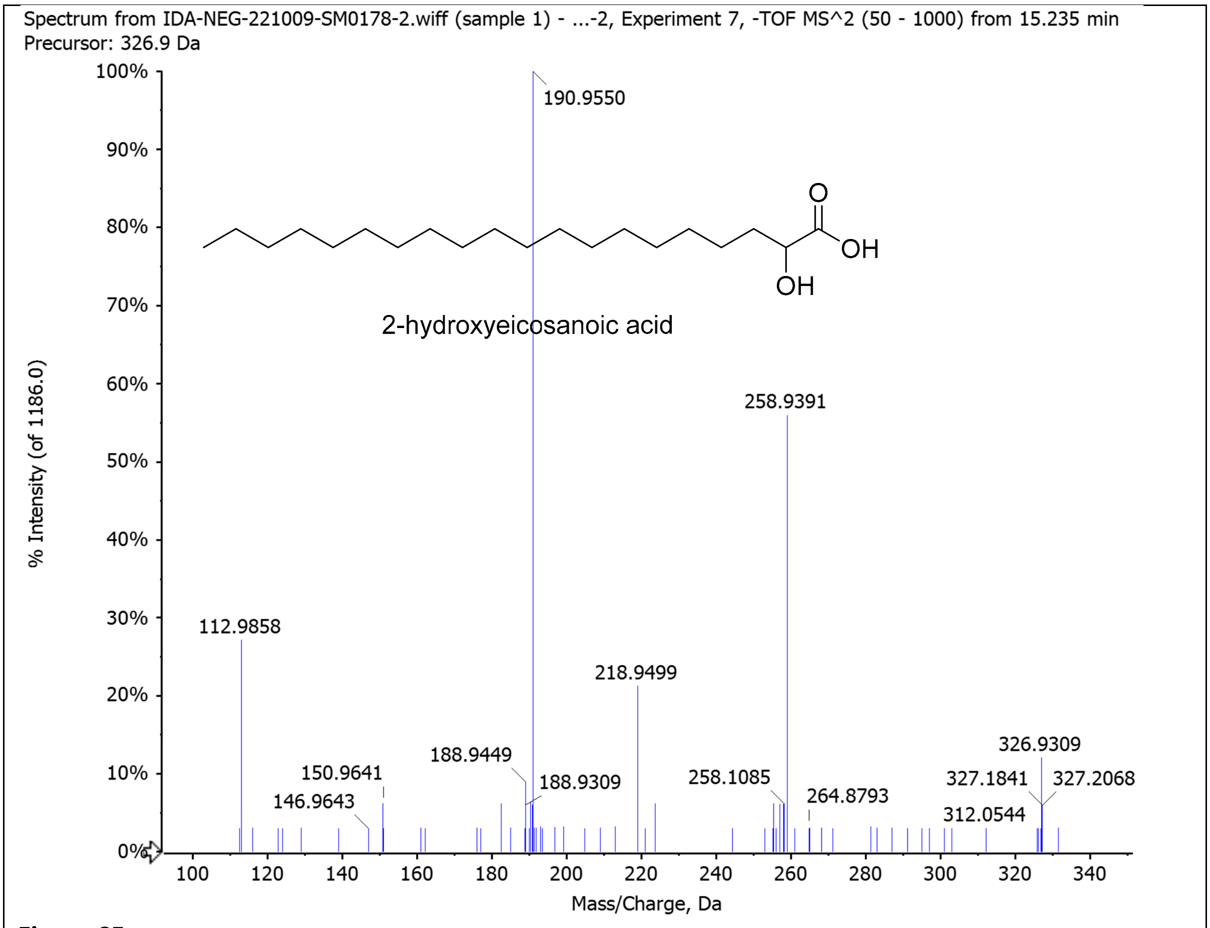
Spectrum from IDA-NEG-221009-SM0178-2.wiff (sample 1) - ...-2, Experiment 7, -TOF MS^2 (50 - 1000) from 15.235 min
Precursor: 326.9 Da
% Intensity (of 1186.0)
100%
90%
80%
70%
60%
50%
40%
30%
20%
10%
0%
190.9550
O
OH
OH
2-hydroxyeicosanoic acid
258.9391
112.9858
218.9499
188.9449
150.9641
146.9643
188.9309
258.1085
264.8793
326.9309
327.1841
327.2068
312.0544
100
120
140
160
180
200
220
240
260
280
300
320
340
Mass/Charge, Da

Figure S5

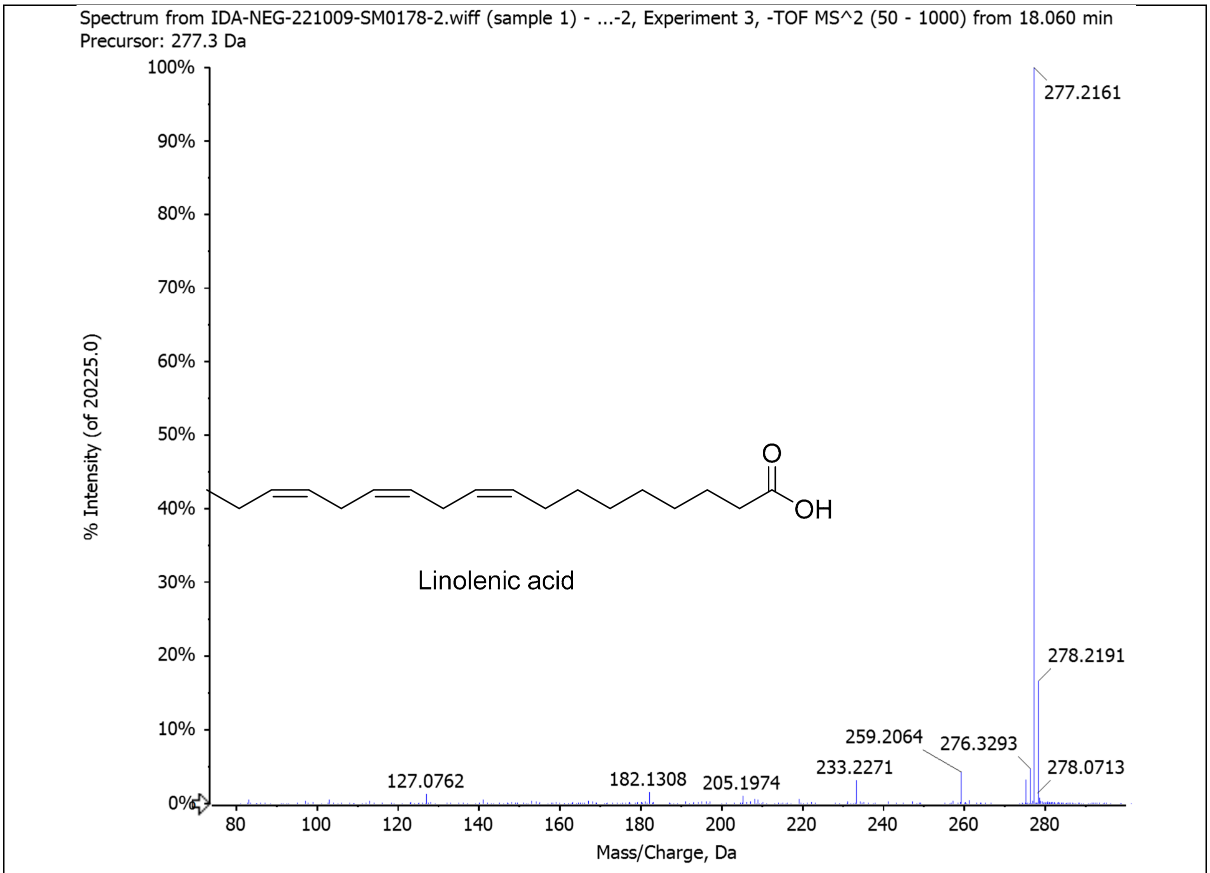
Spectrum from IDA-NEG-221009-SM0178-2.wiff (sample 1) - ...-2, Experiment 3, -TOF MS^2 (50 - 1000) from 18.060 min
Precursor: 277.3 Da
% Intensity (of 20225.0)
Mass/Charge, Da
277.2161
278.2191
259.2064
276.3293
278.0713
233.2271
127.0762
182.1308
205.1974
O
OH
Linolenic acid

Figure S6

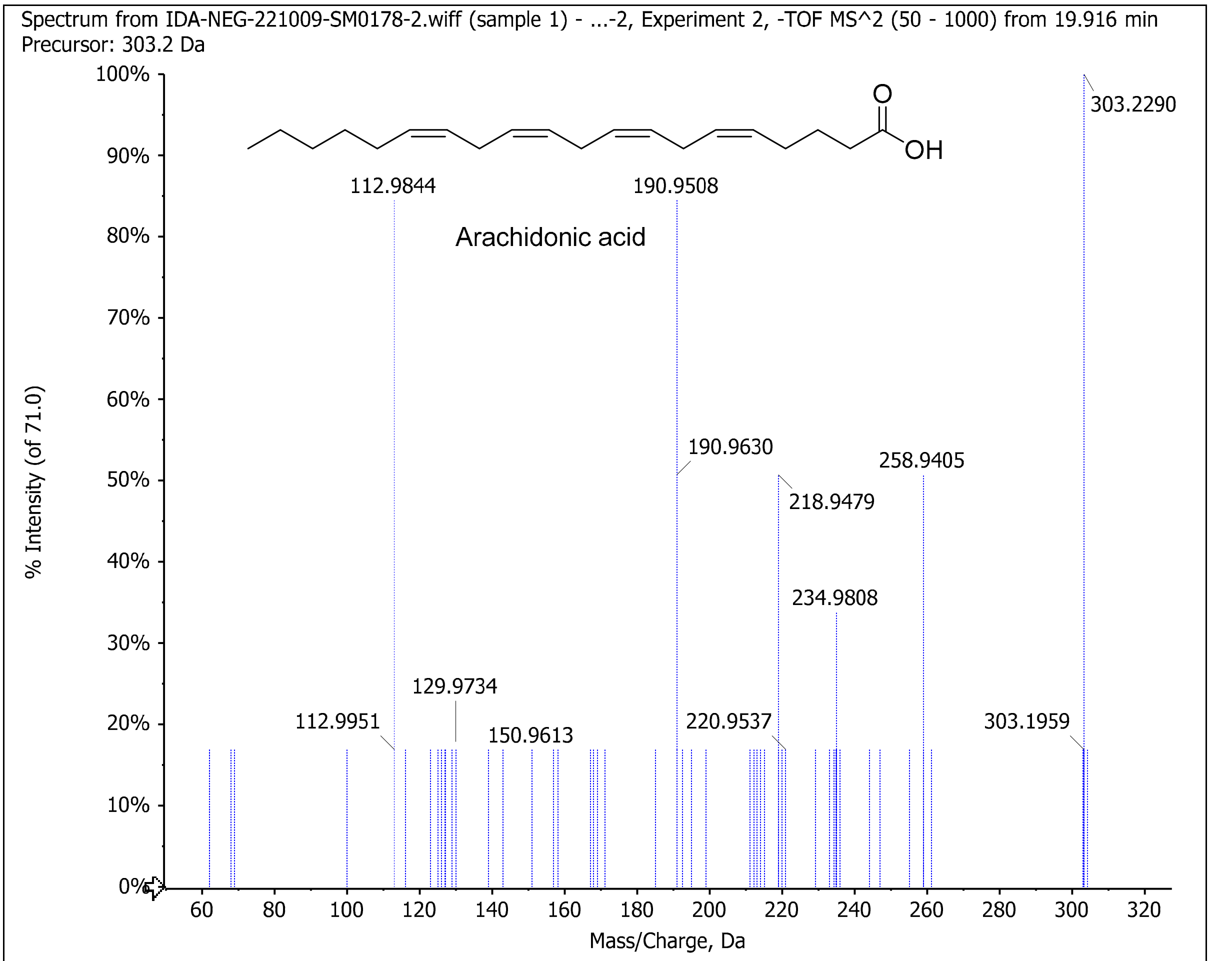

Spectrum from IDA-NEG-221009-SM0178-2.wiff (sample 1) - ...-2, Experiment 2, -TOF MS^2 (50 - 1000) from 19.916 min
Precursor: 303.2 Da
Arachidonic acid
O
OH
303.2290
112.9844
190.9508
190.9630
258.9405
218.9479
234.9808
129.9734
112.9951
150.9613
220.9537
303.1959
% Intensity (of 71.0)
Mass/Charge, Da

Figure S7

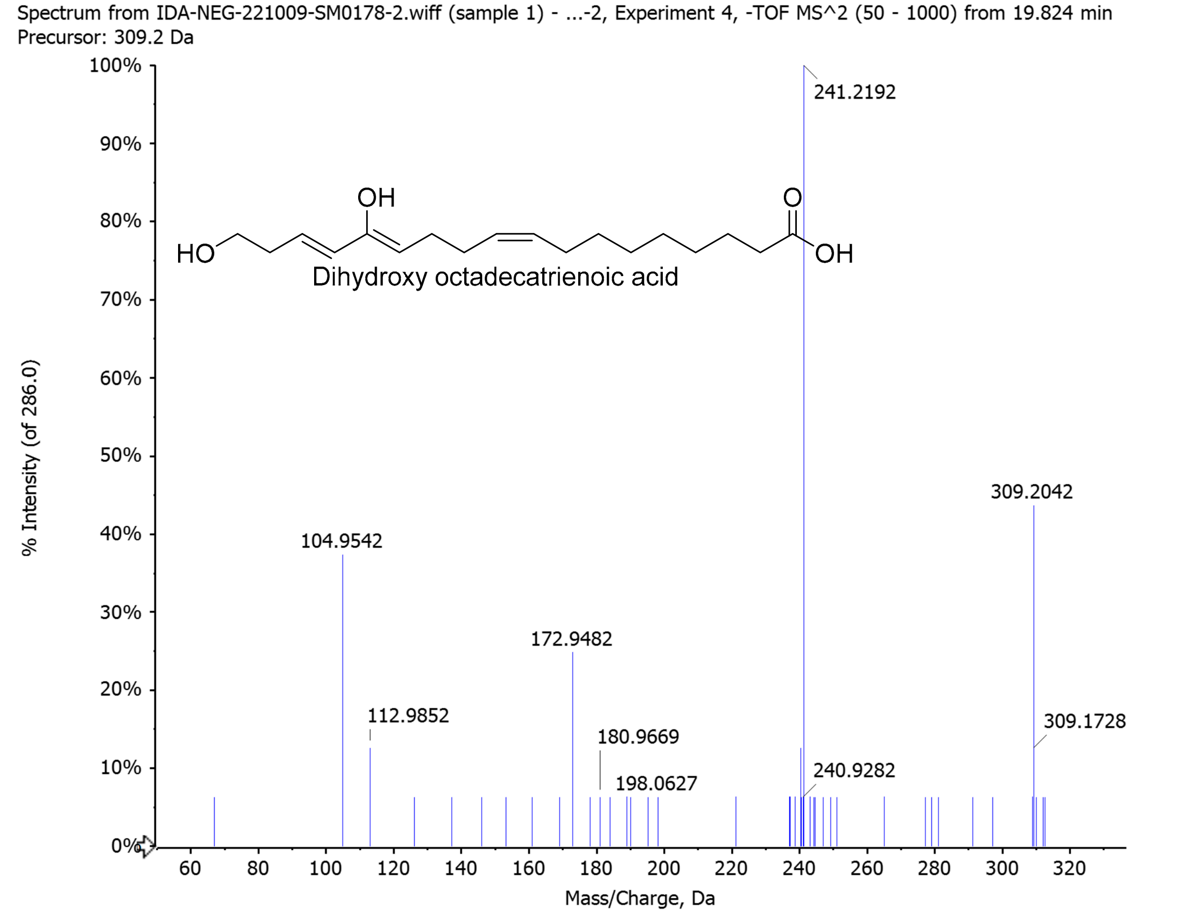
Spectrum from IDA-NEG-221009-SM0178-2.wiff (sample 1) - ...-2, Experiment 4, -TOF MS^2 (50 - 1000) from 19.824 min
Precursor: 309.2 Da
OH
O
HO
OH
Dihydroxy octadecatrienoic acid
241.2192
309.2042
104.9542
172.9482
112.9852
180.9669
198.0627
240.9282
309.1728
% Intensity (of 286.0)
Mass/Charge, Da

Figure S8

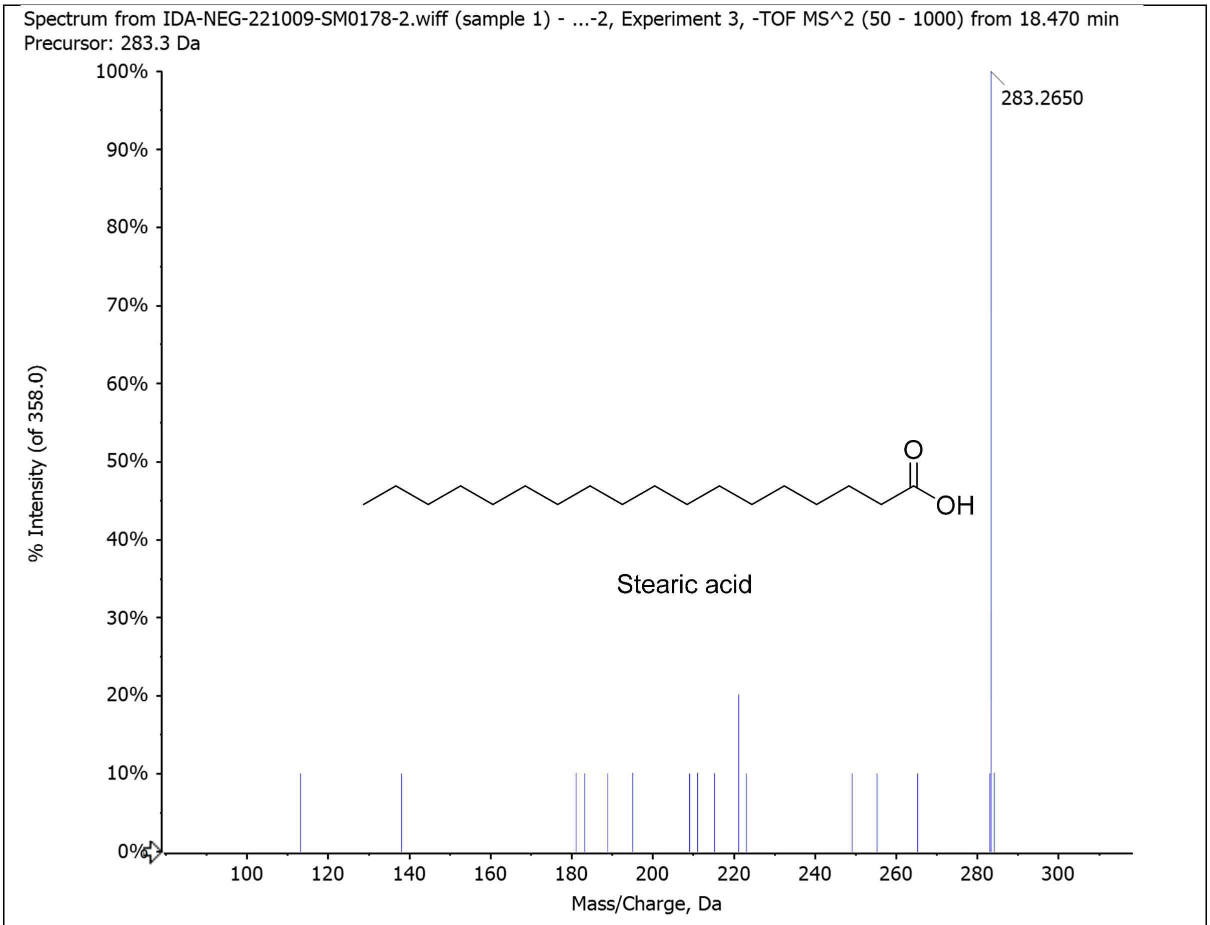
Spectrum from IDA-NEG-221009-SM0178-2.wiff (sample 1) - ...-2, Experiment 3, -TOF MS^2 (50 - 1000) from 18.470 min
Precursor: 283.3 Da
283.2650
% Intensity (of 358.0)
Mass/Charge, Da
O
OH
Stearic acid

Figure S9

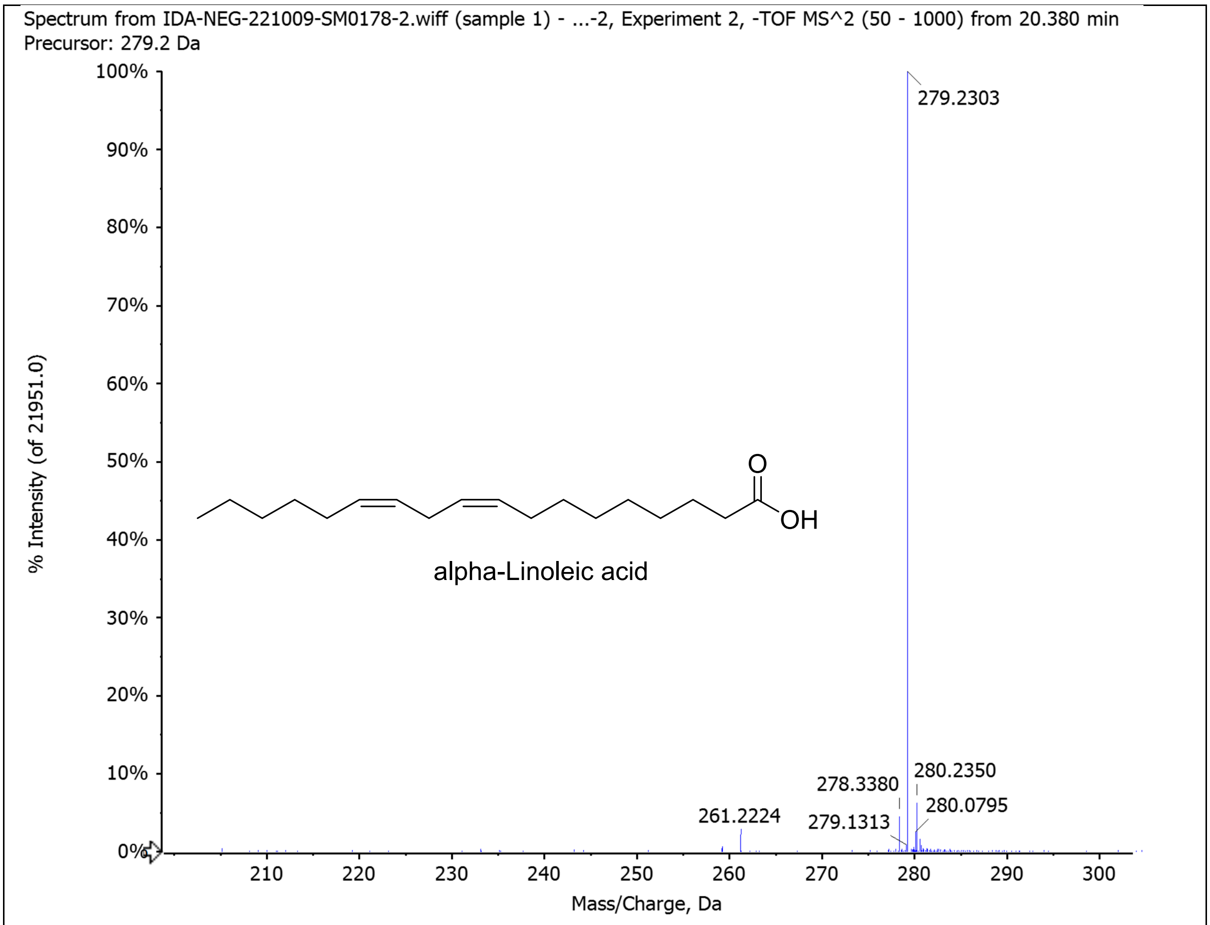
Spectrum from IDA-NEG-221009-SM0178-2.wiff (sample 1) - ...-2, Experiment 2, -TOF MS^2 (50 - 1000) from 20.380 min
Precursor: 279.2 Da
279.2303
280.2350
278.3380
280.0795
261.2224
279.1313
O
OH
alpha-Linoleic acid
% Intensity (of 21951.0)
Mass/Charge, Da
100%
90%
80%
70%
60%
50%
40%
30%
20%
10%
0%
210
220
230
240
250
260
270
280
290
300

Figure S10

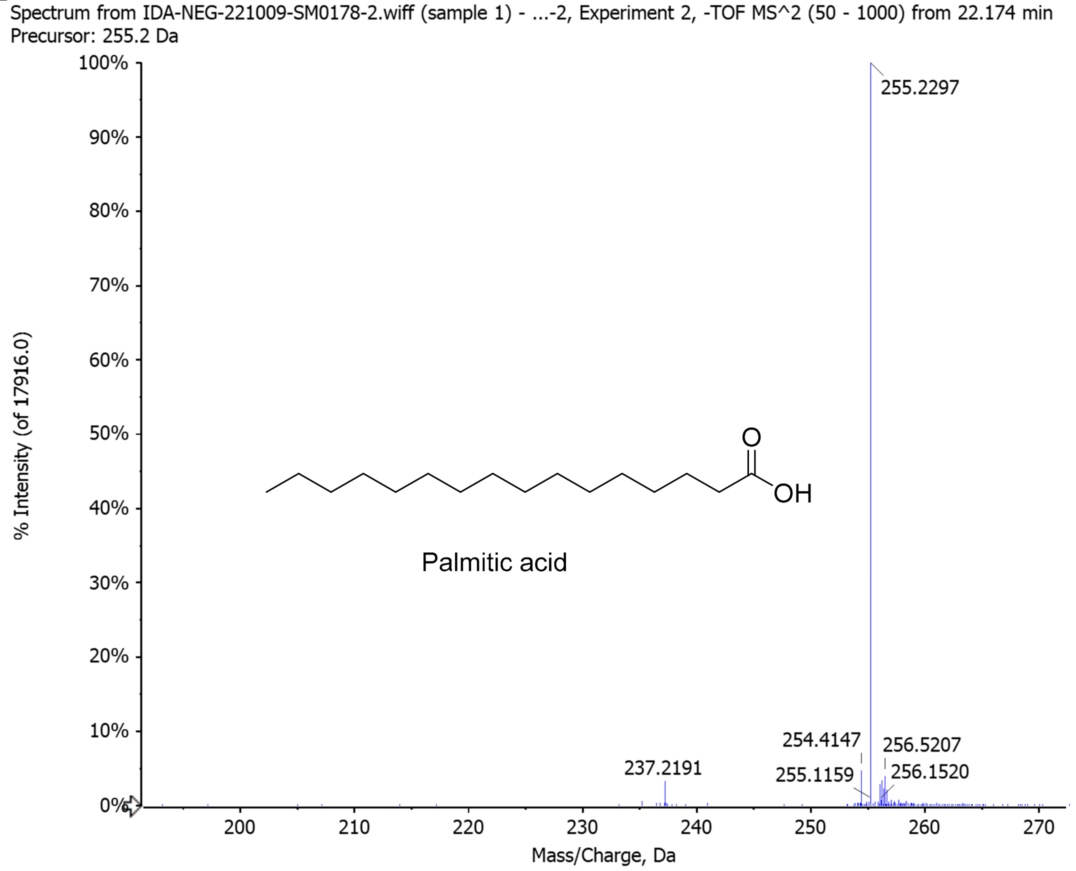
Spectrum from IDA-NEG-221009-SM0178-2.wiff (sample 1) - ...-2, Experiment 2, -TOF MS^2 (50 - 1000) from 22.174 min
Precursor: 255.2 Da
100%
90%
80%
70%
60%
50%
40%
30%
20%
10%
0%
% Intensity (of 17916.0)
255.2297
237.2191
254.4147
255.1159
256.5207
256.1520
O
OH
Palmitic acid
200
210
220
230
240
250
260
270
Mass/Charge, Da

Figure S11

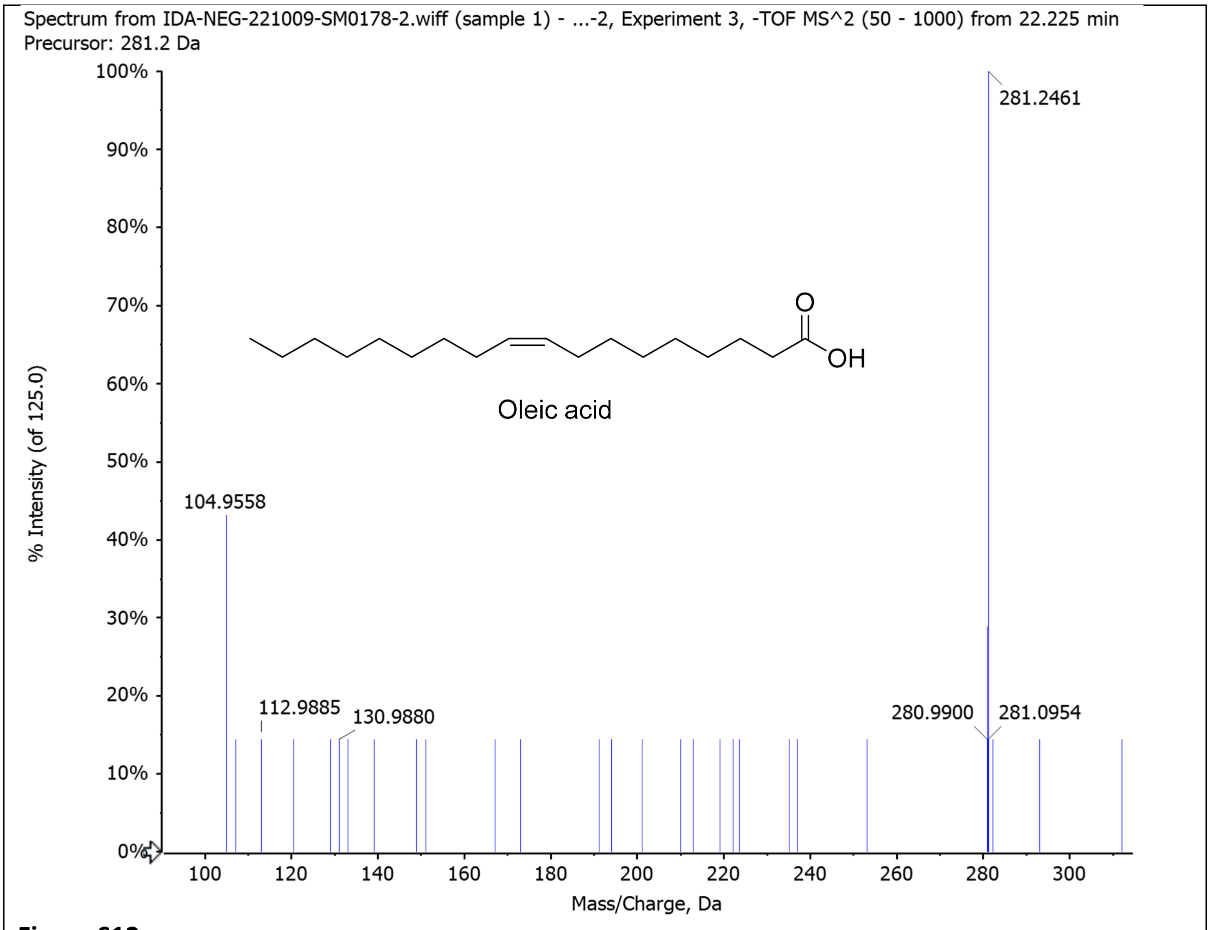

Spectrum from IDA-NEG-221009-SM0178-2.wiff (sample 1) - ...-2, Experiment 3, -TOF MS^2 (50 - 1000) from 22.225 min
Precursor: 281.2 Da
100%
90%
80%
70%
60%
50%
40%
30%
20%
10%
0%
% Intensity (of 125.0)
O
OH
Oleic acid
281.2461
104.9558
112.9885
130.9880
280.9900
281.0954
100
120
140
160
180
200
220
240
260
280
300
Mass/Charge, Da

Figure S12

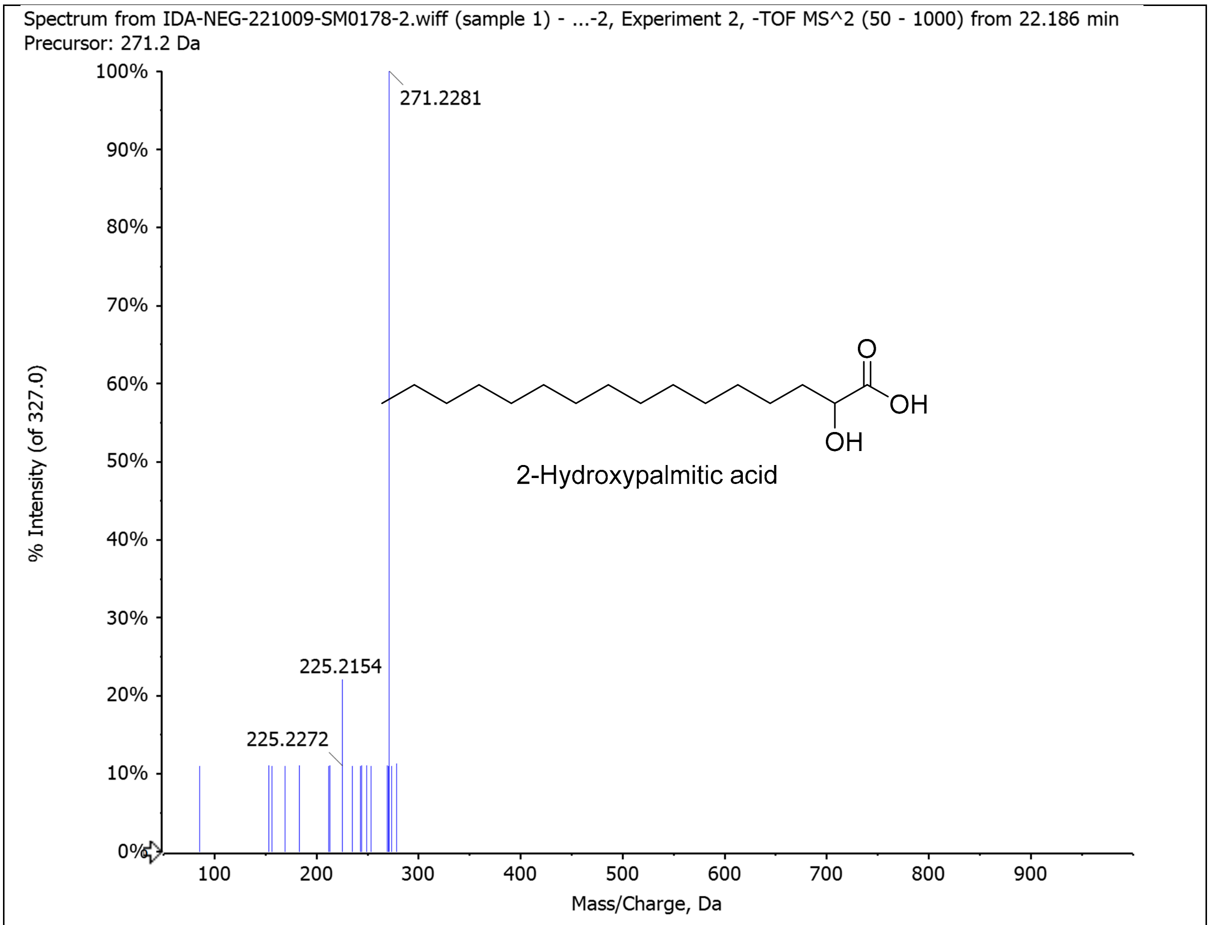

Spectrum from IDA-NEG-221009-SM0178-2.wiff (sample 1) - ...-2, Experiment 2, -TOF MS^2 (50 - 1000) from 22.186 min
Precursor: 271.2 Da
271.2281
225.2154
225.2272
% Intensity (of 327.0)
Mass/Charge, Da
O
OH
OH
2-Hydroxypalmitic acid

Figure S13

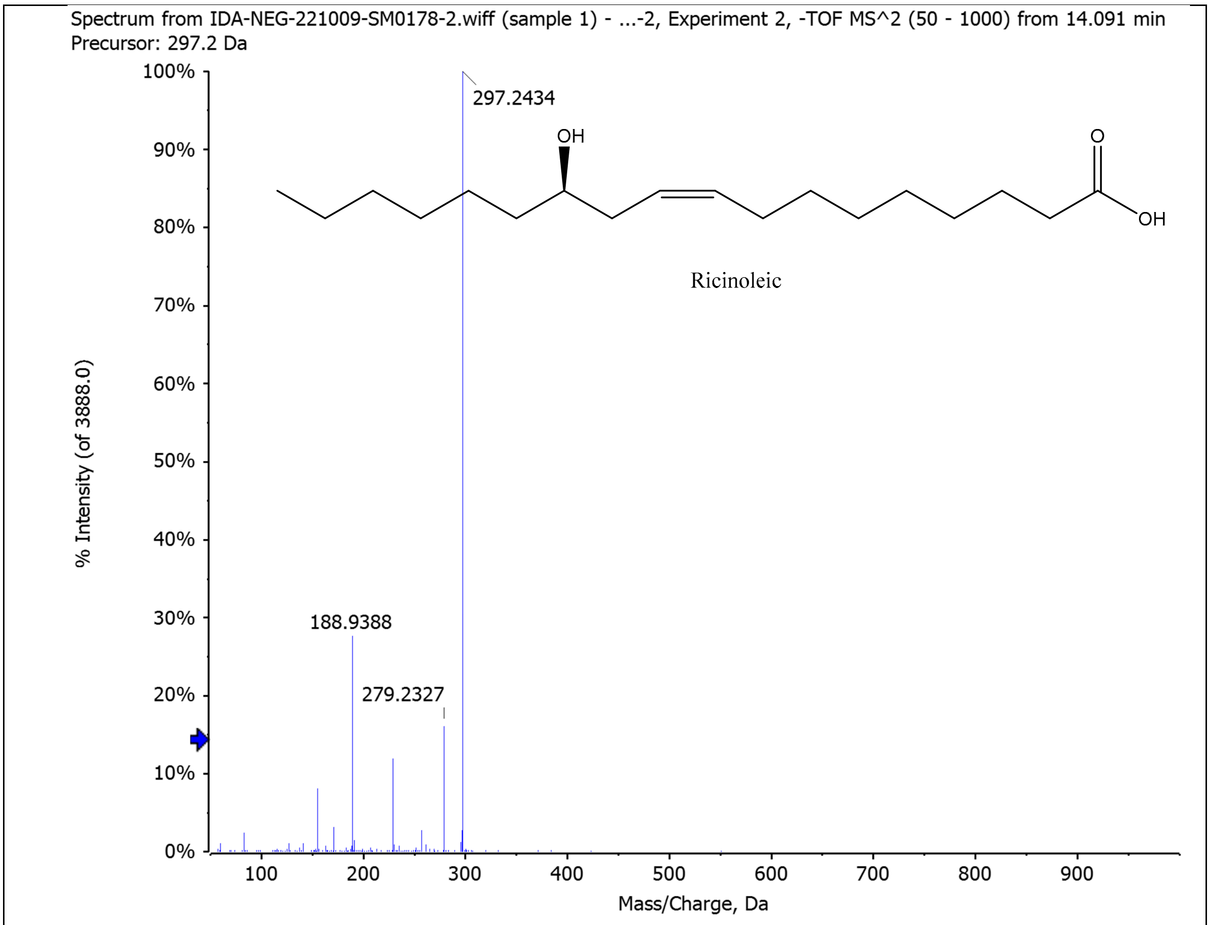
Spectrum from IDA-NEG-221009-SM0178-2.wiff (sample 1) - ...-2, Experiment 2, -TOF MS^2 (50 - 1000) from 14.091 min
Precursor: 297.2 Da
297.2434
188.9388
279.2327
OH
O
OH
Ricinoleic
% Intensity (of 3888.0)
100%
90%
80%
70%
60%
50%
40%
30%
20%
10%
0%
100
200
300
400
500
600
700
800
900
Mass/Charge, Da

Figure S14

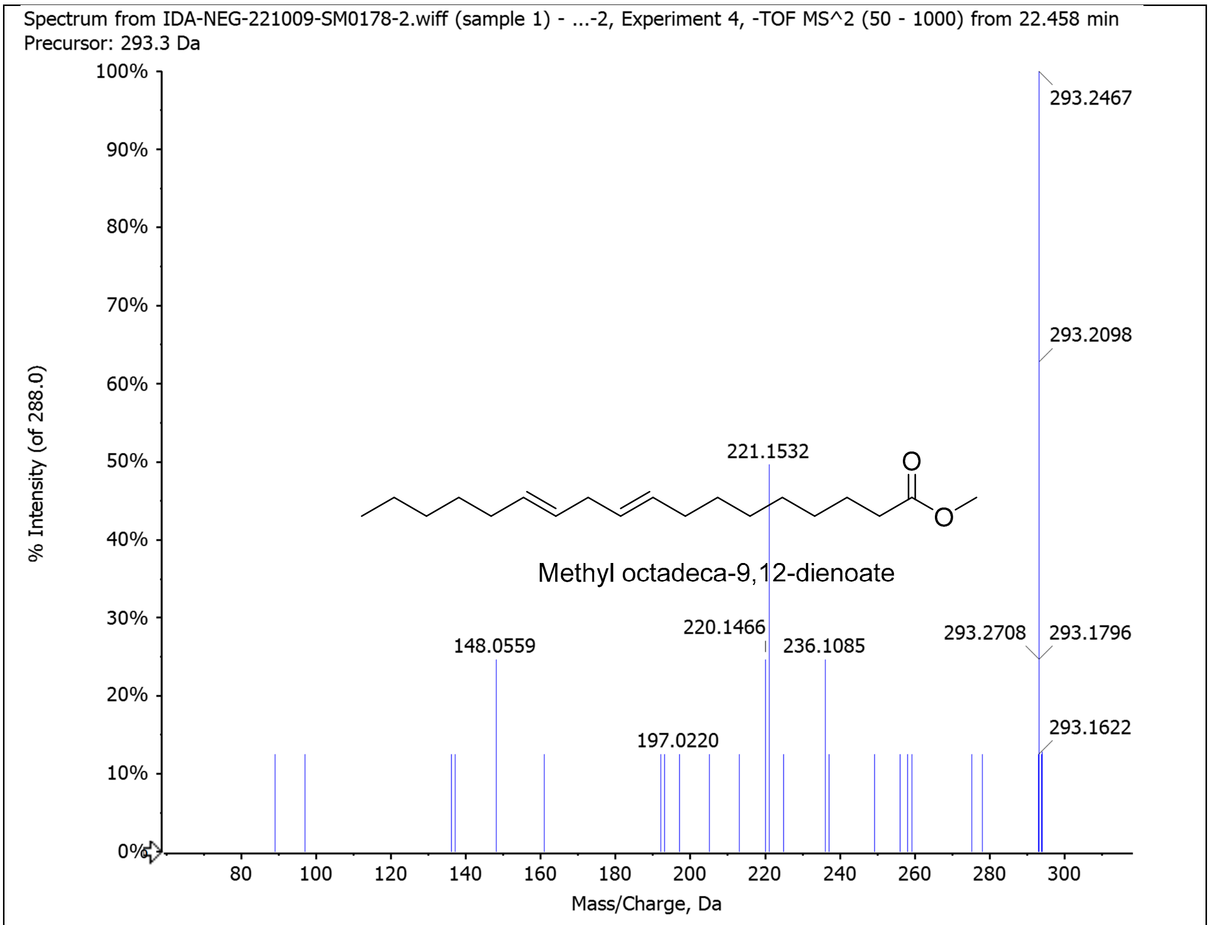

Spectrum from IDA-NEG-221009-SM0178-2.wiff (sample 1) - ...-2, Experiment 4, -TOF MS^2 (50 - 1000) from 22.458 min
Precursor: 293.3 Da
100%
90%
80%
70%
60%
50%
40%
30%
20%
10%
0%
% Intensity (of 288.0)
293.2467
293.2098
221.1532
Methyl octadeca-9,12-dienoate
220.1466
148.0559
236.1085
293.2708
293.1796
197.0220
293.1622
80
100
120
140
160
180
200
220
240
260
280
300
Mass/Charge, Da

Figure S15

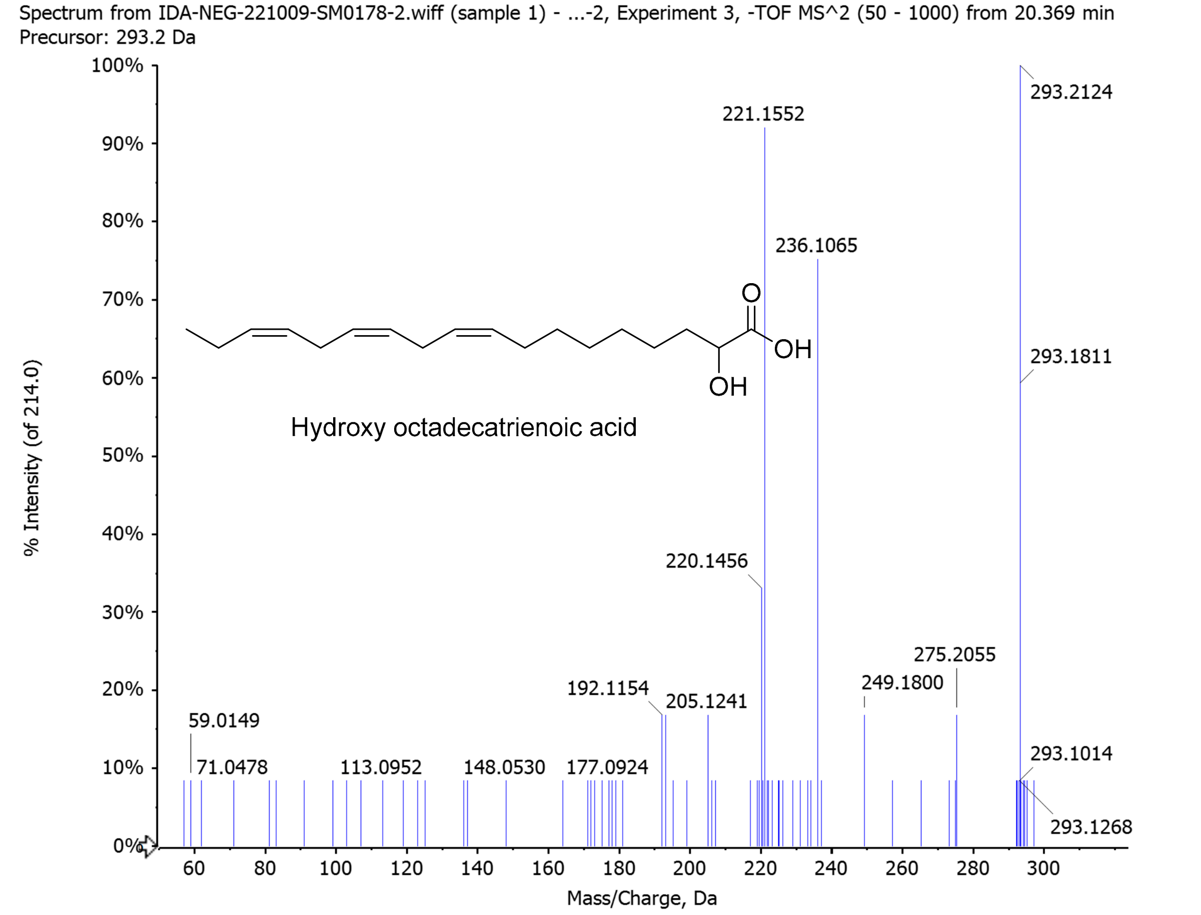
Spectrum from IDA-NEG-221009-SM0178-2.wiff (sample 1) - ...-2, Experiment 3, -TOF MS^2 (50 - 1000) from 20.369 min
Precursor: 293.2 Da
% Intensity (of 214.0)
Mass/Charge, Da
Hydroxy octadecatrienoic acid
O
OH
OH
293.2124
221.1552
236.1065
293.1811
220.1456
275.2055
249.1800
192.1154
205.1241
59.0149
71.0478
113.0952
148.0530
177.0924
293.1014
293.1268

Figure S16

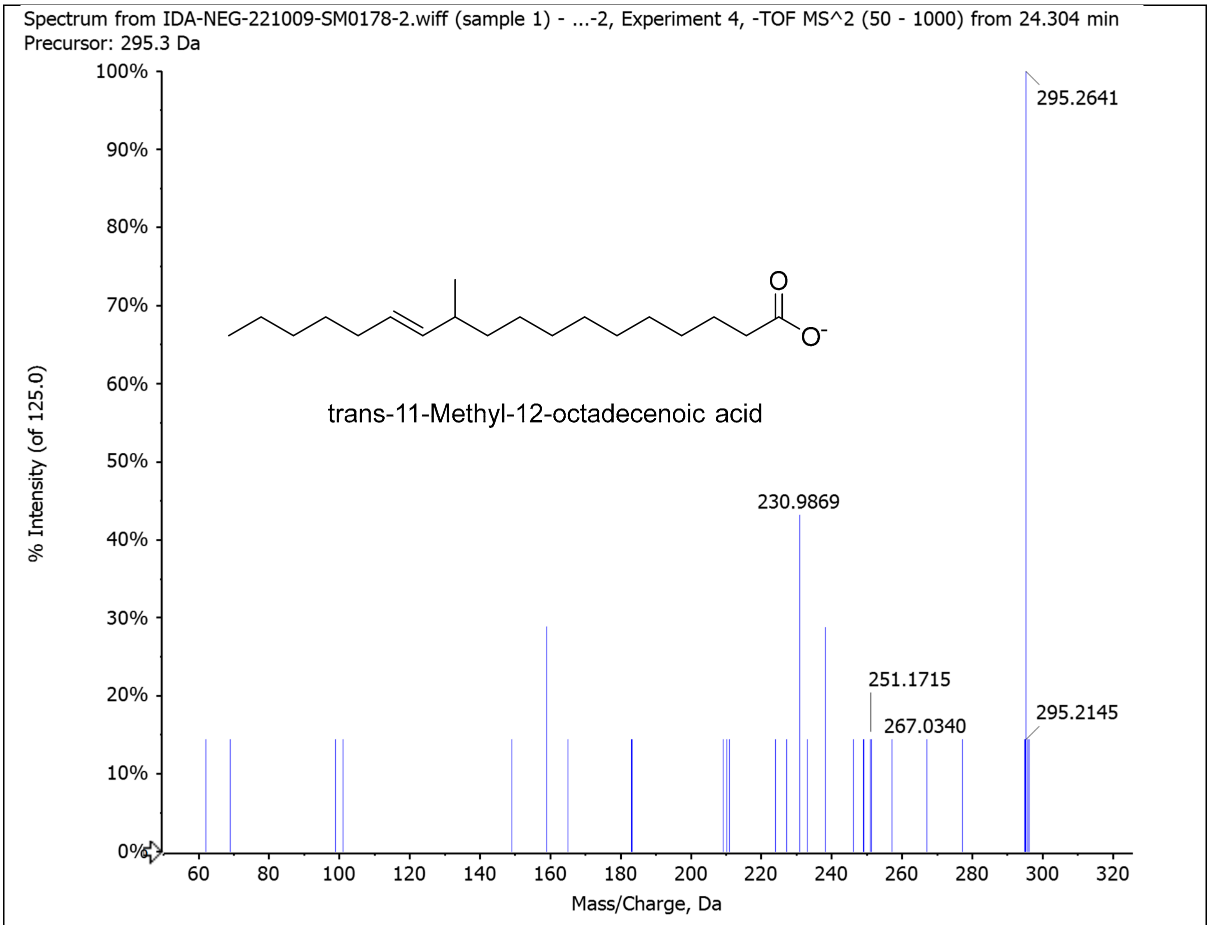
Spectrum from IDA-NEG-221009-SM0178-2.wiff (sample 1) - ...-2, Experiment 4, -TOF MS^2 (50 - 1000) from 24.304 min
Precursor: 295.3 Da
trans-11-Methyl-12-octadecenoic acid
295.2641
230.9869
251.1715
267.0340
295.2145
% Intensity (of 125.0)
Mass/Charge, Da

Figure S17

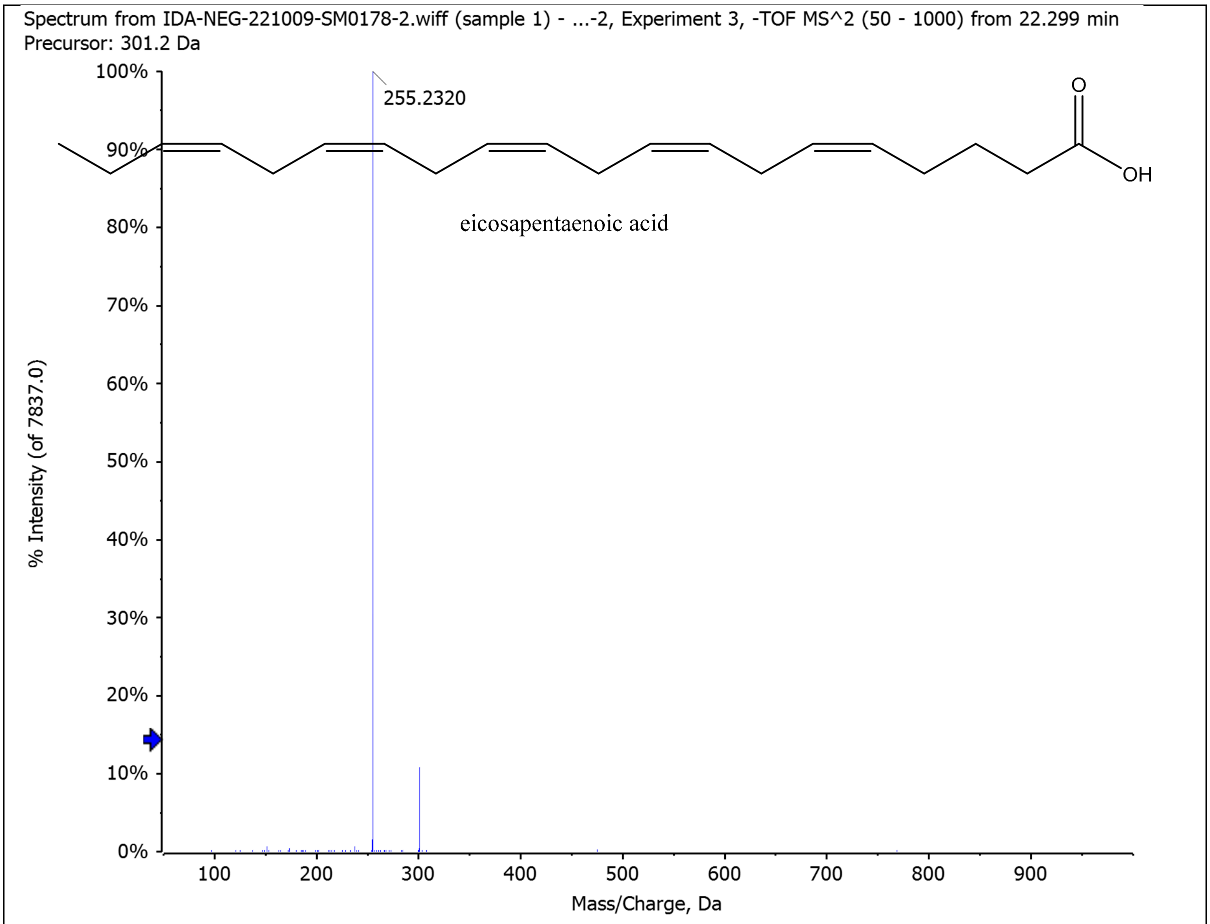

Spectrum from IDA-NEG-221009-SM0178-2.wiff (sample 1) - ...-2, Experiment 3, -TOF MS^2 (50 - 1000) from 22.299 min
Precursor: 301.2 Da
255.2320
eicosapentaenoic acid
O
OH
% Intensity (of 7837.0)
100%
90%
80%
70%
60%
50%
40%
30%
20%
10%
0%
100
200
300
400
500
600
700
800
900
Mass/Charge, Da

Figure S18

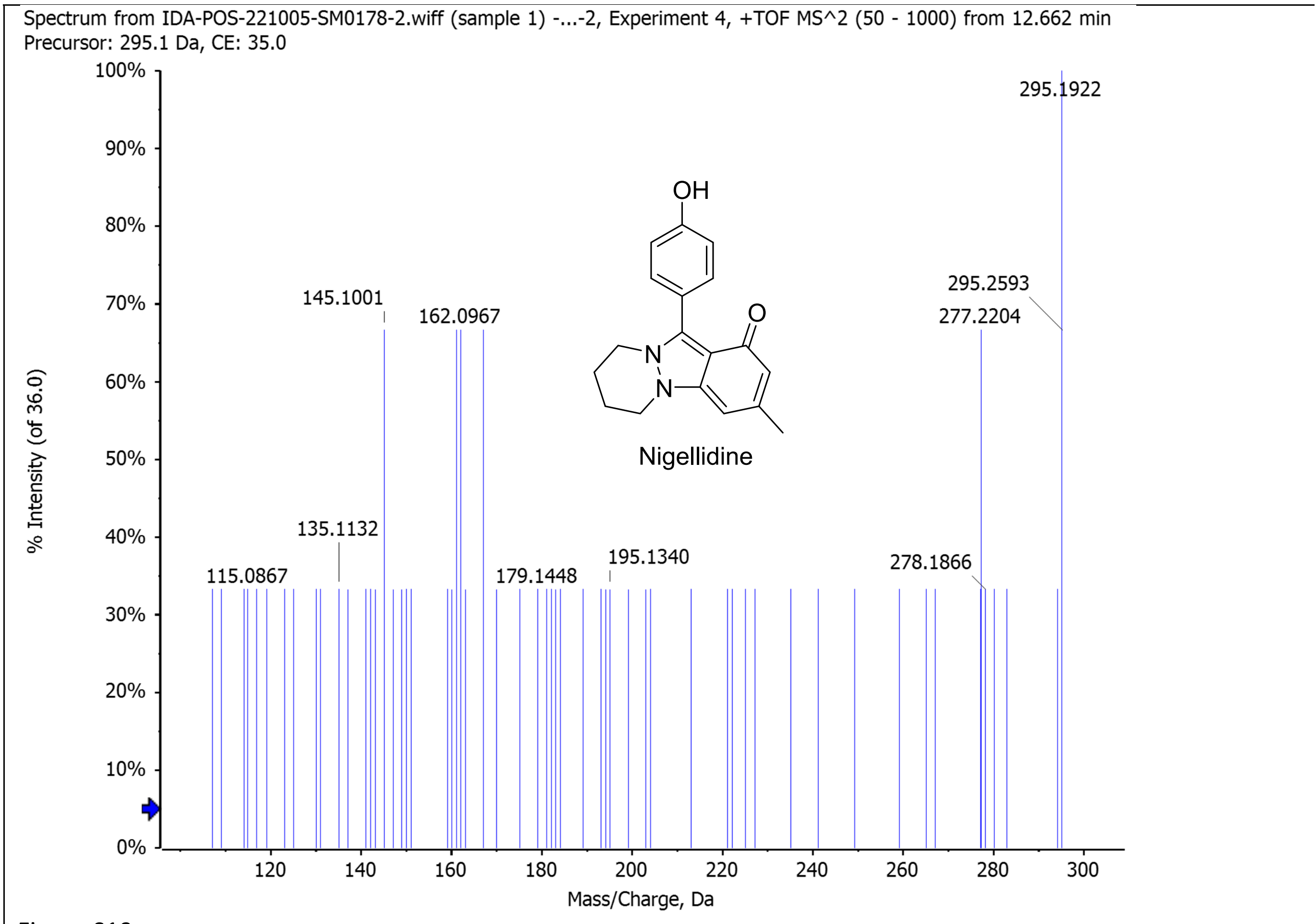

Spectrum from IDA-POS-221005-SM0178-2.wiff (sample 1) -...-2, Experiment 4, +TOF MS^2 (50 - 1000) from 12.662 min
Precursor: 295.1 Da, CE: 35.0
% Intensity (of 36.0)
Mass/Charge, Da
295.1922
295.2593
277.2204
145.1001
162.0967
135.1132
115.0867
179.1448
195.1340
278.1866
OH
O
N
N
Nigellidine

Figure S19

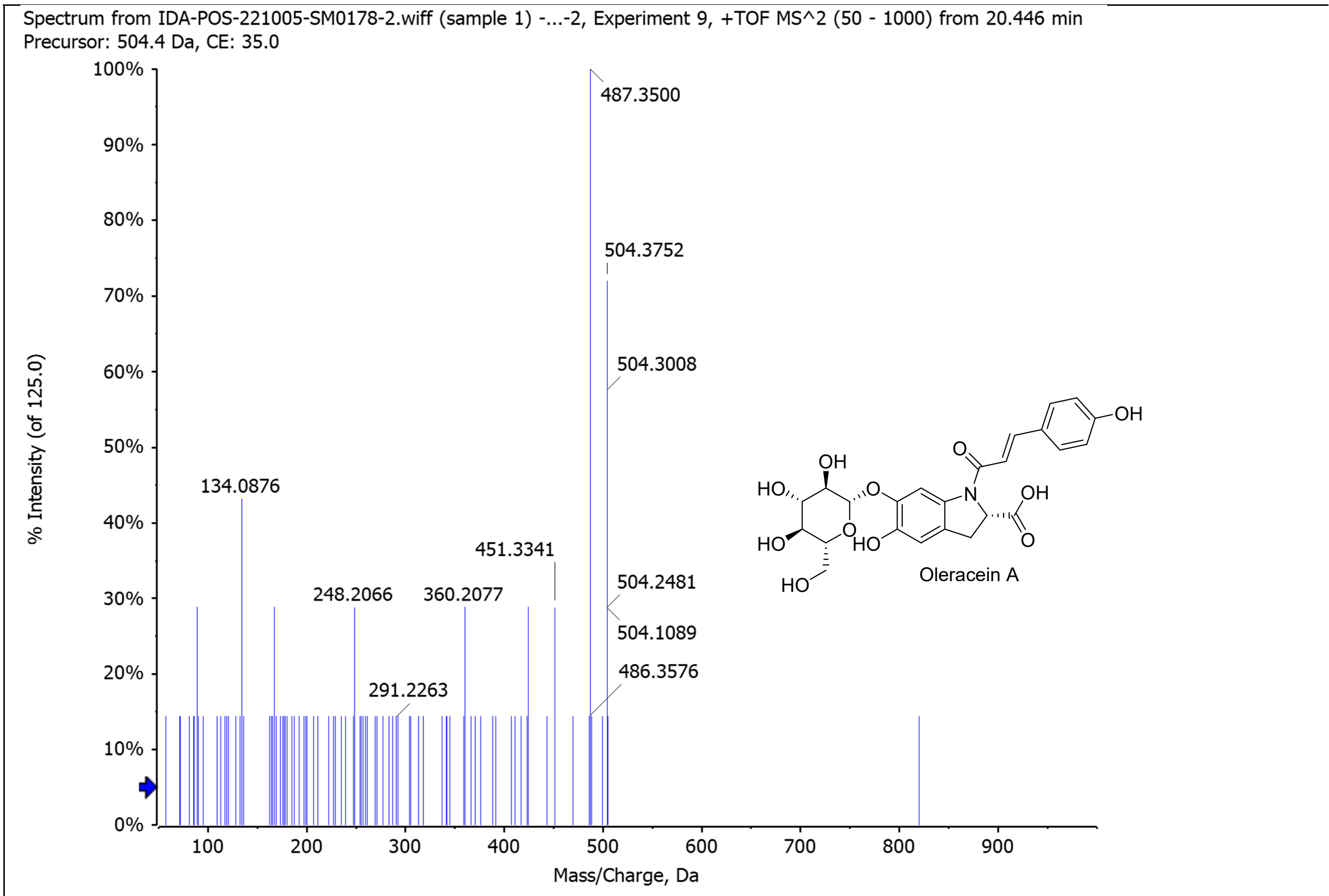
Spectrum from IDA-POS-221005-SM0178-2.wiff (sample 1) -...-2, Experiment 9, +TOF MS^2 (50 - 1000) from 20.446 min
Precursor: 504.4 Da, CE: 35.0
% Intensity (of 125.0)
Mass/Charge, Da
487.3500
504.3752
504.3008
134.0876
451.3341
248.2066
360.2077
504.2481
504.1089
486.3576
291.2263
Oleracein A

Figure S20

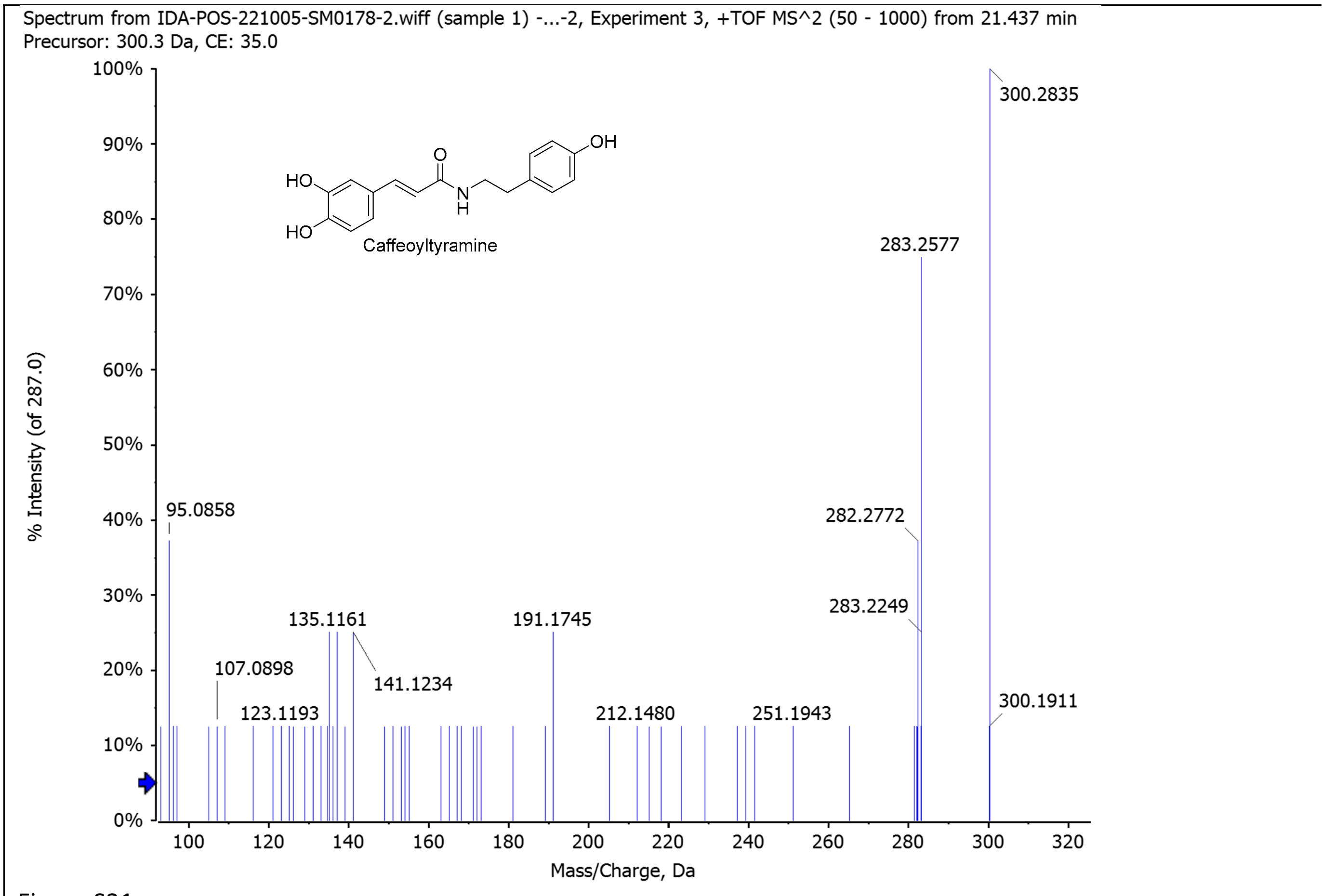
Spectrum from IDA-POS-221005-SM0178-2.wiff (sample 1) -...-2, Experiment 3, +TOF MS^2 (50 - 1000) from 21.437 min
Precursor: 300.3 Da, CE: 35.0
Caffeoyltyramine
300.2835
283.2577
282.2772
283.2249
300.1911
95.0858
107.0898
123.1193
135.1161
141.1234
191.1745
212.1480
251.1943
% Intensity (of 287.0)
Mass/Charge, Da

Figure S21

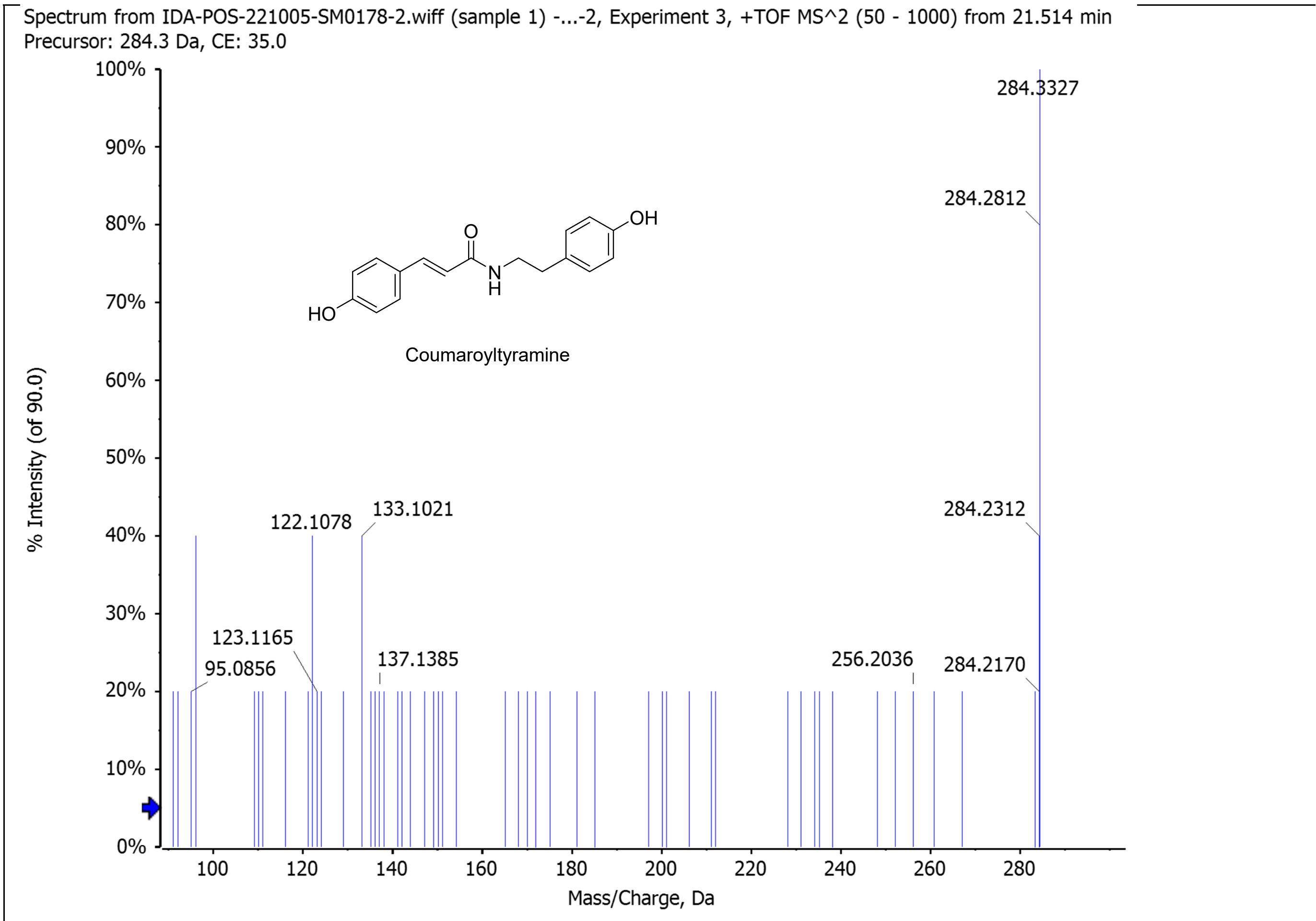
Spectrum from IDA-POS-221005-SM0178-2.wiff (sample 1) -...-2, Experiment 3, +TOF MS^2 (50 - 1000) from 21.514 min
Precursor: 284.3 Da, CE: 35.0
284.3327
284.2812
284.2312
284.2170
256.2036
122.1078
133.1021
123.1165
95.0856
137.1385
Coumaroyltyramine
% Intensity (of 90.0)
Mass/Charge, Da

Figure S22

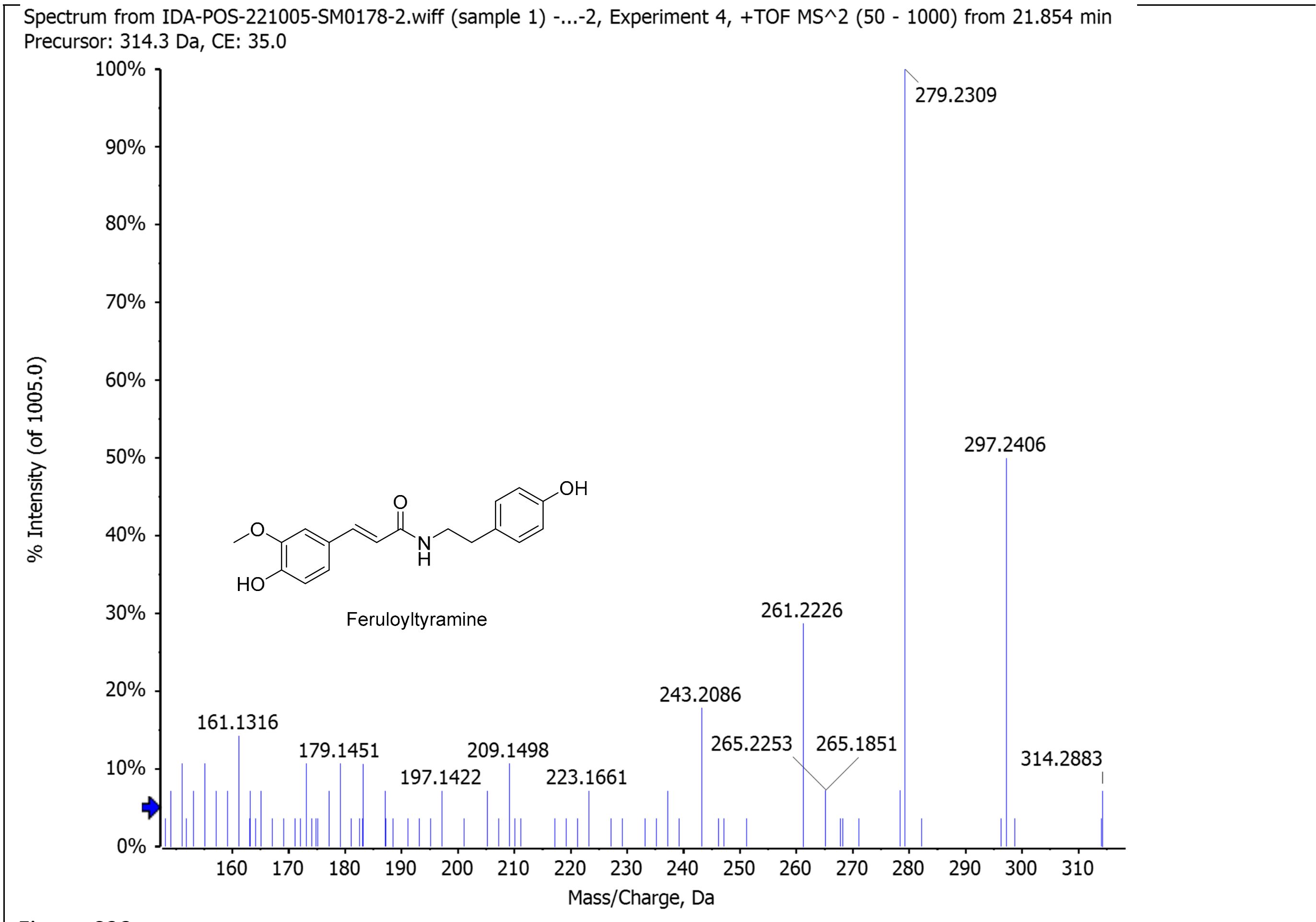

Spectrum from IDA-POS-221005-SM0178-2.wiff (sample 1) -...-2, Experiment 4, +TOF MS^2 (50 - 1000) from 21.854 min
Precursor: 314.3 Da, CE: 35.0
% Intensity (of 1005.0)
Mass/Charge, Da
279.2309
297.2406
261.2226
243.2086
161.1316
179.1451
209.1498
197.1422
223.1661
265.2253
265.1851
314.2883
Feruloyltyramine

Figure S23

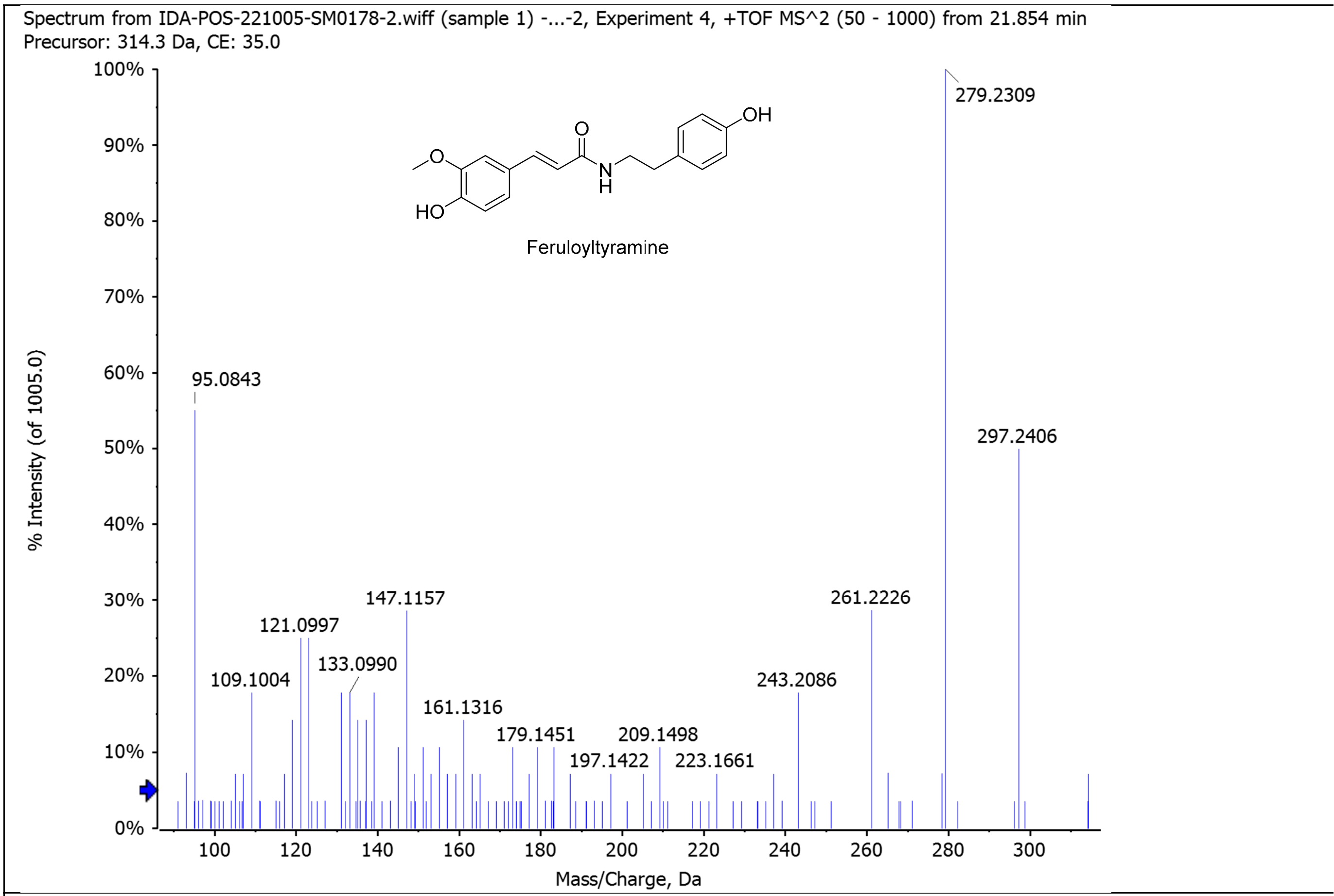

Spectrum from IDA-POS-221005-SM0178-2.wiff (sample 1) -...-2, Experiment 4, +TOF MS^2 (50 - 1000) from 21.854 min
Precursor: 314.3 Da, CE: 35.0
Feruloyltyramine
OH
O
N
H
HO
279.2309
95.0843
297.2406
147.1157
261.2226
121.0997
133.0990
109.1004
243.2086
161.1316
179.1451
209.1498
197.1422
223.1661
% Intensity (of 1005.0)
Mass/Charge, Da

Figure S24

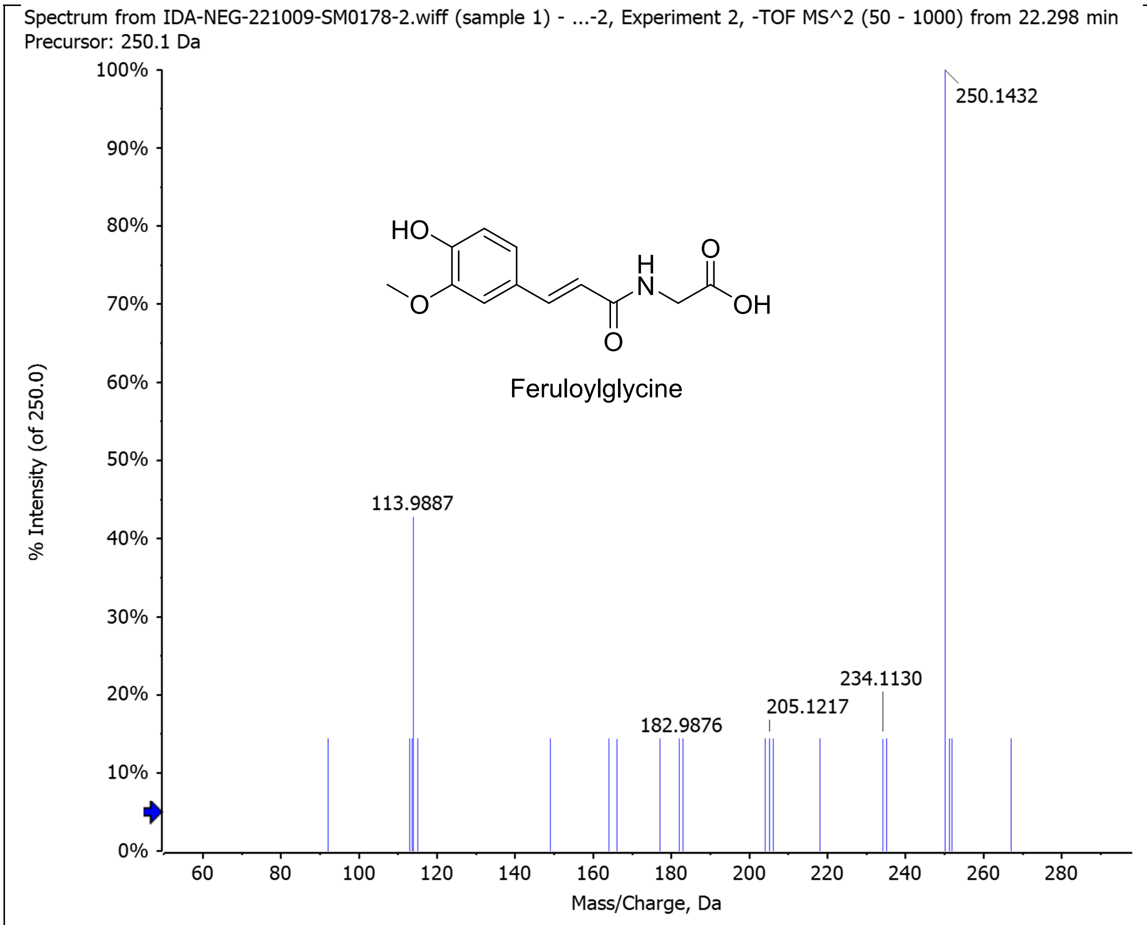

Spectrum from IDA-NEG-221009-SM0178-2.wiff (sample 1) - ...-2, Experiment 2, -TOF MS^2 (50 - 1000) from 22.298 min
Precursor: 250.1 Da
% Intensity (of 250.0)
Mass/Charge, Da
250.1432
113.9887
234.1130
205.1217
182.9876
HO
O
H
N
O
OH
O
Feruloylglycine

Figure S25

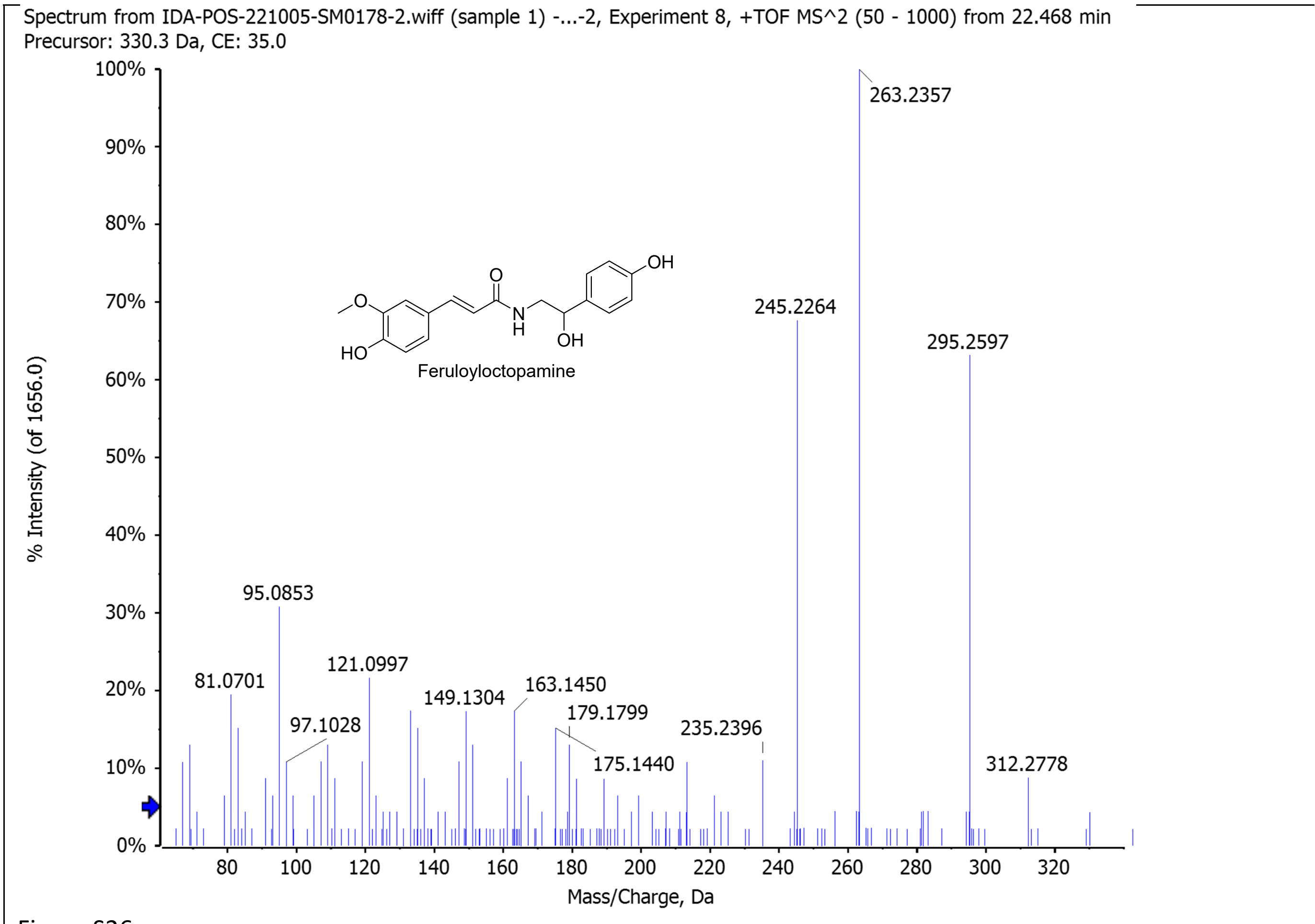

Spectrum from IDA-POS-221005-SM0178-2.wiff (sample 1) -...-2, Experiment 8, +TOF MS^2 (50 - 1000) from 22.468 min
Precursor: 330.3 Da, CE: 35.0
% Intensity (of 1656.0)
Mass/Charge, Da
Feruloyloctopamine
263.2357
245.2264
295.2597
95.0853
121.0997
81.0701
149.1304
163.1450
179.1799
97.1028
235.2396
175.1440
312.2778

Figure S26

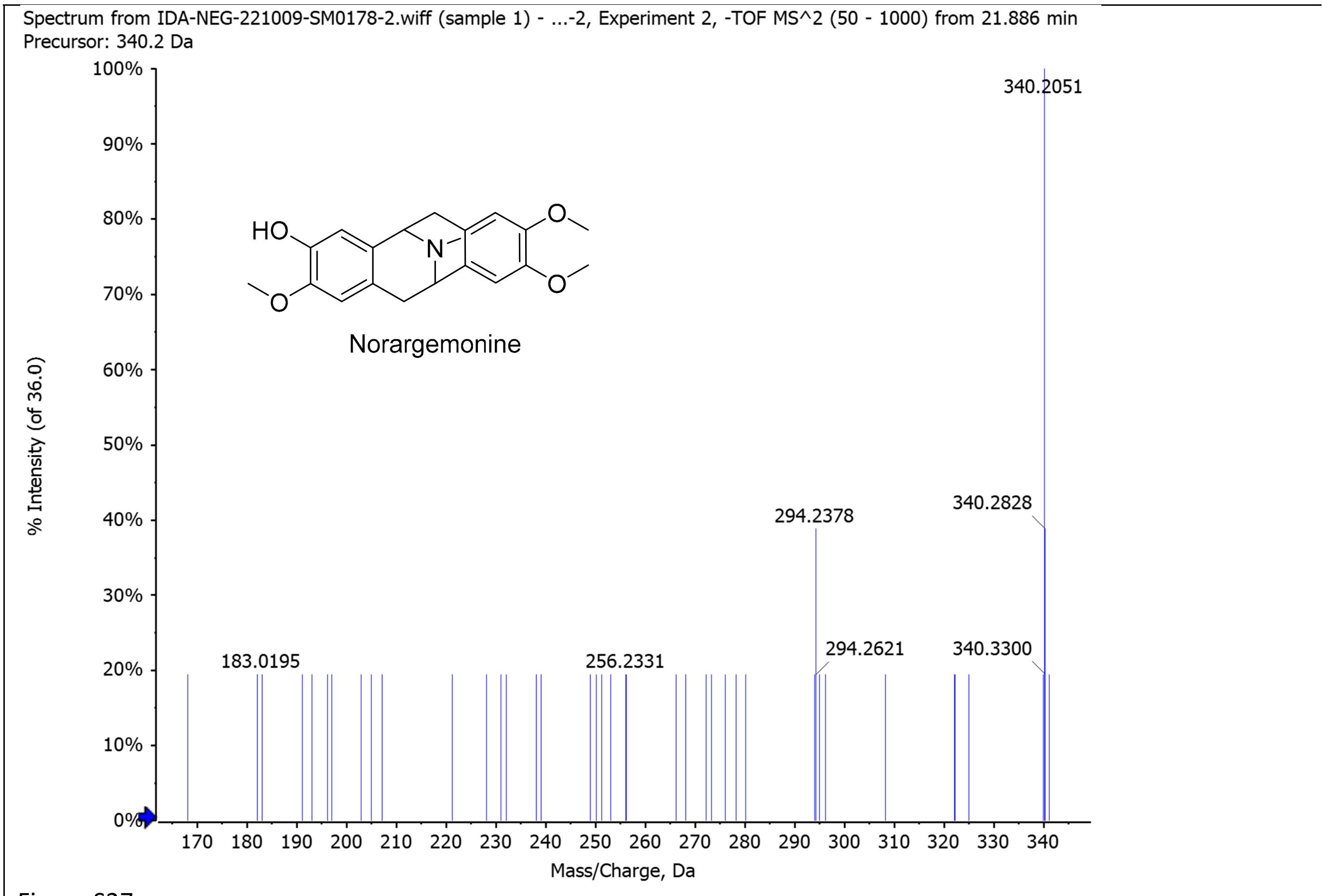
Spectrum from IDA-NEG-221009-SM0178-2.wiff (sample 1) - ...-2, Experiment 2, -TOF MS^2 (50 - 1000) from 21.886 min
Precursor: 340.2 Da
340.2051
294.2378
340.2828
183.0195
256.2331
294.2621
340.3300
HO
N
O
O
O
Norargemonine
% Intensity (of 36.0)
Mass/Charge, Da
100%
90%
80%
70%
60%
50%
40%
30%
20%
10%
0%
170 180 190 200 210 220 230 240 250 260 270 280 290 300 310 320 330 340

Figure S27

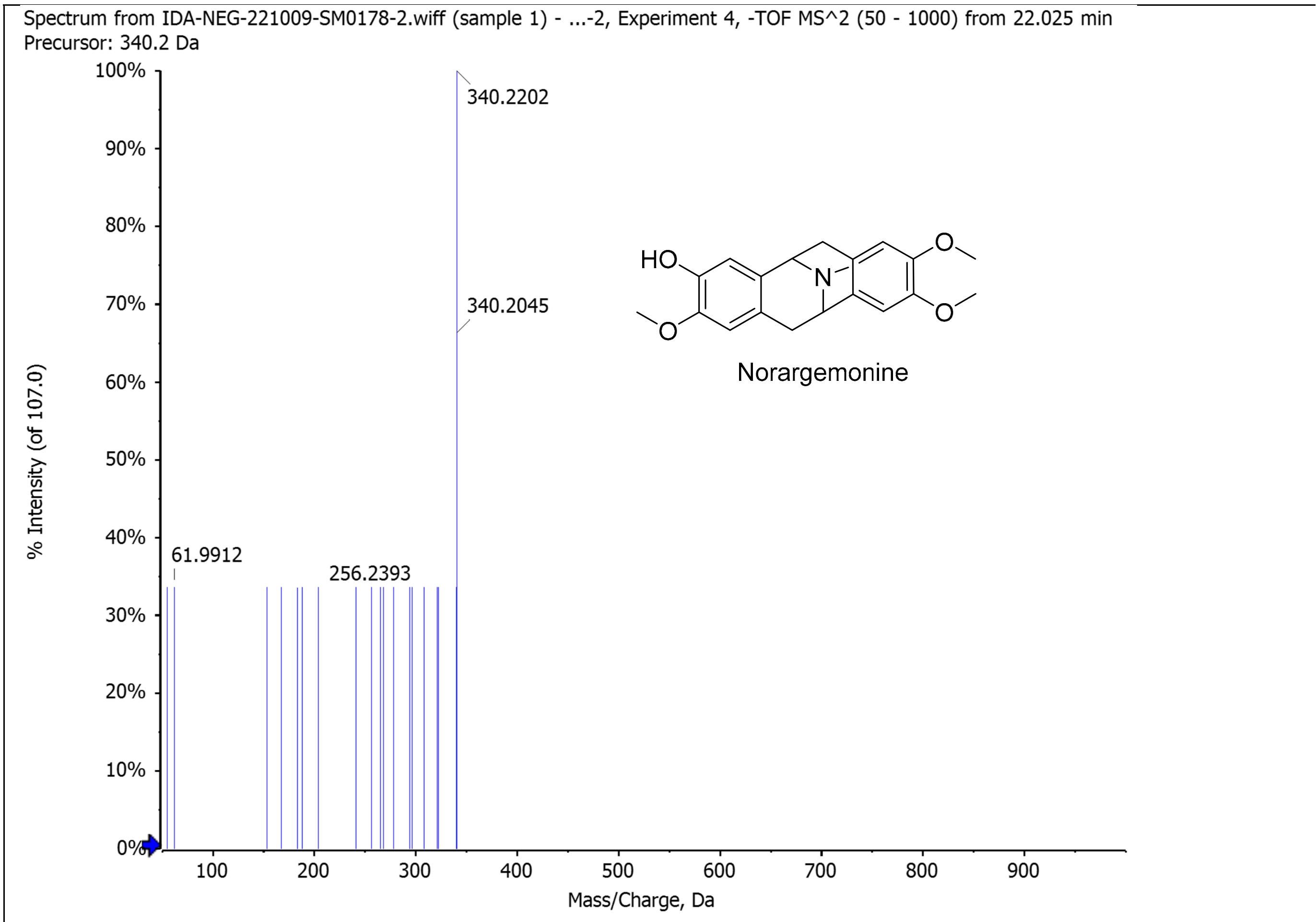

Spectrum from IDA-NEG-221009-SM0178-2.wiff (sample 1) - ...-2, Experiment 4, -TOF MS^2 (50 - 1000) from 22.025 min
Precursor: 340.2 Da
% Intensity (of 107.0)
100%
90%
80%
70%
60%
50%
40%
30%
20%
10%
0%
340.2202
340.2045
61.9912
256.2393
100
200
300
400
500
600
700
800
900
Mass/Charge, Da
HO
N
O
O
O
Norargemonine

Figure S28

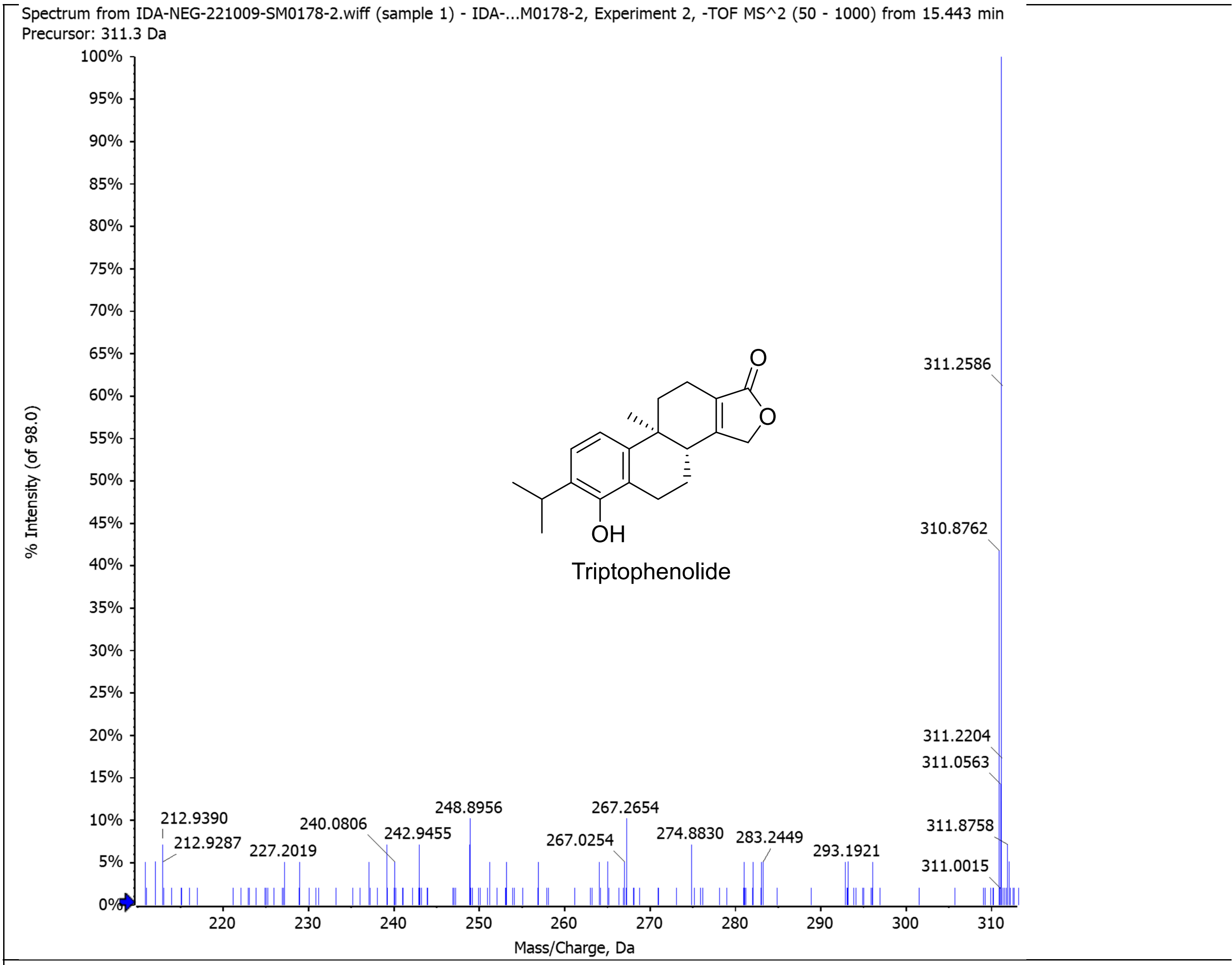

Spectrum from IDA-NEG-221009-SM0178-2.wiff (sample 1) - IDA-...M0178-2, Experiment 2, -TOF MS^2 (50 - 1000) from 15.443 min
Precursor: 311.3 Da
% Intensity (of 98.0)
Mass/Charge, Da
311.2586
310.8762
311.2204
311.0563
311.8758
311.0015
212.9390
212.9287
227.2019
240.0806
242.9455
248.8956
267.0254
267.2654
274.8830
283.2449
293.1921
OH
O
O
Triptophenolide

Figure S29.

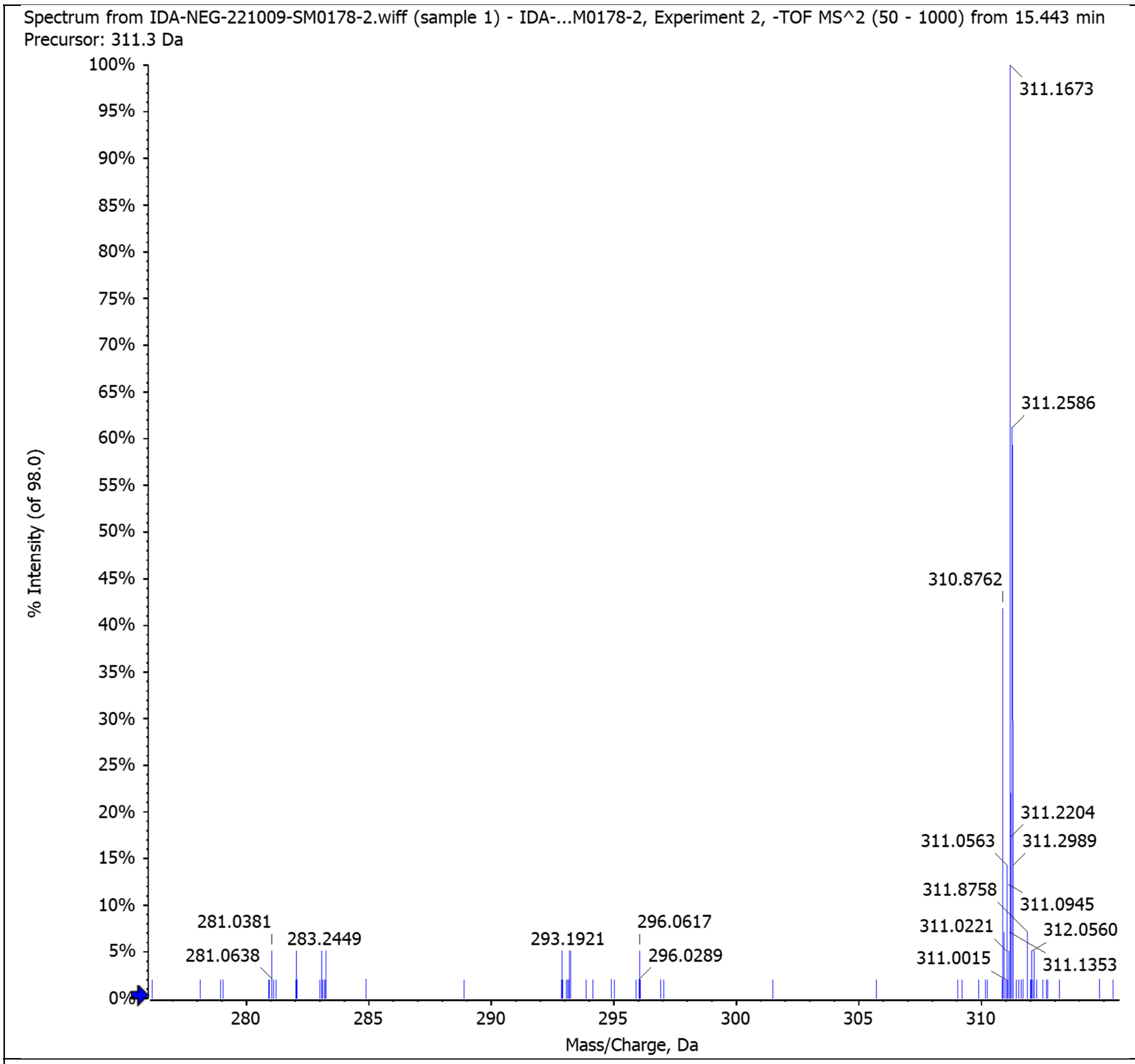

Figure S30. Triptophenolide

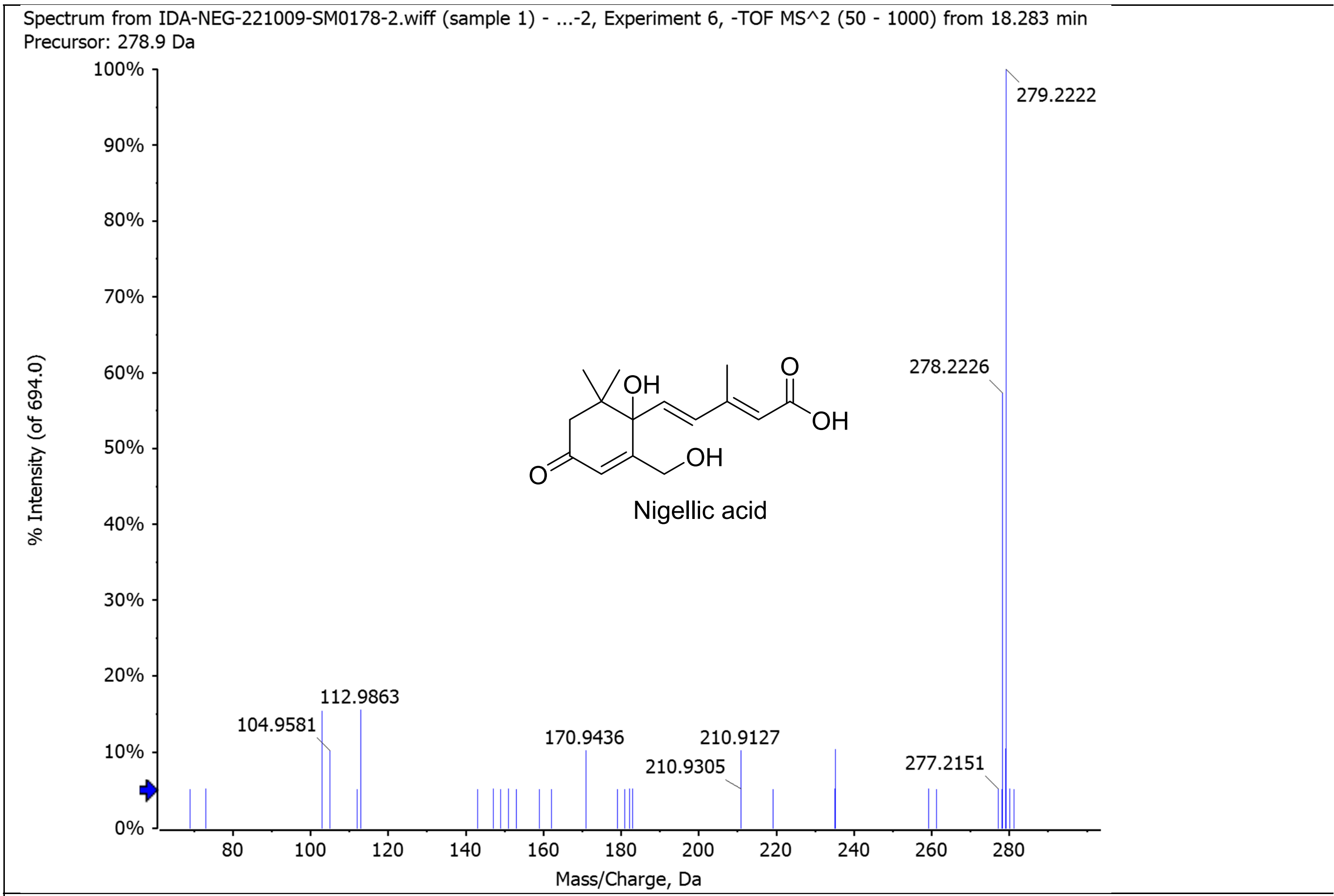

Figure S31. Nigellic acid, Monoterpenes

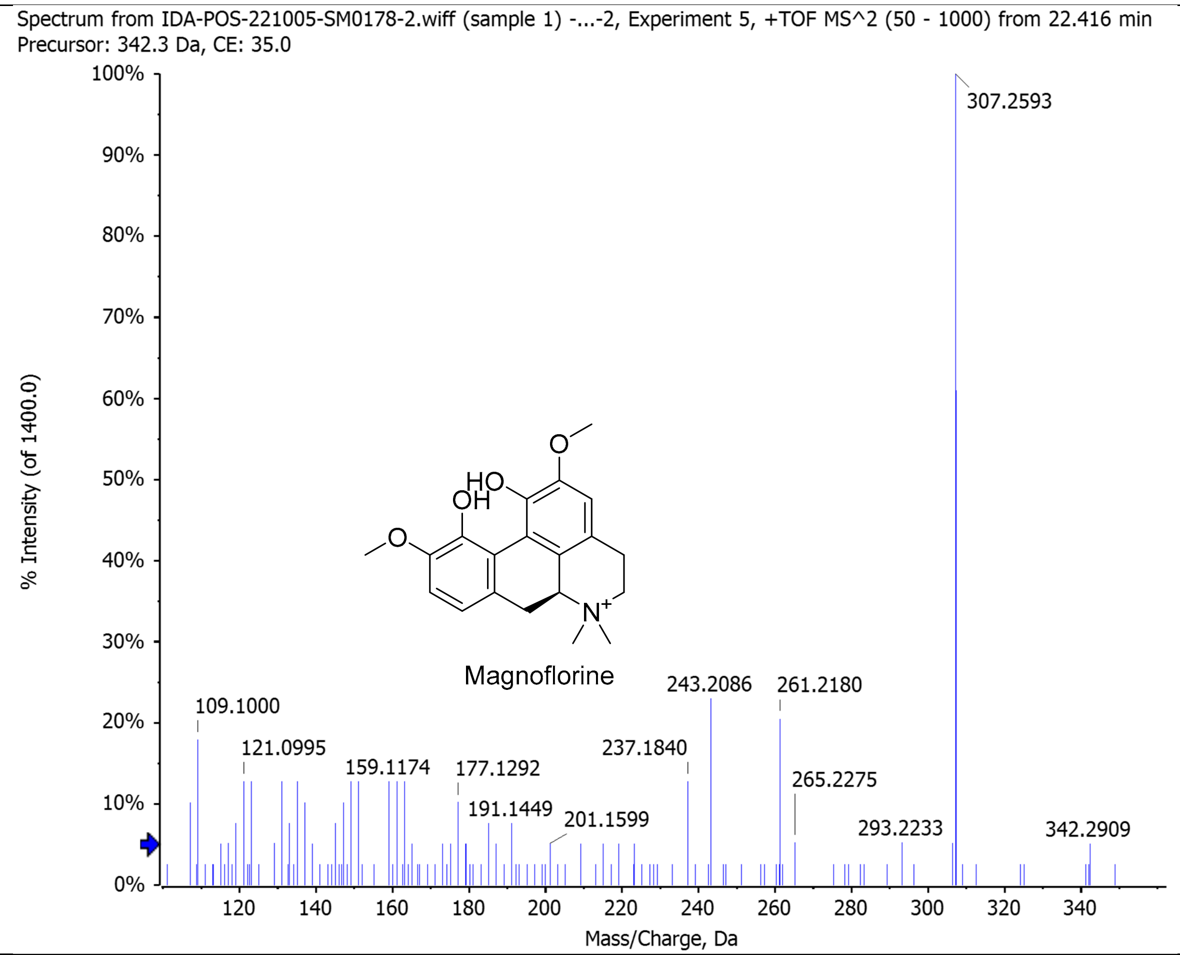

Spectrum from IDA-POS-221005-SM0178-2.wiff (sample 1) -...-2, Experiment 5, +TOF MS^2 (50 - 1000) from 22.416 min
Precursor: 342.3 Da, CE: 35.0
% Intensity (of 1400.0)
100%
90%
80%
70%
60%
50%
40%
30%
20%
10%
0%
307.2593
109.1000
121.0995
159.1174
177.1292
191.1449
201.1599
237.1840
243.2086
261.2180
265.2275
293.2233
342.2909
HO
OH
O
O
N+
Magnoflorine
120
140
160
180
200
220
240
260
280
300
320
340
Mass/Charge, Da

Figure S32

Spectrum from IDA-POS-221005-SM0178-2.wiff (sample 1) -..., Experiment 12, +TOF MS^2 (50 - 1000) from 13.062 min
Precursor: 220.1 Da, CE: 35.0

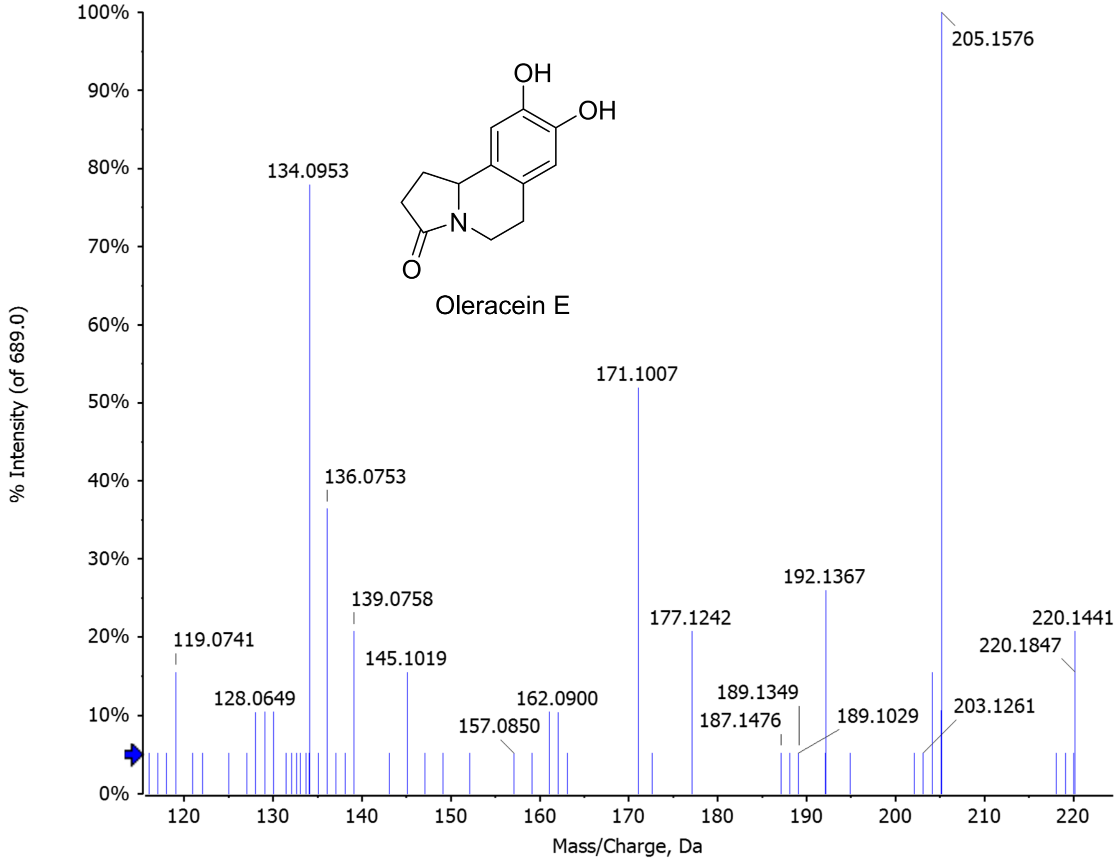

Figure S33

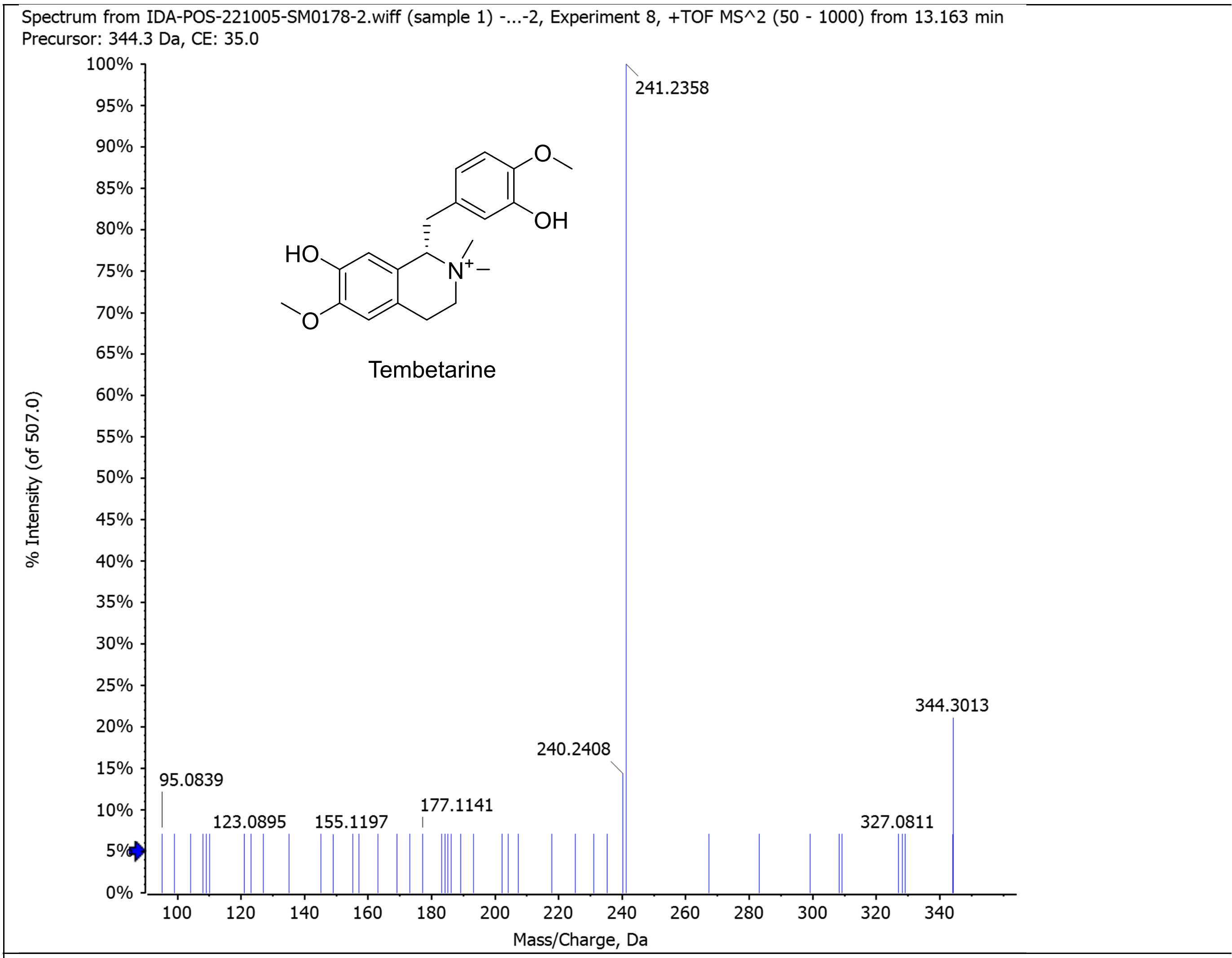

Figure S34 Tembetarine (Alkaloid)

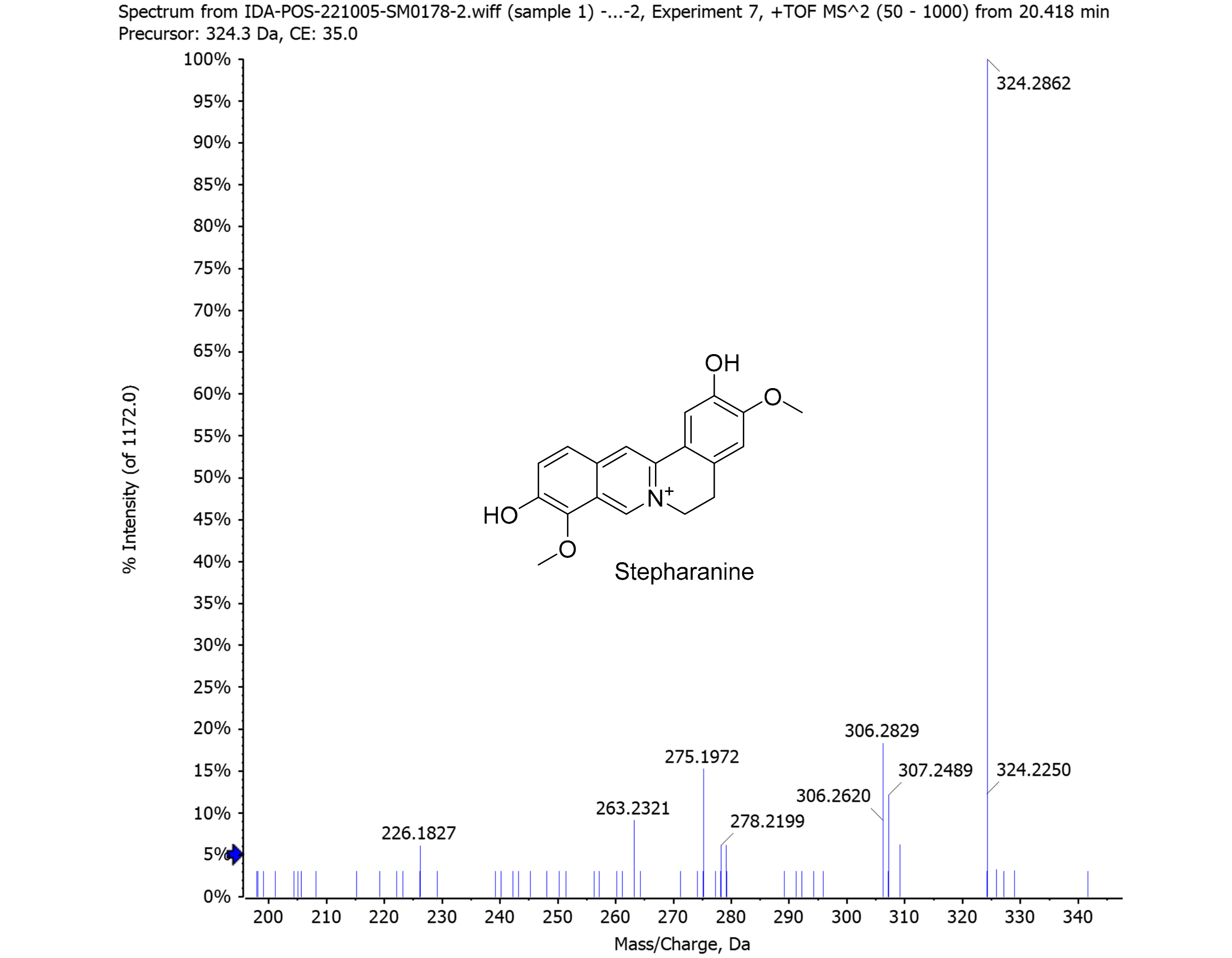
Spectrum from IDA-POS-221005-SM0178-2.wiff (sample 1) -...-2, Experiment 7, +TOF MS^2 (50 - 1000) from 20.418 min
Precursor: 324.3 Da, CE: 35.0
% Intensity (of 1172.0)
Mass/Charge, Da
324.2862
306.2829
275.1972
307.2489
324.2250
306.2620
278.2199
263.2321
226.1827
OH
O
HO
O
N+
Stepharanine

Figure S35

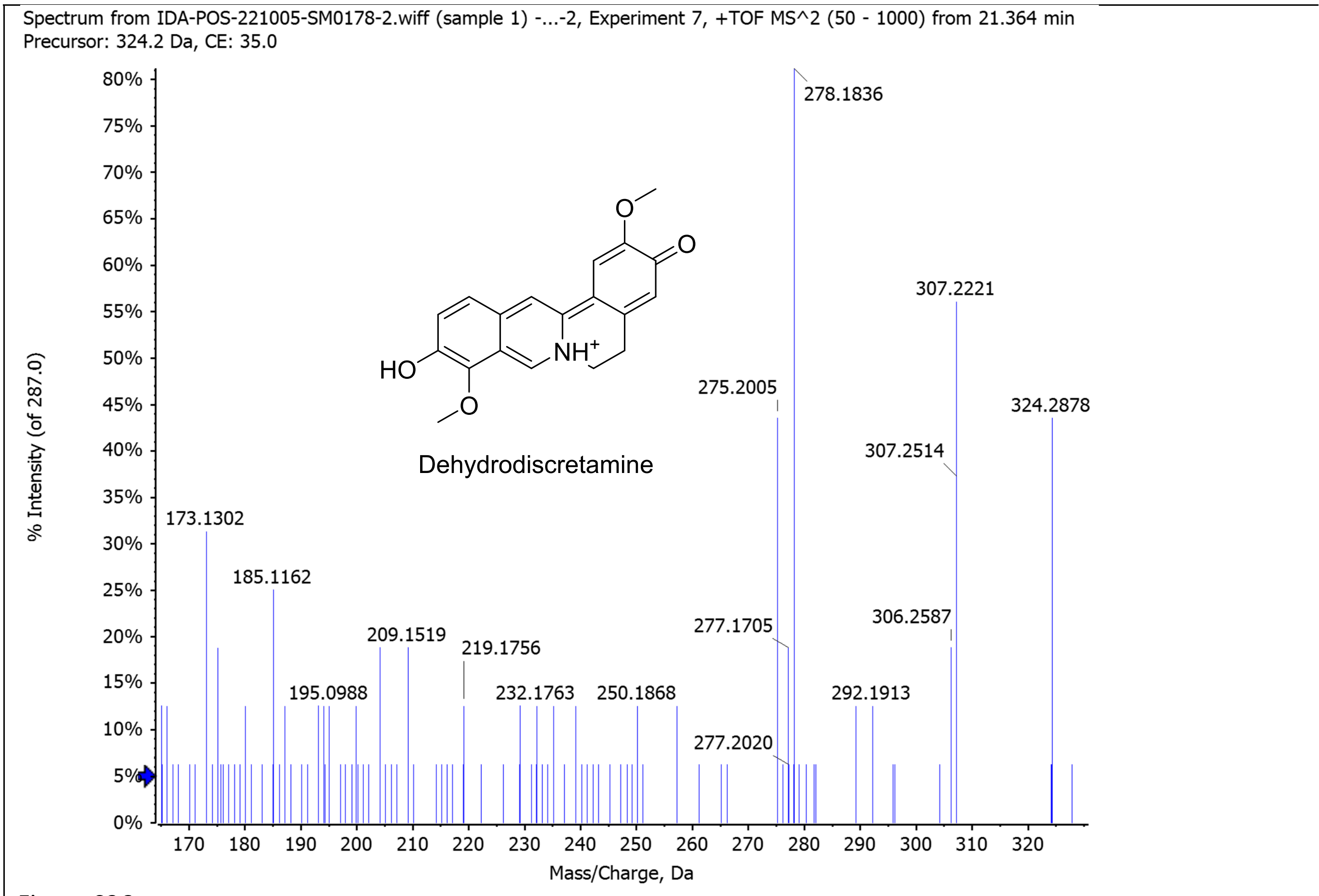
Spectrum from IDA-POS-221005-SM0178-2.wiff (sample 1) -...-2, Experiment 7, +TOF MS^2 (50 - 1000) from 21.364 min
Precursor: 324.2 Da, CE: 35.0
% Intensity (of 287.0)
Mass/Charge, Da
278.1836
307.2221
275.2005
324.2878
307.2514
173.1302
185.1162
209.1519
219.1756
277.1705
306.2587
195.0988
232.1763
250.1868
292.1913
277.2020
HO
O
NH+
Dehydrodiscretamine

Figure S36

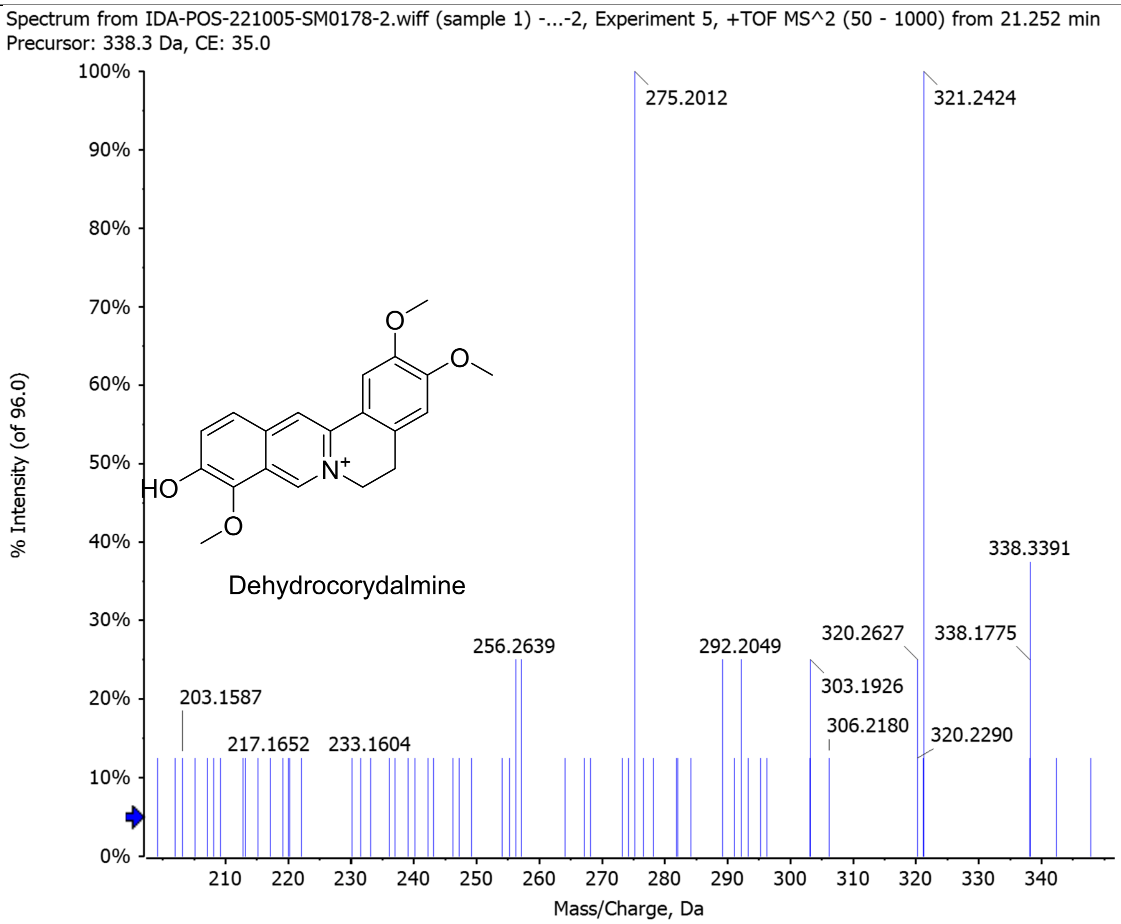
Spectrum from IDA-POS-221005-SM0178-2.wiff (sample 1) -...-2, Experiment 5, +TOF MS^2 (50 - 1000) from 21.252 min
Precursor: 338.3 Da, CE: 35.0
% Intensity (of 96.0)
Mass/Charge, Da
275.2012
321.2424
338.3391
256.2639
292.2049
320.2627
338.1775
303.1926
306.2180
320.2290
203.1587
217.1652
233.1604
HO
O
N+
Dehydrocorydalmine

Figure S37

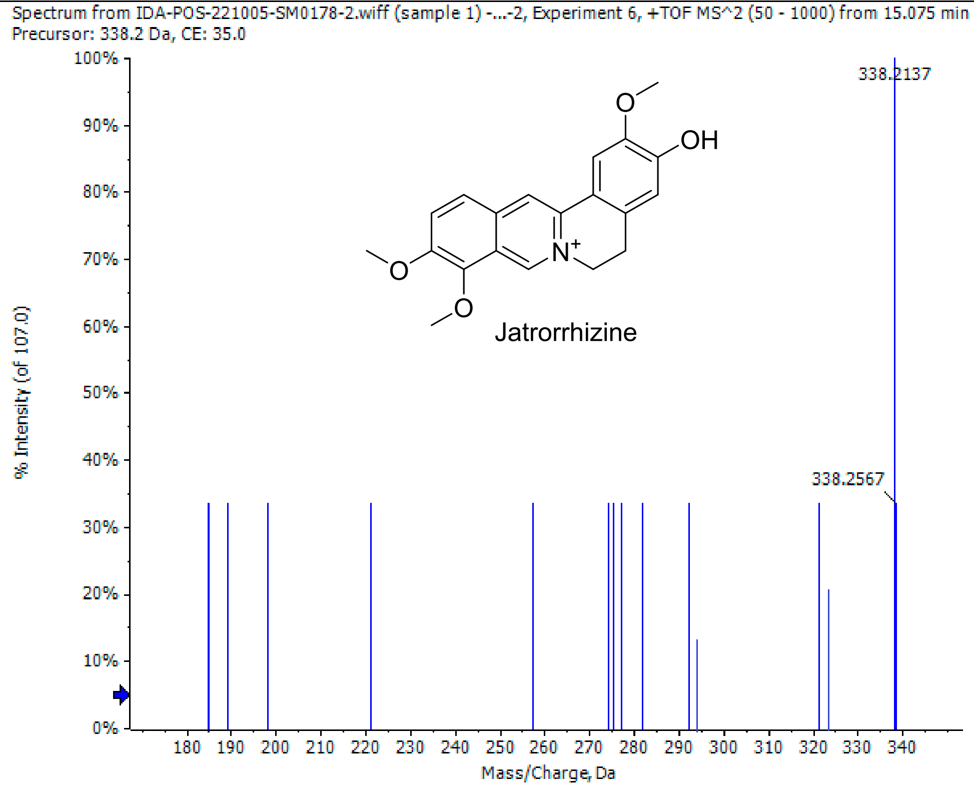
Spectrum from IDA-POS-221005-SM0178-2.wiff (sample 1) -...-2, Experiment 6, +TOF MS^2 (50 - 1000) from 15.075 min
Precursor: 338.2 Da, CE: 35.0
338.2137
338.2567
Jatrorrhizine
OH
O
N+
% Intensity (of 107.0)
Mass/Charge, Da
100%
90%
80%
70%
60%
50%
40%
30%
20%
10%
0%
180
190
200
210
220
230
240
250
260
270
280
290
300
310
320
330
340

Figure S38

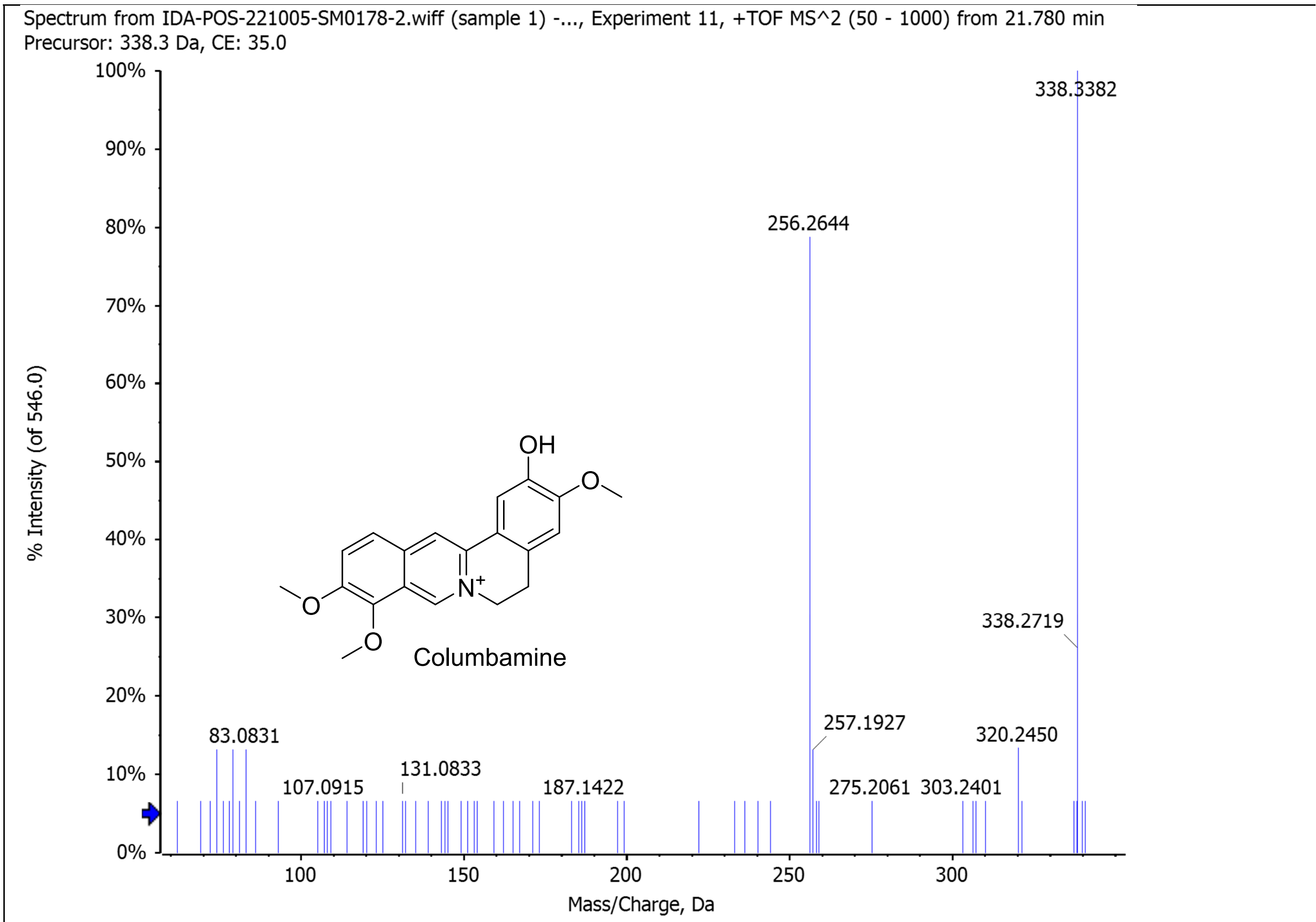
Spectrum from IDA-POS-221005-SM0178-2.wiff (sample 1) -..., Experiment 11, +TOF MS^2 (50 - 1000) from 21.780 min
Precursor: 338.3 Da, CE: 35.0
100%
90%
80%
70%
60%
50%
40%
30%
20%
10%
0%
% Intensity (of 546.0)
338.3382
256.2644
338.2719
257.1927
320.2450
83.0831
131.0833
107.0915
187.1422
275.2061
303.2401
OH
O
N+
O
O
Columbamine
100
150
200
250
300
Mass/Charge, Da
Figure S39

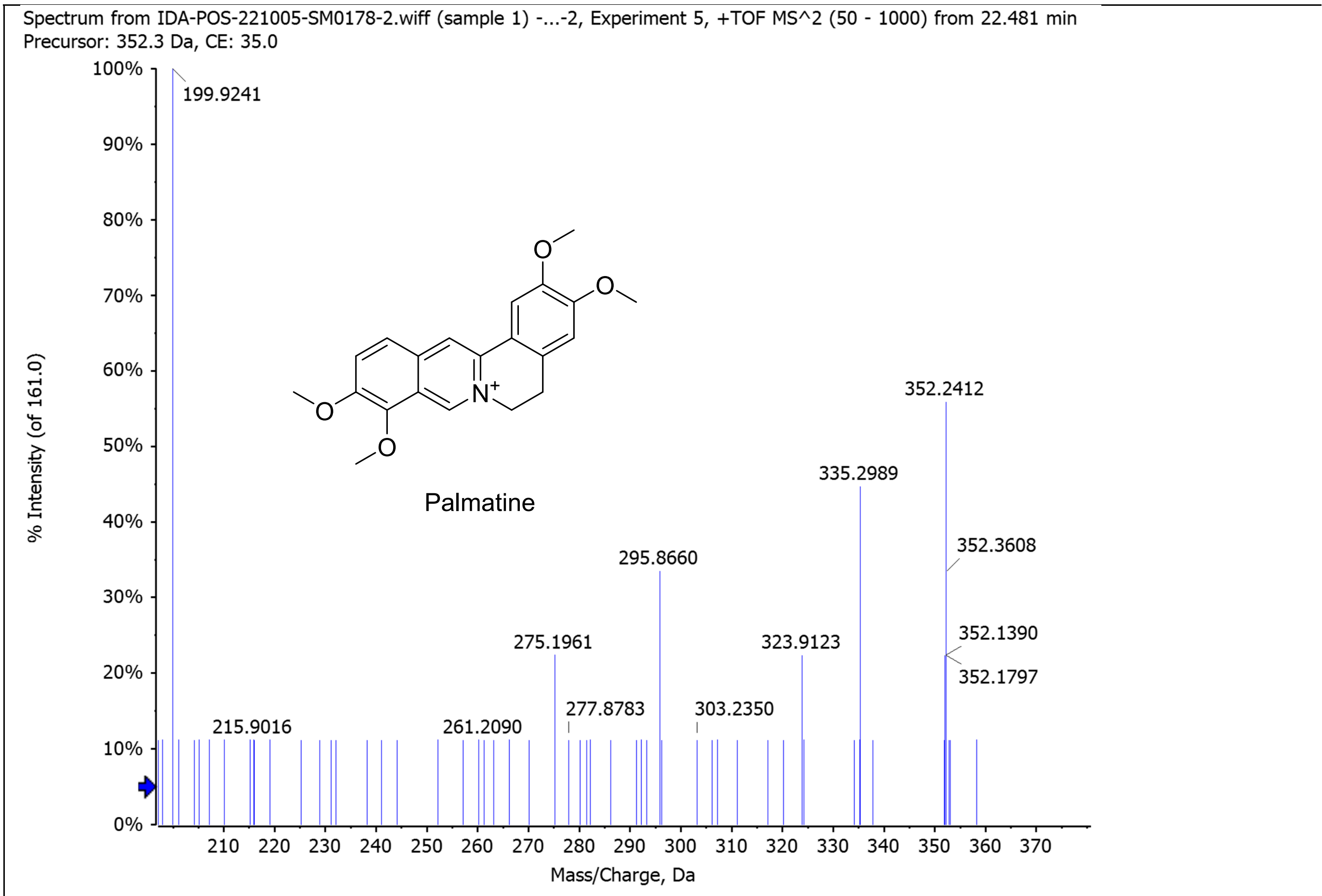
Spectrum from IDA-POS-221005-SM0178-2.wiff (sample 1) -...-2, Experiment 5, +TOF MS^2 (50 - 1000) from 22.481 min
Precursor: 352.3 Da, CE: 35.0
% Intensity (of 161.0)
100%
90%
80%
70%
60%
50%
40%
30%
20%
10%
0%
199.9241
215.9016
261.2090
275.1961
277.8783
295.8660
303.2350
323.9123
335.2989
352.2412
352.3608
352.1390
352.1797
Palmatine
210
220
230
240
250
260
270
280
290
300
310
320
330
340
350
360
370
Mass/Charge, Da

Figure S40

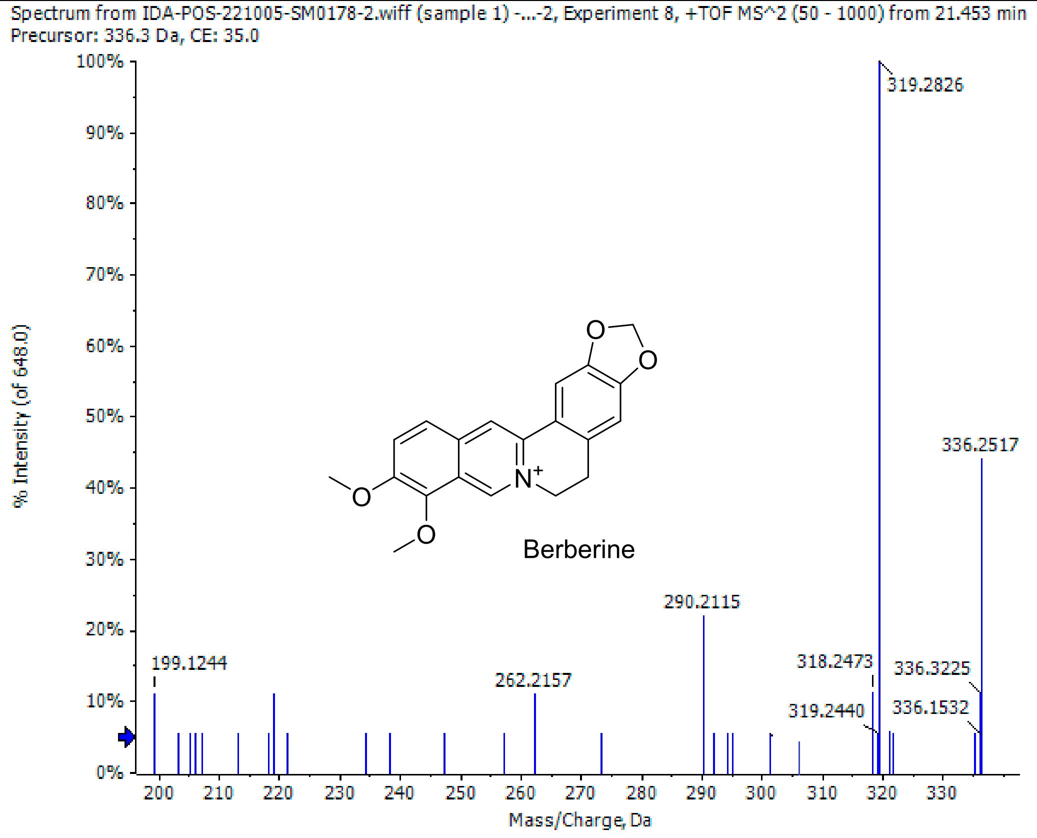

Spectrum from IDA-POS-221005-SM0178-2.wiff (sample 1) -...-2, Experiment 8, +TOF MS^2 (50 - 1000) from 21.453 min
Precursor: 336.3 Da, CE: 35.0
% Intensity (of 648.0)
Mass/Charge, Da
319.2826
336.2517
290.2115
199.1244
262.2157
318.2473
319.2440
336.3225
336.1532
Berberine

Figure S41

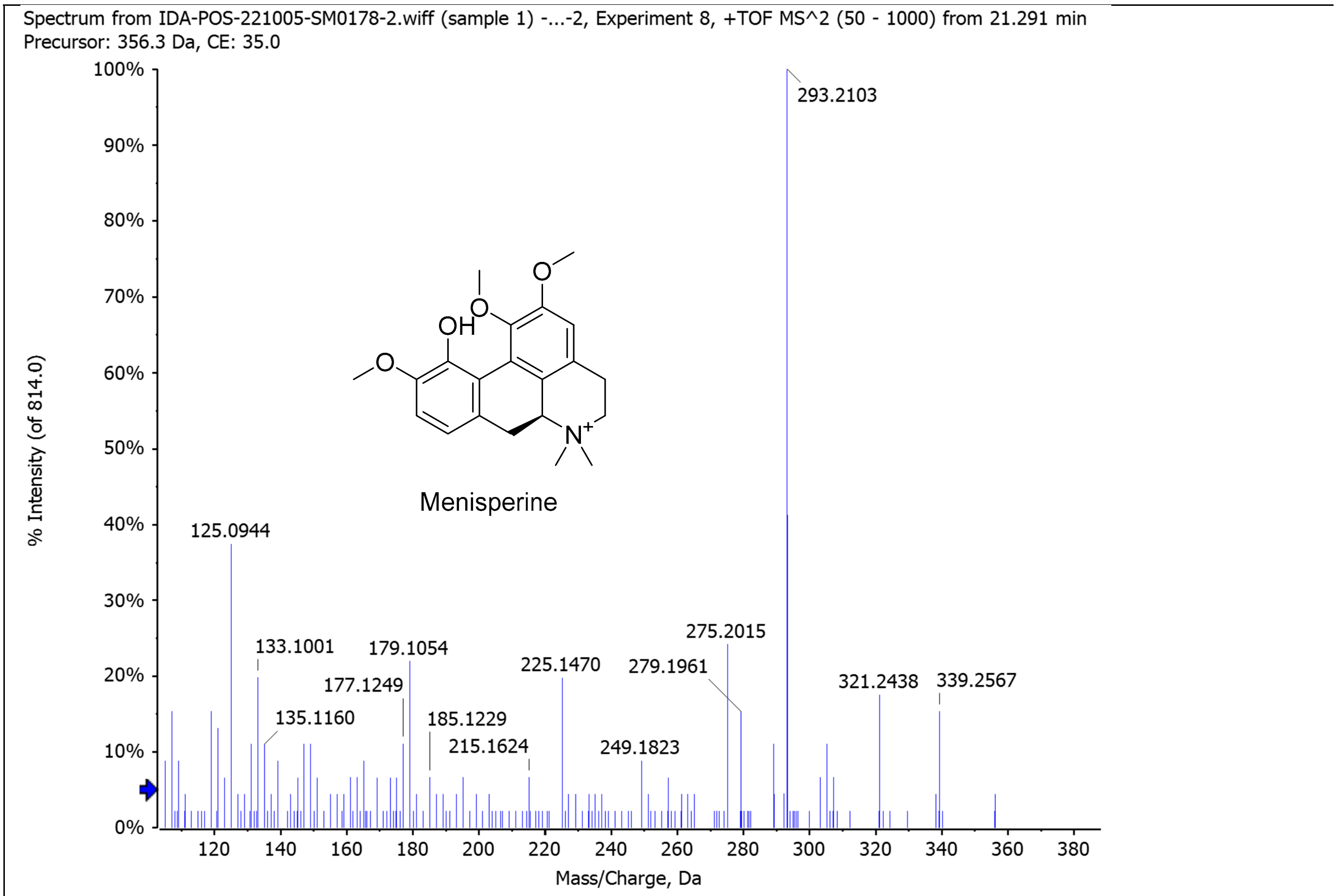

Spectrum from IDA-POS-221005-SM0178-2.wiff (sample 1) -...-2, Experiment 8, +TOF MS^2 (50 - 1000) from 21.291 min
Precursor: 356.3 Da, CE: 35.0
% Intensity (of 814.0)
Mass/Charge, Da
293.2103
125.0944
133.1001
135.1160
177.1249
179.1054
185.1229
215.1624
225.1470
249.1823
275.2015
279.1961
321.2438
339.2567
OH
O
N+
Menisperine

Figure S42

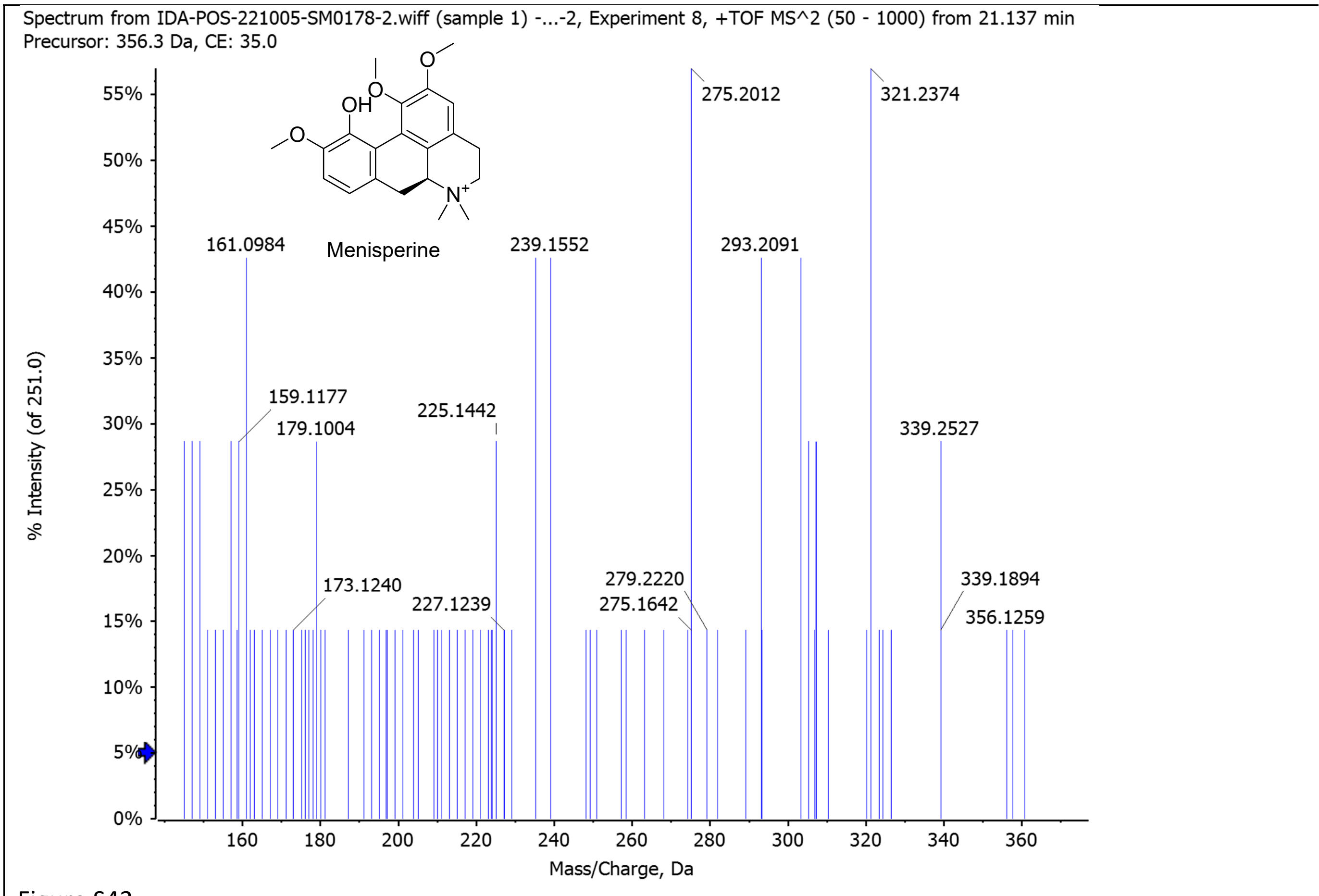

Spectrum from IDA-POS-221005-SM0178-2.wiff (sample 1) -...-2, Experiment 8, +TOF MS^2 (50 - 1000) from 21.137 min
Precursor: 356.3 Da, CE: 35.0
OH
O
O
O
N+
Menisperine
% Intensity (of 251.0)
Mass/Charge, Da
161.0984
159.1177
179.1004
173.1240
225.1442
227.1239
239.1552
275.2012
279.2220
275.1642
293.2091
321.2374
339.2527
339.1894
356.1259

Figure S43

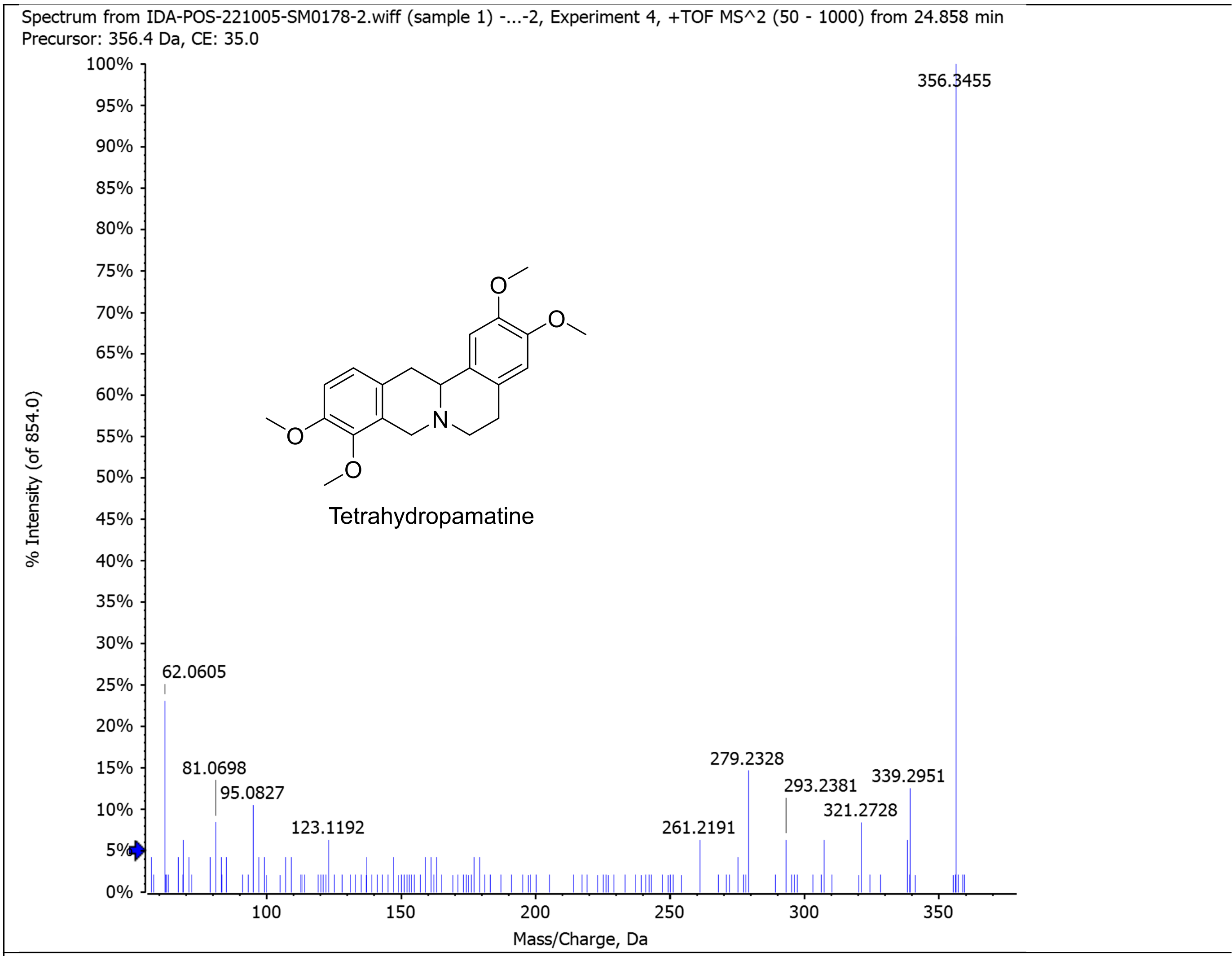

Spectrum from IDA-POS-221005-SM0178-2.wiff (sample 1) -...-2, Experiment 4, +TOF MS^2 (50 - 1000) from 24.858 min
Precursor: 356.4 Da, CE: 35.0
% Intensity (of 854.0)
Mass/Charge, Da
356.3455
62.0605
81.0698
95.0827
123.1192
261.2191
279.2328
293.2381
321.2728
339.2951
Tetrahydropamatine

Figure S44

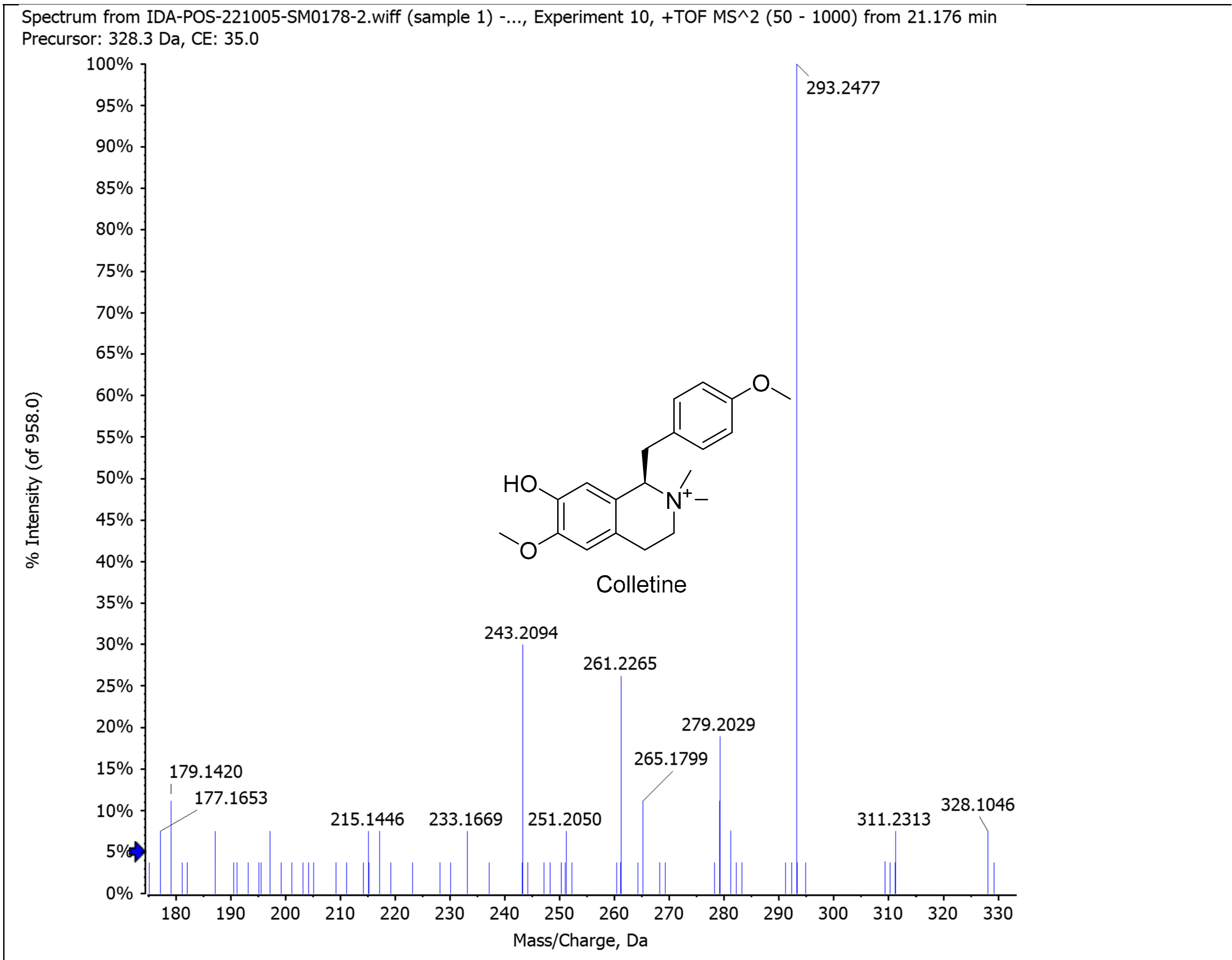
Spectrum from IDA-POS-221005-SM0178-2.wiff (sample 1) -..., Experiment 10, +TOF MS^2 (50 - 1000) from 21.176 min
Precursor: 328.3 Da, CE: 35.0
% Intensity (of 958.0)
Mass/Charge, Da
293.2477
243.2094
261.2265
279.2029
265.1799
179.1420
177.1653
215.1446
233.1669
251.2050
311.2313
328.1046
HO
O
N+
Colletine

Figure S45

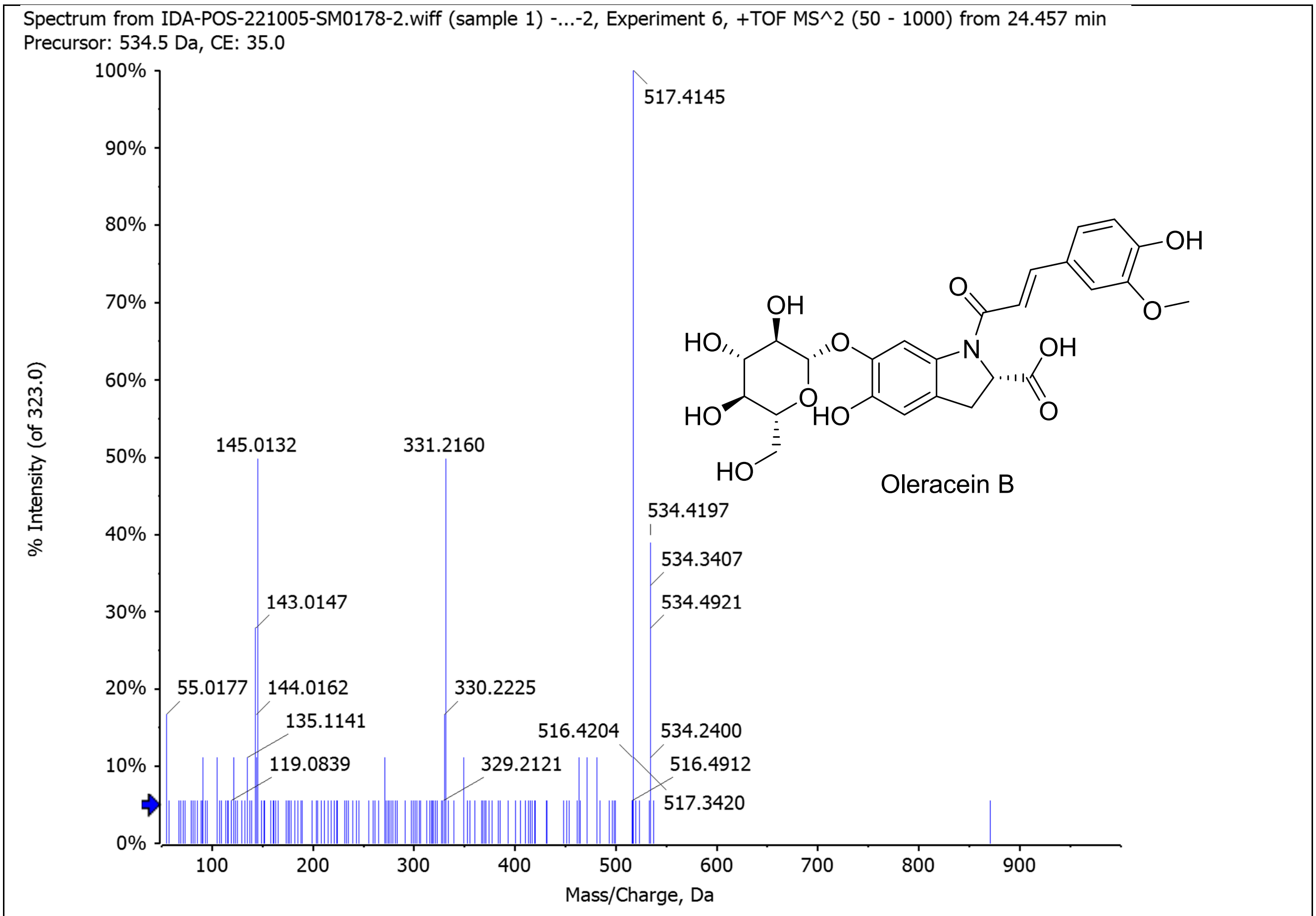

Spectrum from IDA-POS-221005-SM0178-2.wiff (sample 1) -...-2, Experiment 6, +TOF MS^2 (50 - 1000) from 24.457 min
Precursor: 534.5 Da, CE: 35.0
% Intensity (of 323.0)
Mass/Charge, Da
517.4145
145.0132
331.2160
534.4197
534.3407
534.4921
143.0147
55.0177
144.0162
330.2225
135.1141
516.4204
534.2400
119.0839
329.2121
516.4912
517.3420
Oleracein B

Figure S46

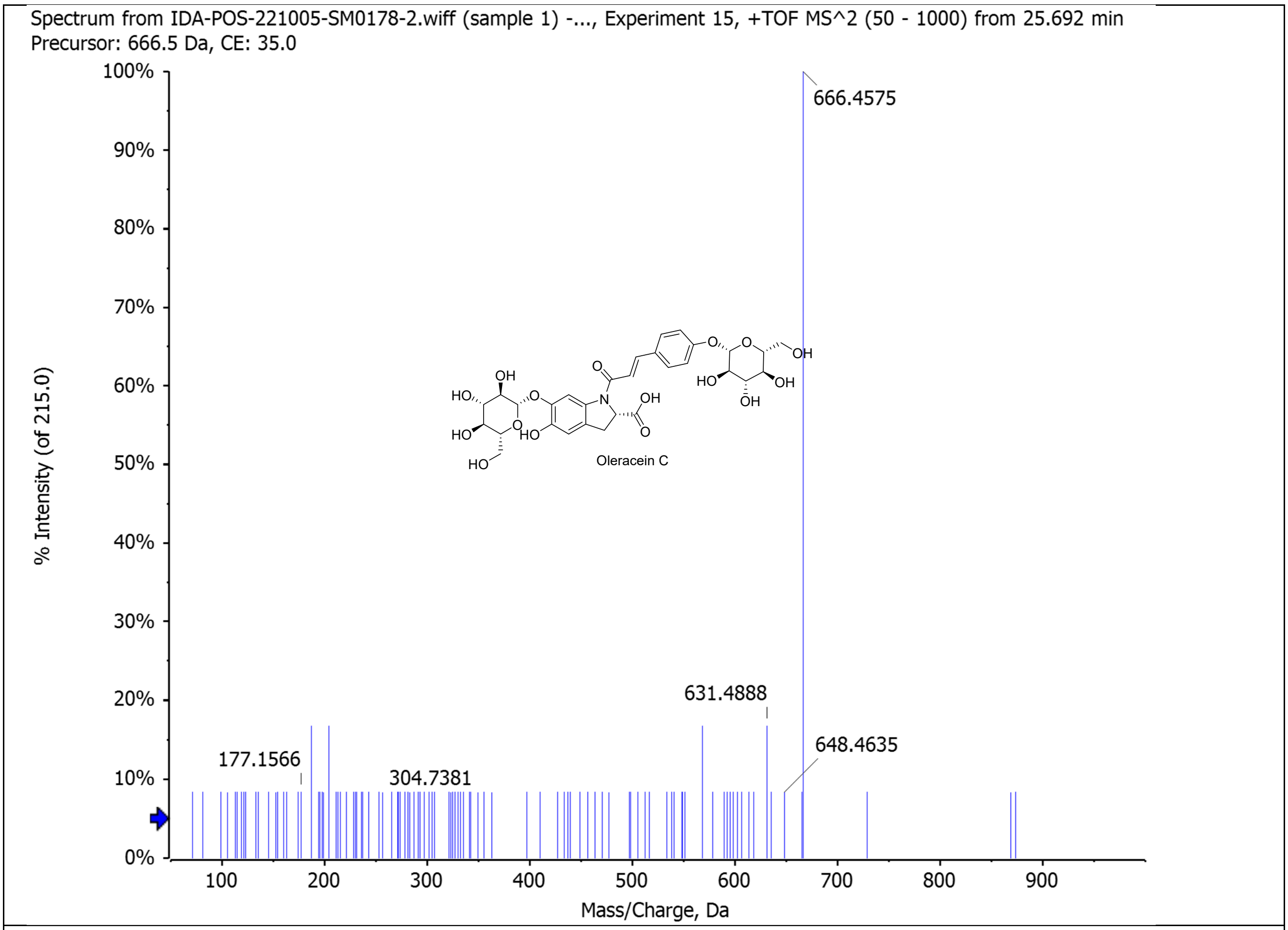

Spectrum from IDA-POS-221005-SM0178-2.wiff (sample 1) -..., Experiment 15, +TOF MS^2 (50 - 1000) from 25.692 min
Precursor: 666.5 Da, CE: 35.0
666.4575
631.4888
648.4635
177.1566
304.7381
% Intensity (of 215.0)
Mass/Charge, Da
Oleracein C

Figure S47

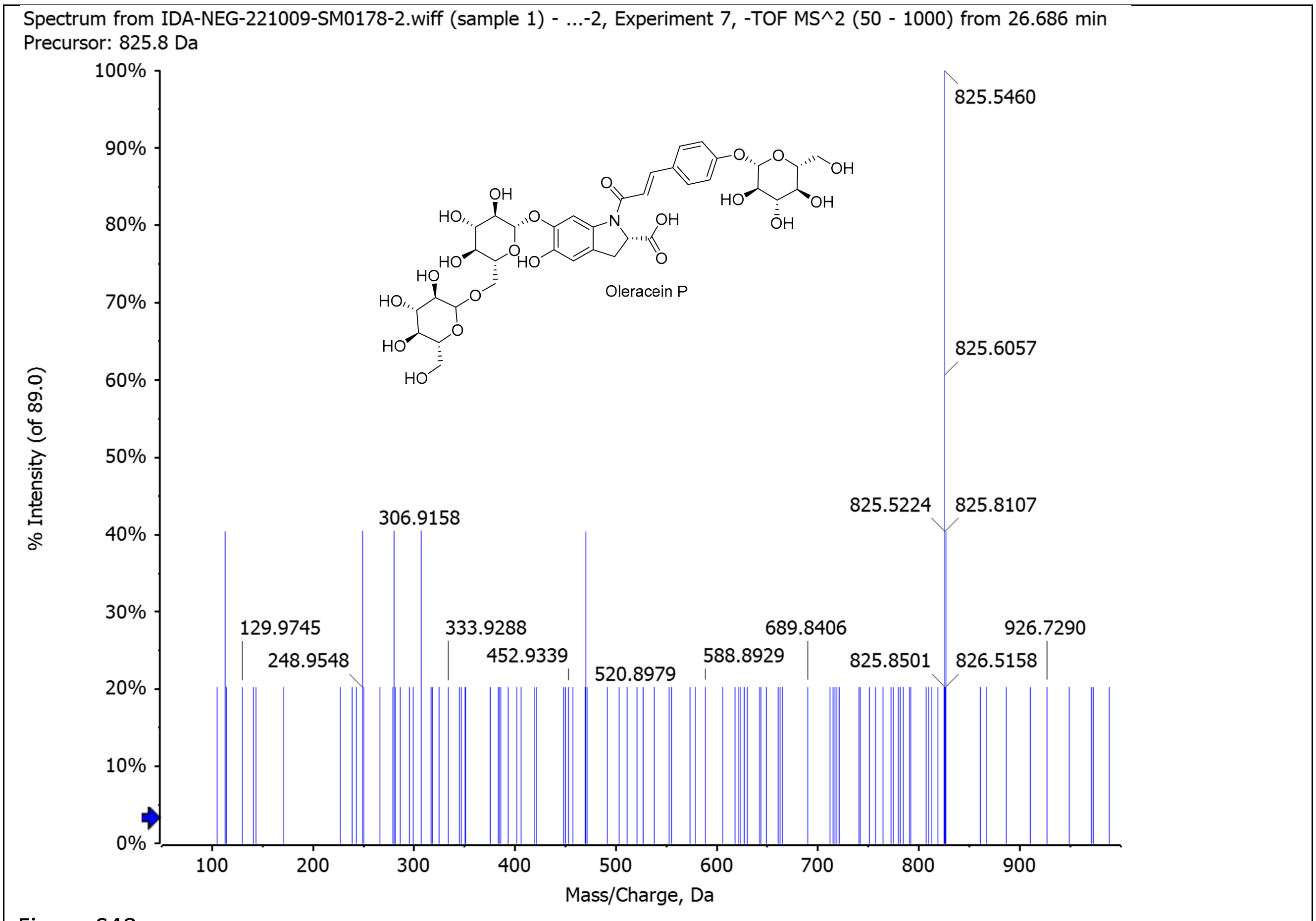
Spectrum from IDA-NEG-221009-SM0178-2.wiff (sample 1) - ...-2, Experiment 7, -TOF MS^2 (50 - 1000) from 26.686 min
Precursor: 825.8 Da
% Intensity (of 89.0)
Mass/Charge, Da
825.5460
825.6057
825.5224
825.8107
306.9158
129.9745
333.9288
689.8406
926.7290
248.9548
452.9339
588.8929
825.8501
826.5158
520.8979
Oleracein P

Figure S48

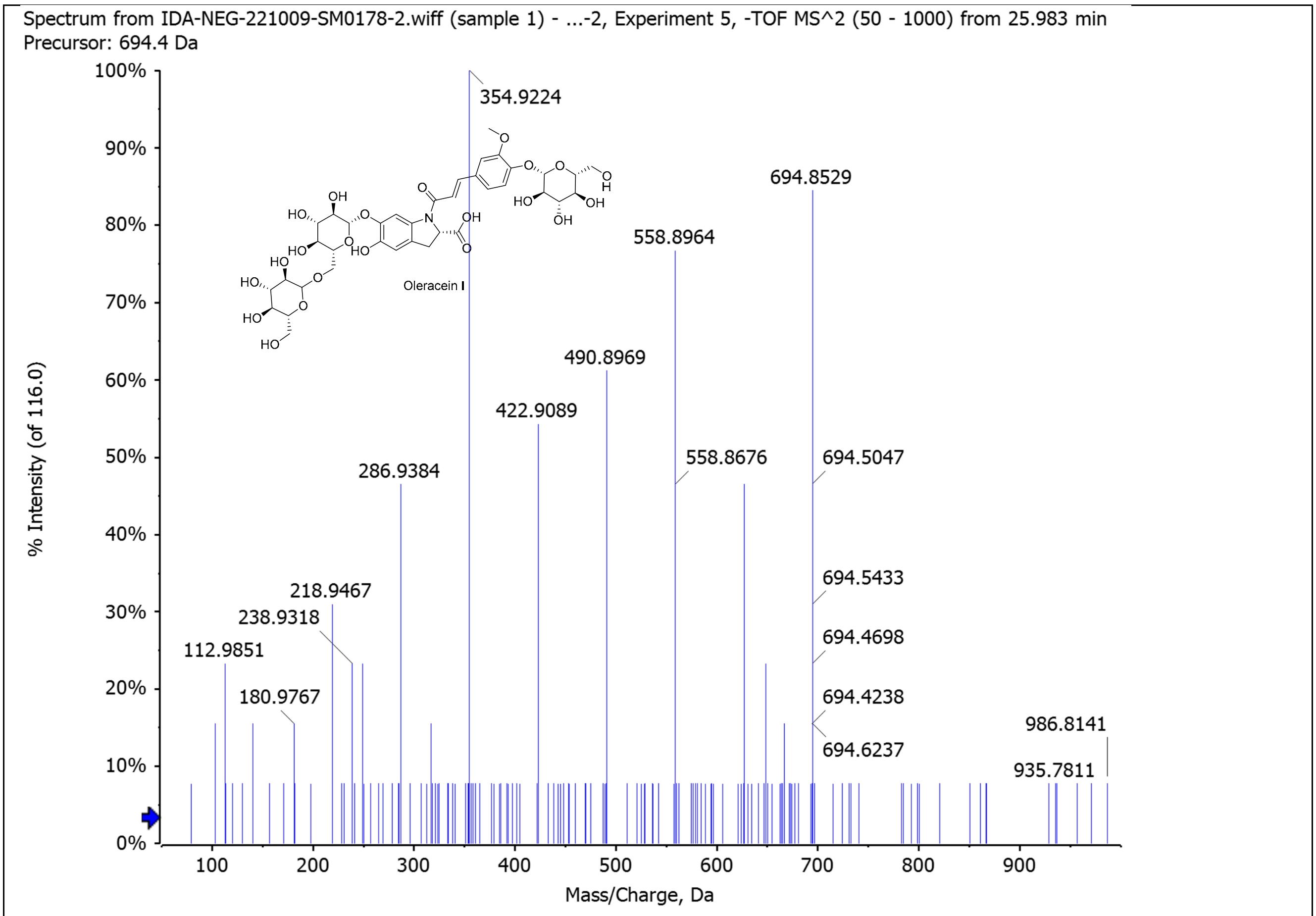
Spectrum from IDA-NEG-221009-SM0178-2.wiff (sample 1) - ...-2, Experiment 5, -TOF MS^2 (50 - 1000) from 25.983 min
Precursor: 694.4 Da
Oleracein I
354.9224
694.8529
558.8964
490.8969
422.9089
286.9384
558.8676
694.5047
694.5433
218.9467
238.9318
112.9851
694.4698
180.9767
694.4238
986.8141
694.6237
935.7811
% Intensity (of 116.0)
Mass/Charge, Da
Figure S49

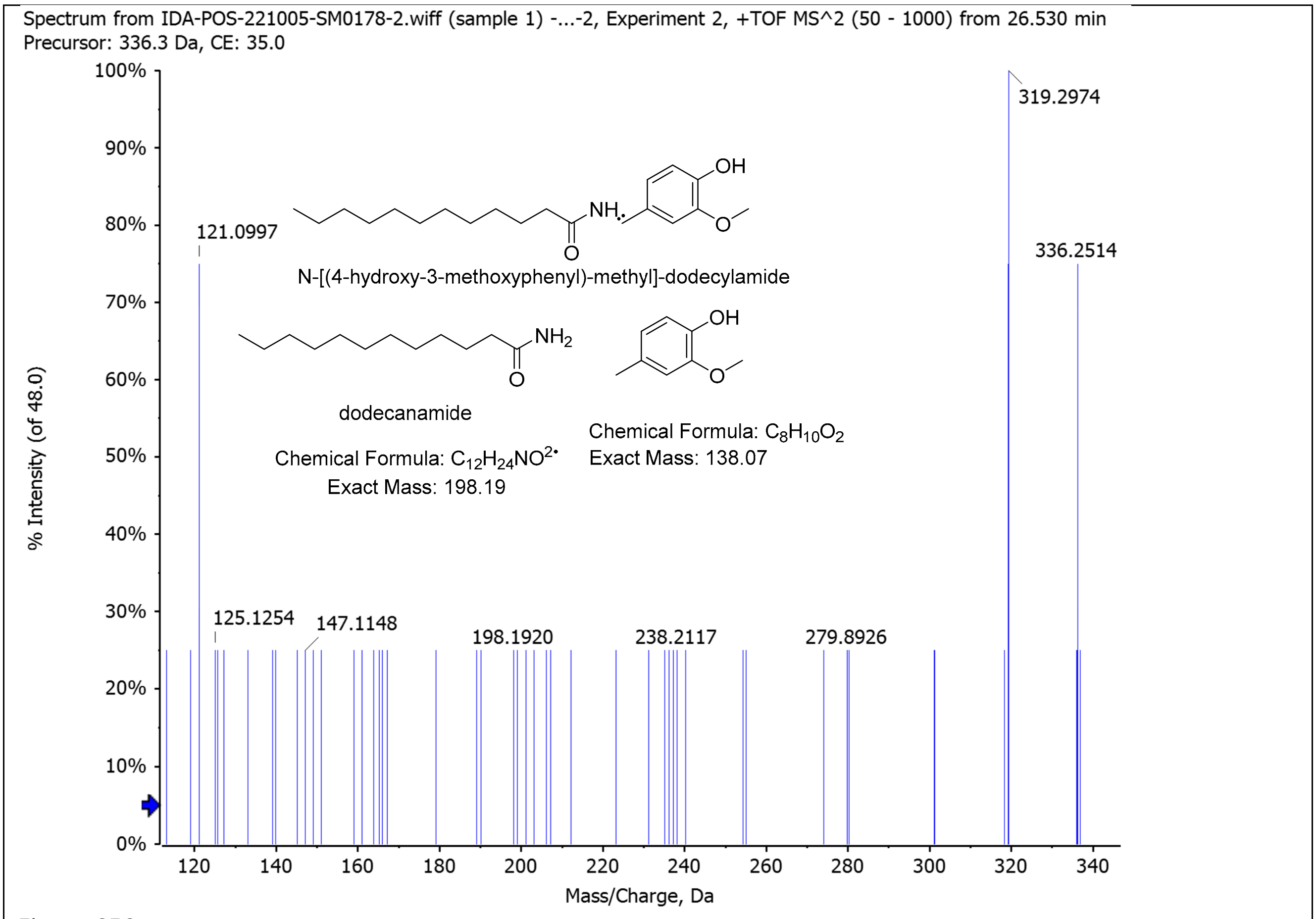
Spectrum from IDA-POS-221005-SM0178-2.wiff (sample 1) -...-2, Experiment 2, +TOF MS^2 (50 - 1000) from 26.530 min
Precursor: 336.3 Da, CE: 35.0
319.2974
336.2514
121.0997
125.1254
147.1148
198.1920
238.2117
279.8926
OH
NH
O
N-[(4-hydroxy-3-methoxyphenyl)-methyl]-dodecylamide
NH_2
dodecanamide
Chemical Formula: $C_{12}H_{24}NO^{2\bullet}$
Exact Mass: 198.19
Chemical Formula: $C_8H_{10}O_2$
Exact Mass: 138.07
% Intensity (of 48.0)
Mass/Charge, Da

Figure S50

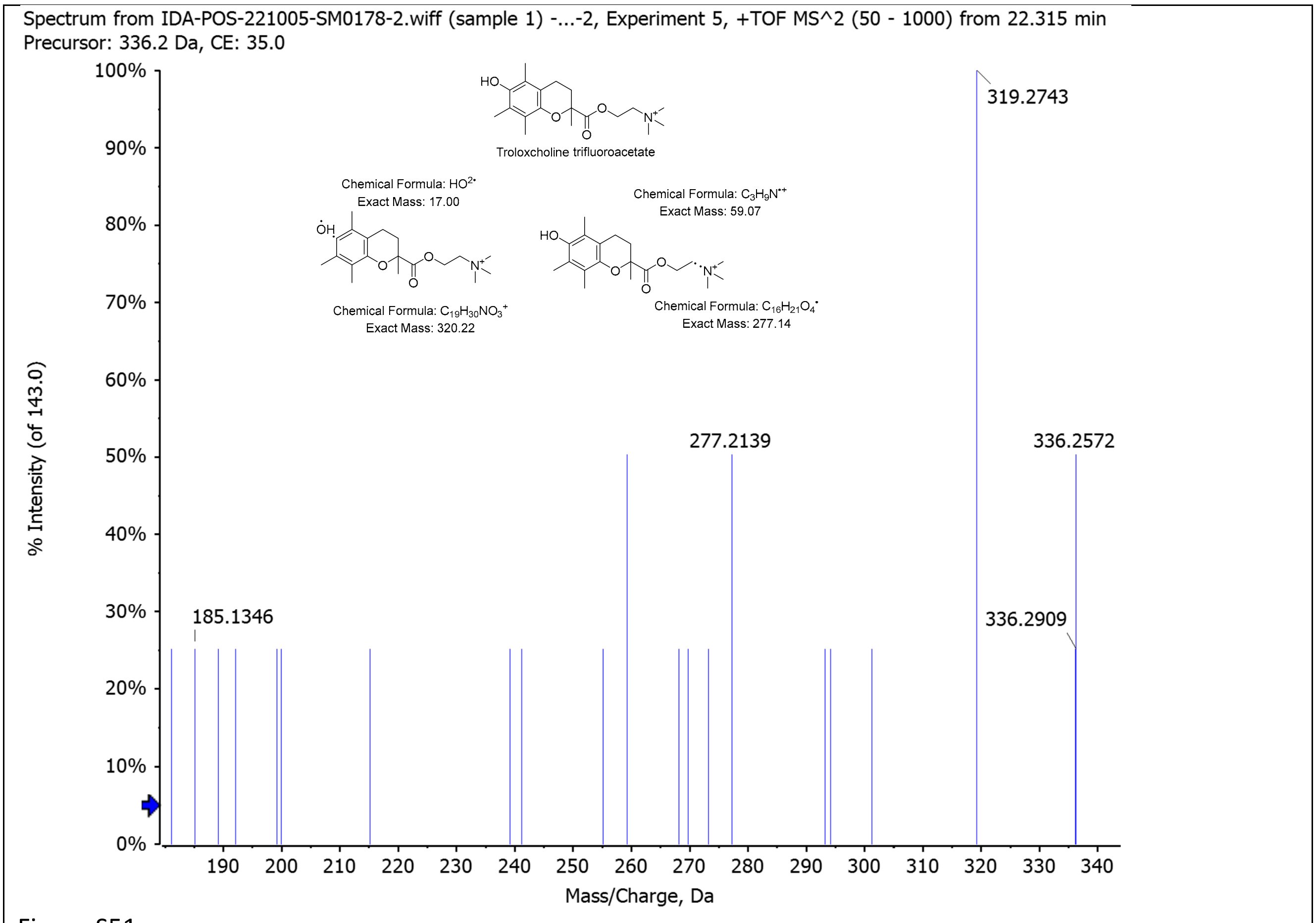

Spectrum from IDA-POS-221005-SM0178-2.wiff (sample 1) -...-2, Experiment 5, +TOF MS^2 (50 - 1000) from 22.315 min
Precursor: 336.2 Da, CE: 35.0
Troloxcholine trifluoroacetate
Chemical Formula: HO2•
Exact Mass: 17.00
Chemical Formula: C3H9N•+
Exact Mass: 59.07
Chemical Formula: C19H30NO3+
Exact Mass: 320.22
Chemical Formula: C16H21O4•
Exact Mass: 277.14
319.2743
277.2139
336.2572
185.1346
336.2909
% Intensity (of 143.0)
Mass/Charge, Da

Figure S51

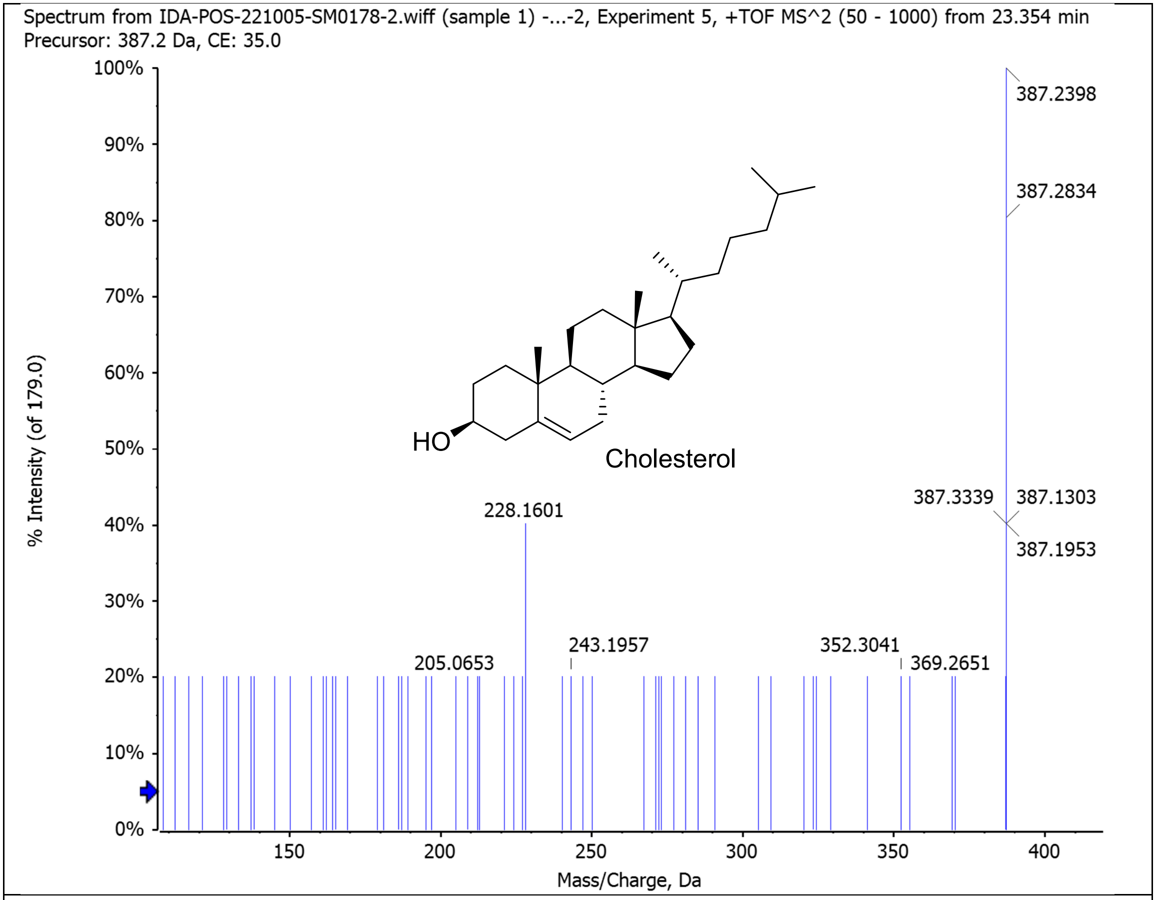

Spectrum from IDA-POS-221005-SM0178-2.wiff (sample 1) -...-2, Experiment 5, +TOF MS^2 (50 - 1000) from 23.354 min
Precursor: 387.2 Da, CE: 35.0
% Intensity (of 179.0)
100%
90%
80%
70%
60%
50%
40%
30%
20%
10%
0%
387.2398
387.2834
HO
Cholesterol
228.1601
387.3339
387.1303
387.1953
243.1957
352.3041
205.0653
369.2651
150
200
250
300
350
400
Mass/Charge, Da

Figure S52

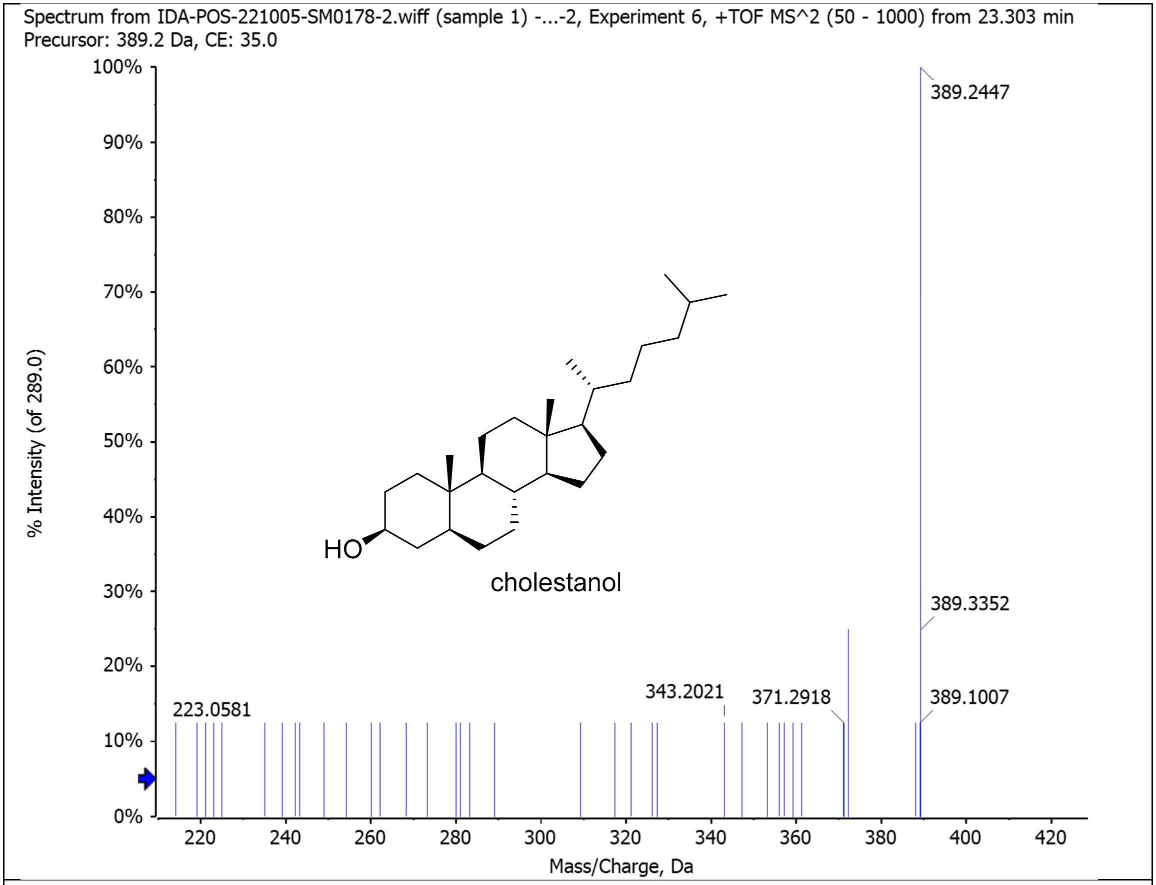
Spectrum from IDA-POS-221005-SM0178-2.wiff (sample 1) -...-2, Experiment 6, +TOF MS^2 (50 - 1000) from 23.303 min
Precursor: 389.2 Da, CE: 35.0
% Intensity (of 289.0)
Mass/Charge, Da
389.2447
389.3352
389.1007
371.2918
343.2021
223.0581
HO
cholestanol

Figure S53

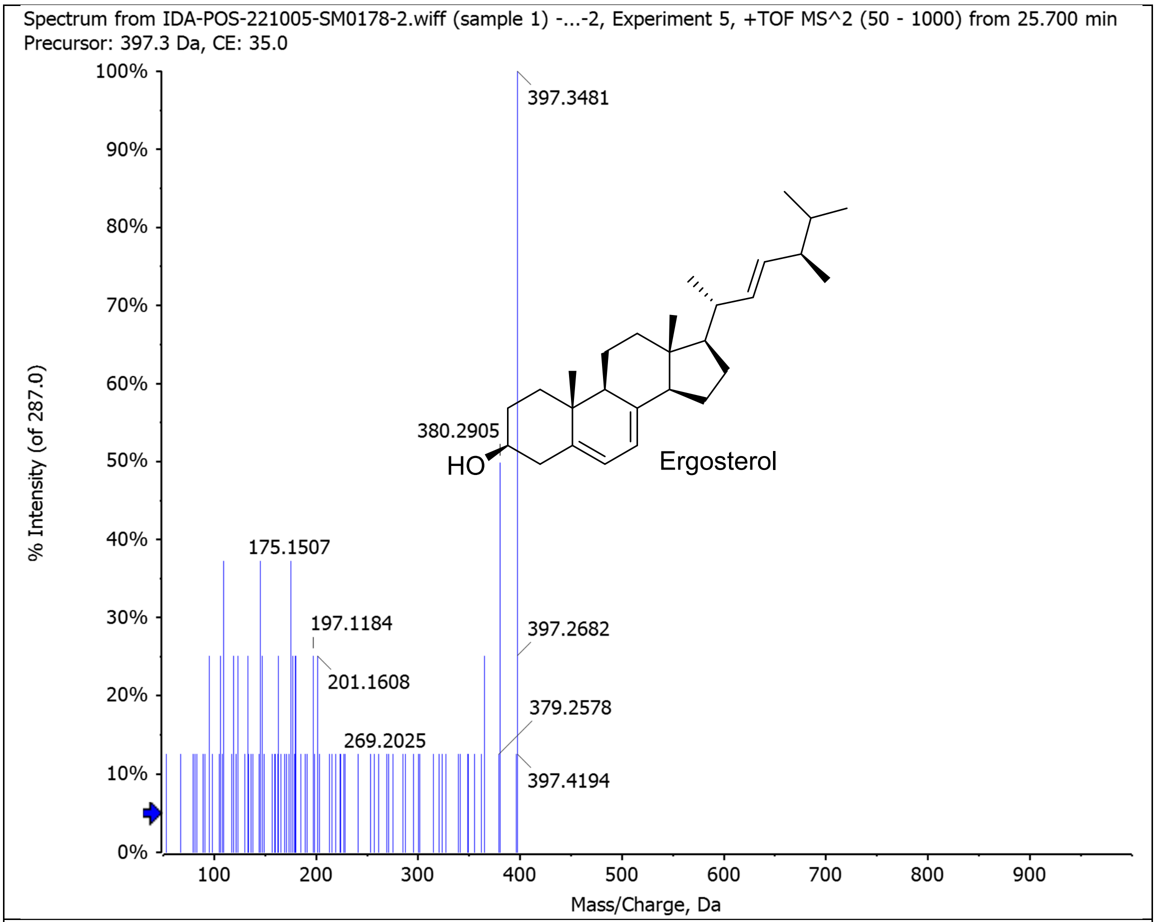

Spectrum from IDA-POS-221005-SM0178-2.wiff (sample 1) -...-2, Experiment 5, +TOF MS^2 (50 - 1000) from 25.700 min
Precursor: 397.3 Da, CE: 35.0
100%
90%
80%
70%
60%
50%
40%
30%
20%
10%
0%
% Intensity (of 287.0)
397.3481
380.2905
175.1507
197.1184
201.1608
269.2025
397.2682
379.2578
397.4194
HO
Ergosterol
100
200
300
400
500
600
700
800
900
Mass/Charge, Da

Figure S54

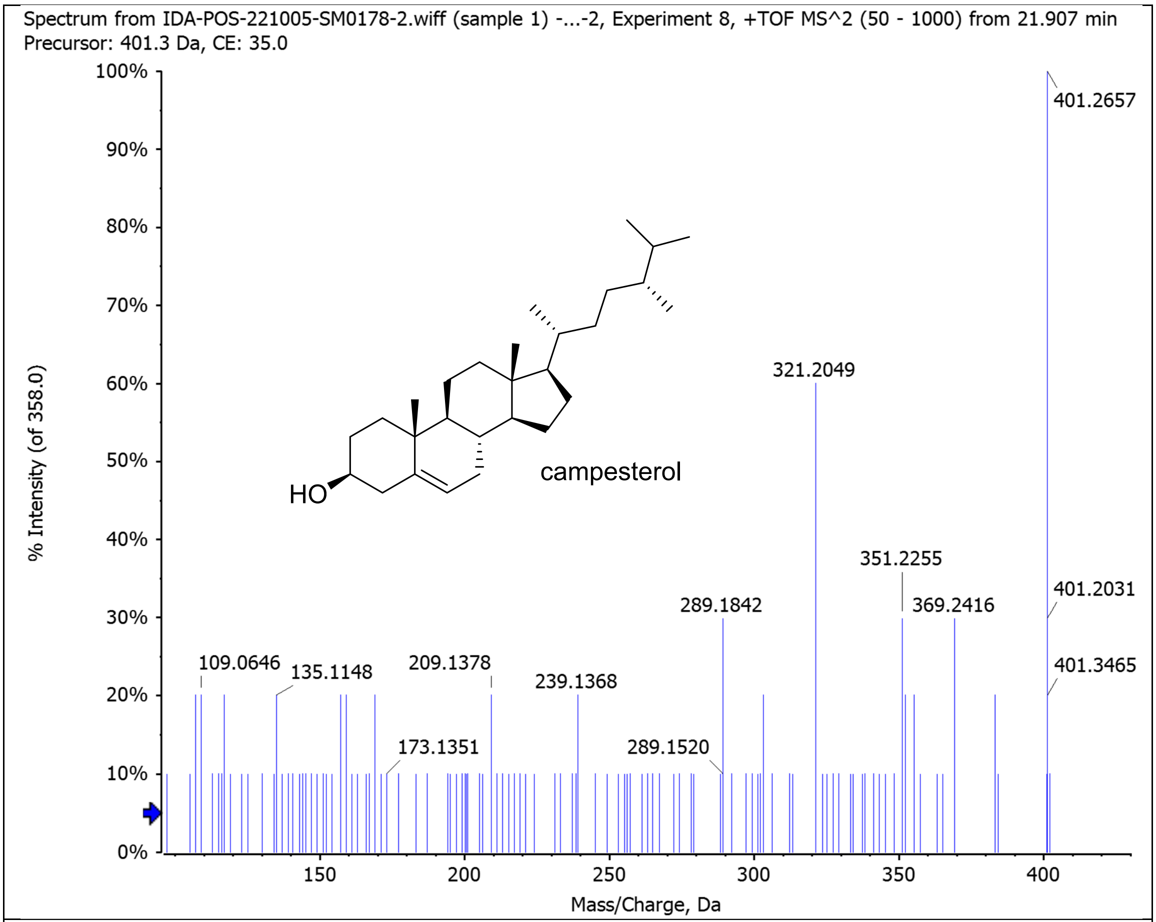
Spectrum from IDA-POS-221005-SM0178-2.wiff (sample 1) -...-2, Experiment 8, +TOF MS^2 (50 - 1000) from 21.907 min
Precursor: 401.3 Da, CE: 35.0
% Intensity (of 358.0)
Mass/Charge, Da
campesterol
HO
401.2657
321.2049
351.2255
369.2416
401.2031
289.1842
401.3465
109.0646
135.1148
209.1378
239.1368
173.1351
289.1520

Figure S55

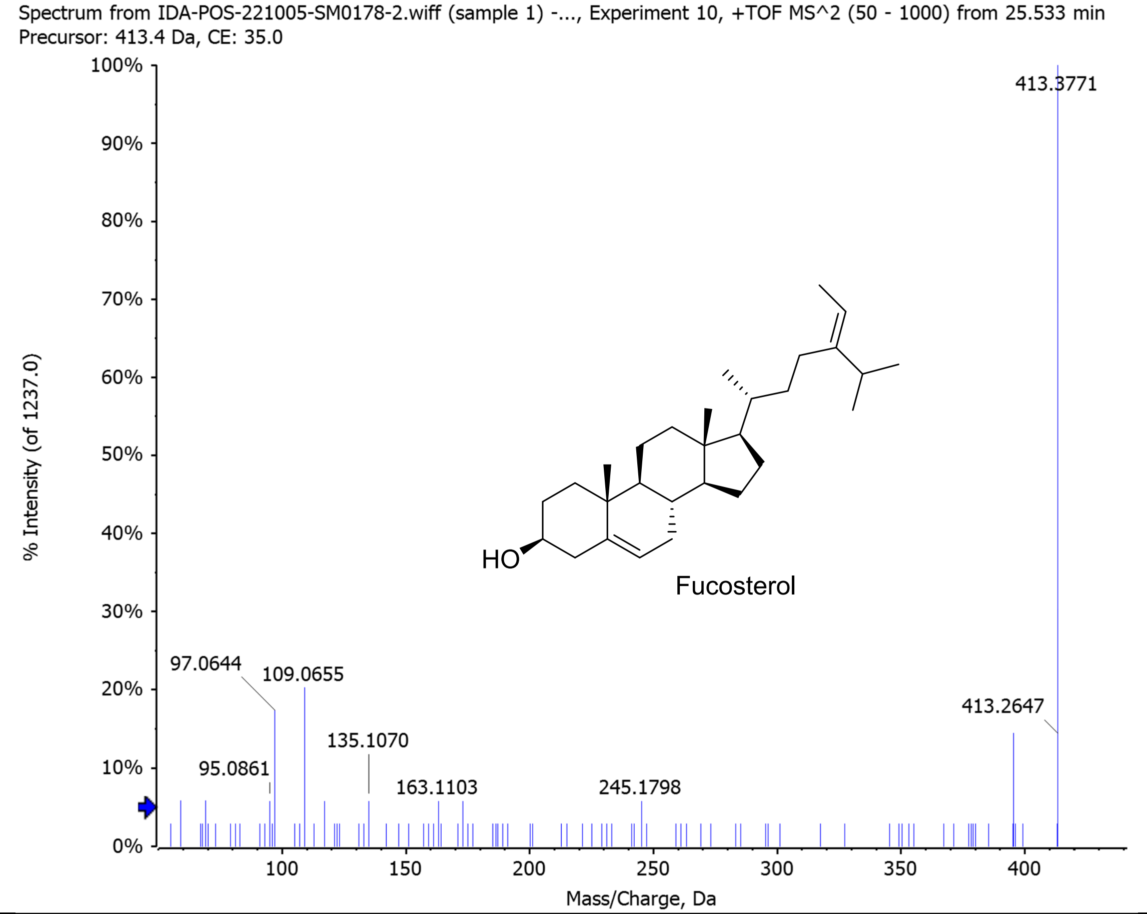

Spectrum from IDA-POS-221005-SM0178-2.wiff (sample 1) -..., Experiment 10, +TOF MS^2 (50 - 1000) from 25.533 min
Precursor: 413.4 Da, CE: 35.0
413.3771
413.2647
97.0644
109.0655
95.0861
135.1070
163.1103
245.1798
HO
Fucosterol
% Intensity (of 1237.0)
Mass/Charge, Da

Figure S56

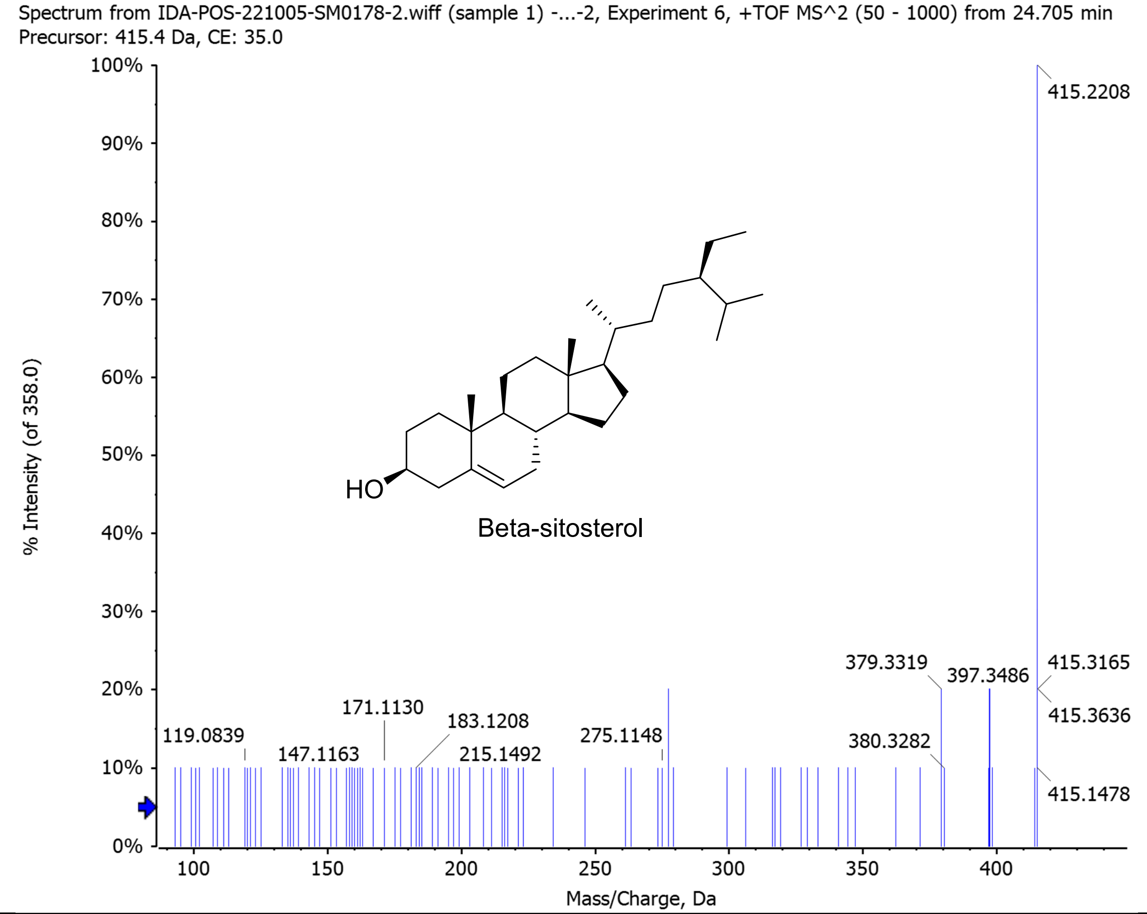

Spectrum from IDA-POS-221005-SM0178-2.wiff (sample 1) -...-2, Experiment 6, +TOF MS^2 (50 - 1000) from 24.705 min
Precursor: 415.4 Da, CE: 35.0
% Intensity (of 358.0)
Mass/Charge, Da
415.2208
415.3165
415.3636
415.1478
379.3319
397.3486
380.3282
119.0839
147.1163
171.1130
183.1208
215.1492
275.1148
HO
Beta-sitosterol

Figure S57

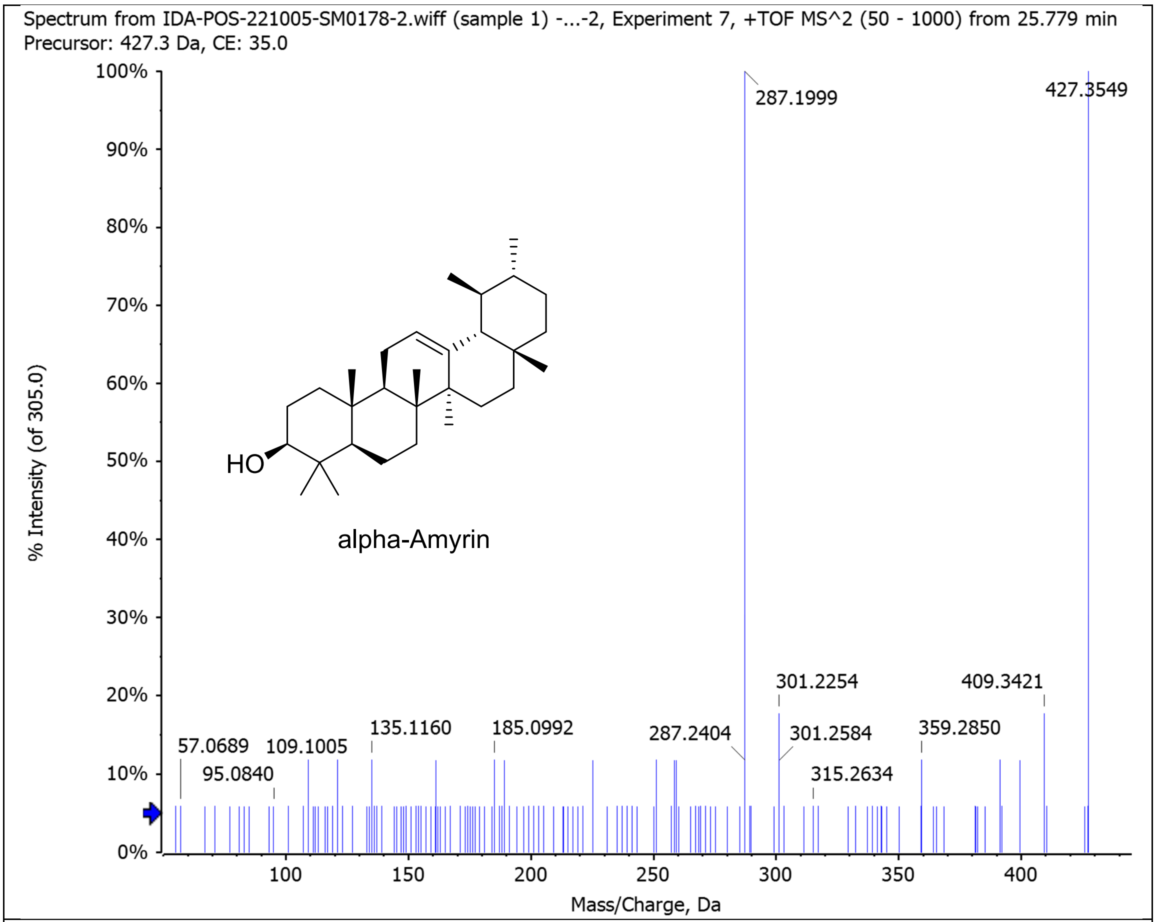

Spectrum from IDA-POS-221005-SM0178-2.wiff (sample 1) -...-2, Experiment 7, +TOF MS^2 (50 - 1000) from 25.779 min
Precursor: 427.3 Da, CE: 35.0
% Intensity (of 305.0)
100%
90%
80%
70%
60%
50%
40%
30%
20%
10%
0%
287.1999
427.3549
HO
alpha-Amyrin
301.2254
409.3421
57.0689
109.1005
135.1160
185.0992
287.2404
301.2584
359.2850
95.0840
315.2634
100
150
200
250
300
350
400
Mass/Charge, Da

Figure S58
